# Toward Comprehensive Analysis of the 3D Chemistry of *Pseudomonas aeruginosa* Biofilms

**DOI:** 10.1021/acs.analchem.3c04443

**Published:** 2023-12-04

**Authors:** Anna M. Kotowska, Junting Zhang, Alessandro Carabelli, Julie Watts, Jonathan W. Aylott, Ian S. Gilmore, Paul Williams, David J. Scurr, Morgan R. Alexander

**Affiliations:** † School of Pharmacy, University of Nottingham, Nottingham NG7 2RD, U.K.; ‡ National Physical Laboratory, Hampton Road, Teddington, Middlesex TW11 0LW, U.K.; § National Biofilms Innovation Centre, Biodiscovery Institute and School of Life Sciences, University of Nottingham, University Park, Nottingham NG7 2RD, U.K.

## Abstract

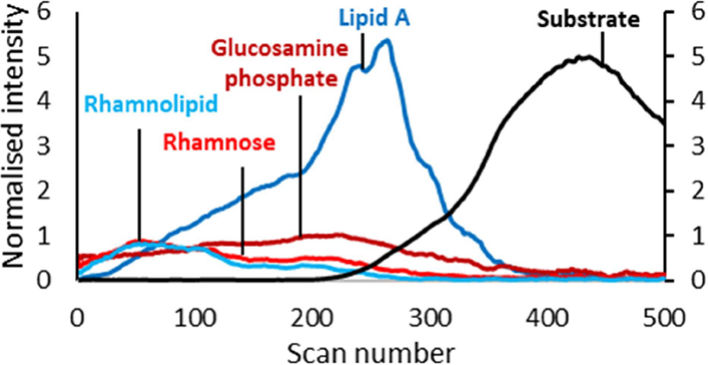

Bacterial biofilms are structured communities consisting of cells enmeshed in a self-generated extracellular matrix usually attached to a surface. They contain diverse classes of molecules including polysaccharides, lipids, proteins, nucleic acids, and diverse small organic molecules (primary and secondary metabolites) which are organized to optimize survival and facilitate dispersal to new colonization sites. In situ characterization of the chemical composition and structure of bacterial biofilms is necessary to fully understand their development on surfaces relevant to biofouling in health, industry, and the environment. Biofilm development has been extensively studied using confocal microscopy using targeted fluorescent labels providing important insights into the architecture of biofilms. Recently, cryopreparation has been used to undertake targeted in situ chemical characterization using Orbitrap secondary ion mass spectrometry (OrbiSIMS), providing a label-free method for imaging biofilms in their native state. Although the high mass resolution of OrbiSIMS enables more confident peak assignments, it is still very challenging to assign most of the peaks in the spectra due to complexity of SIMS spectra and lack of automatic peak assignment methods. Here, we analyze the same OrbiSIMS depth profile data generated from the frozen-hydrated biofilm, but employ a new untargeted chemical filtering process utilizing mass spectral databases to assign secondary ions to decipher the large number of fragments present in the SIMS spectra. To move towards comprehensive analysis of different chemistries in the sample, we apply a molecular formula prediction approach which putatively assigns 81% of peaks in the 3D OrbiSIMS depth profile analysis. This enables us to catalog over 1000 lipids and their fragments, 3500 protein fragments, 71 quorum sensing-related molecules (2-alkyl-4-quinolones and *N*-acylhomoserine lactones), 150 polysaccharide fragments, and glycolipids simultaneously from one data set and map these separated molecular classes spatially through a *Pseudomonas aeruginosa* biofilm. Assignment of different chemistries in this sample facilitates identification of differences between biofilms grown on biofilm-promoting and biofilm-resistant polymers.

## Introduction

Bacterial biofilms are communities of cells embedded in a self-generated extracellular matrix (ECM). These are found in many different environments, both natural and industrial including for example on food processing equipment, hospital water, and waste pipes, on plants and animals, for example on teeth, and also surfaces such as medical implants.^1^ After adhering irreversibly to a surface, bacteria organize into microcolonies prior to developing into mature biofilms which have properties substantially different from planktonic bacteria. The ECM in which the bacterial cells are embedded, consists mostly of polysaccharides, nucleic acids, lipids, proteins, and secondary metabolites. It also contains outer membrane vesicles and cell fragments as a consequence of the cell death and lysis that occur during biofilm development.^1^ The ECM offers protection for bacteria growing in biofilms from antibiotics and host immune defenses such that biofilms are commonly responsible for challenging chronic human infections.^2^ Consequently, the prevention and treatment of biofilm-associated infections require an in-depth understanding of bacterial biofilm formation, physiology, and architecture.

To date, a significant amount of information has been obtained on the composition and architecture of biofilms.^2^ Generally, biofilms are composed of clusters of cells, between which there are channels, transporting nutrients, secondary metabolites, and quorum sensing (QS) signaling molecules.^3^ QS signal molecules facilitate the cell density-dependent co-ordination of gene expression within a bacterial population. *Pseudomonas aeruginosa* for example employs both *N*-acylhomoserine lactones (AHLs) and 2-alkyl-4-quinolones (AQs) as QS signal molecules which collectively control the production of virulence determinants, motility, secondary metabolism, and biofilm maturation.^4^

Wide-ranging studies of the chemistry of *P. aeruginosa* biofilms have been done using LC-MS,^5^ providing chemically rich characterization, although this lacks the 3D information about molecule distribution throughout the biofilm. This is because the sample requires extraction from the surface, causing problems with the analysis of insoluble or volatile compounds,^6^ and this can be compounded by loss of material in the multistep sample preparation.

Several methods have been applied to analyze the chemistry of biofilms in situ, namely matrix-assisted laser desorption ionization (MALDI),^7^ secondary ion mass spectrometry (SIMS),^8,9^ and Raman spectroscopy.^10^ These studies have primarily focused on the detection and mapping of AQs such as 2-heptyl-3-hydroxy-4-quinolone (PQS) which contribute to biofilm development and aid communication between the cells and the environment. SIMS has also been applied to the analysis of biofilm matrix lipids and AQs,^11,12^ and peptide fragments have been detected using MALDI.^13^ Additionally, in situ analysis can help observe the impact of exogenous compounds and monitor the localization of signaling molecules.^14^ Recently, Zhang et al. developed a method for chemical imaging of native biofilms by using cryo-OrbiSIMS, revealing multiple QS molecules, lipids, amino acids, nucleobases and fatty acids, and other metabolites.^15^

One of the advantages of using label-free in situ techniques such as SIMS is the capability to carry out discovery-based research and detect several groups of compounds simultaneously; however, the complexity of the data often prevents comprehensive assignment of different chemistries. The introduction of the OrbiSIMS instrument,^16^ with higher mass resolving power and accuracy (Orbitrap analyzer) and less fragmentation (use of gas cluster ion beam as a primary beam), paved the way for more comprehensive data analysis. Recently, a chemical filtering methodology has been developed for 3D OrbiSIMS data by calculation of elemental formula from the exact mass.^17^ The program then uses the saturated or unsaturated character of the found molecules (double bond equivalent, DBE) to classify compounds into different compound categories, e.g., lipids, peptides, saccharides, and AQs. Here, we use this recently developed chemical filtering method (SIMS-Molecular Formula Prediction tool (SIMS-MFP)) to realize the full potential of the wealth of information achieved when investigating *P. aeruginosa* biofilm using cryo-OrbiSIMS.

## Experimental Section

### Sample Preparation

Part of this work is a reanalysis of data collected by Zhang et al. and detailed sample preparation information can be found in the referenced publication.^15^*P. aeruginosa* was maintained on lysogeny agar and grown overnight in lysogeny broth (LB) at 37 °C with constant shaking. For growth of biofilms, *P. aeruginosa* was grown overnight and diluted to an optical density at 600 nm (OD_600_) = 0.05 in FAB medium [2 g of (NH_4_)_2_SO_4_, 6 g of Na_2_HPO_4_·2H_2_O, 3 g of KH_2_PO_4_, 3 g of NaCl per liter] with 0.1 mM CaCl_2_, 1 mM MgCl_2_, 1 mL L^–1^ trace metals mix (200 mg L^–1^ CaSO_4_·2H_2_O, 200 mg L^–1^ FeSO_4_·7H_2_O, 20 mg L^–1^ MnSO_4_·H_2_O, 20 mg L^–1^ CuSO_4_·5H_2_O, 20 mg·L^–1^ ZnSO_4_·7H_2_O, 10 mg L^–1 CoSO4^·7H_2_O, 12 mg L^–1^ NaMoO_4_·H2O, and 5 mg L^–1^ H_3_BO_3_) and 30 mM glucose. Biofilms were directly grown on the flat face of 3 mm aluminum sample carriers for 48 h using a rotary flow system.^18^ Growth medium was replaced after 24 h. The fresh biofilms were washed 2–3 times with 150 mM ammonium formate solution and assembled in a sample carrier system for high-pressure freezing using a Leica EM ICE (Leica, Germany). After high-pressure freezing, samples were stored in liquid nitrogen.

Biofilms on EGdPEA and NGPDA polymers were grown on respective polymer-coated glass coverslips for 2, 5, and 24 h using a rotary flow system in FAB medium.^18^ The biofilms were washed three times with an ammonium formate solution (150 mM) and freeze-dried overnight before analysis.

### Cryo-OrbiSIMS Experimental Methods

The cryo-OrbiSIMS is equipped with a fully proportional–integral–derivative (PID) temperature controller, which controls resistive heating and a direct liquid nitrogen (LN_2_) closed loop circulation cooling stage, allowing sample temperature control within the load lock and main chamber. Having been installed with cryogenic storage tanks, LN_2_ was pumped for circulating the cooling medium through vacuum feed-throughs to a cooling finger below the sample, allowing fast cooling to −180 °C with a stability of ±1–2 °C for at least 7 days. This system allows for full sample movement in x, y, z, rotate, and tilt directions while under cryogenic conditions. Before measurement by cryo-OrbiSIMS, samples were placed in a cryo-manipulation station, Leica EM VCM (Leica, Germany), from where they were transferred to the cryo-OrbiSIMS using a shuttle chamber Leica EM VCT500 (Leica, Germany). The data were obtained from the University of Nottingham 3D OrbiSIMS (ToF-SIMS V Hybrid SIMS, IONTOF GmbH, Germany) and the process is described in detail in Supporting Information of Zhang et al., *Analytical Chemistry* 2020. All cryo-OrbiSIMS analyses (except those in Figure 2 and Supplementary Figure 3) were conducted at −180 °C. Mass calibration of the Q Exactive instrument was performed once a day using silver cluster ions. Electrons with an energy of 21 eV and a current of −10 μA and argon gas flooding were used for charge compensation. Three modes of OrbiSIMS were mainly used for the work described in this paper (details on the operation mode are given in ref (3)): mode 4 (single beam, 20 keV Ar_3000_^+^, Orbitrap MS). For all Orbitrap data, mass spectral information was collected from a mass range from 80 to 1200 Da. The Orbitrap analyzer was operated in positive-ion mode at the 240,000 at *m*/*z* 200 mass-resolution setting (512 ms transient time).

### Room Temperature OrbiSIMS Experimental Methods

The data were obtained from the University of Nottingham 3D OrbiSIMS (ToF5 Hybrid SIMS, IONTOF GmbH, Germany). Mass calibration of the Q Exactive instrument was performed once a day using silver cluster ions. Electrons with an energy of 21 eV and a current of −10 μA and argon gas flooding were used for charge compensation. Mode 4 (single beam, 20 keV Ar_3000_^+^, Orbitrap MS) was used for the measurement of the freeze-dried samples. Mass spectra were collected from a mass range from 75 to 1125 Da. The Orbitrap analyzer was operated in positive-ion mode at the 240,000 at *m*/*z* 200 mass-resolution setting (512 ms transient time).

Data analysis was done using the MFP software.^15^ The elemental and DBE limits are described separately in every compound group in the Results and Discussion section.

## Results and Discussion

Native *P. aeruginosa* biofilms were analyzed in cryogenic conditions as described previously by Zhang et al.^15^ in which a targeted assignment strategy was used to annotate approximately 100 peaks in the positive polarity spectra.^15^ We applied an untargeted approach to analyzing this data, starting with an automated peak search on the same OrbiSIMS positive polarity depth profile through the biofilm, resulting in identification of 9976 secondary ion peaks above the level of the noise. The manual assignment of molecules to this secondary ion data set would be challenging without the use of specialized software. This is especially true for fragments of macromolecules such as proteins and polysaccharides, which are typically of weak intensity and are often missed by manual analysis. Here, the chemical filtering (SIMS MFP^17^) enabled the assignment of 81% of all peaks in the positive ion spectrum with putative secondary ion compositions based on the accurate mass of the peaks at deviations <2 ppm (8104 of the total 9976 peaks).

**Figure 1:**
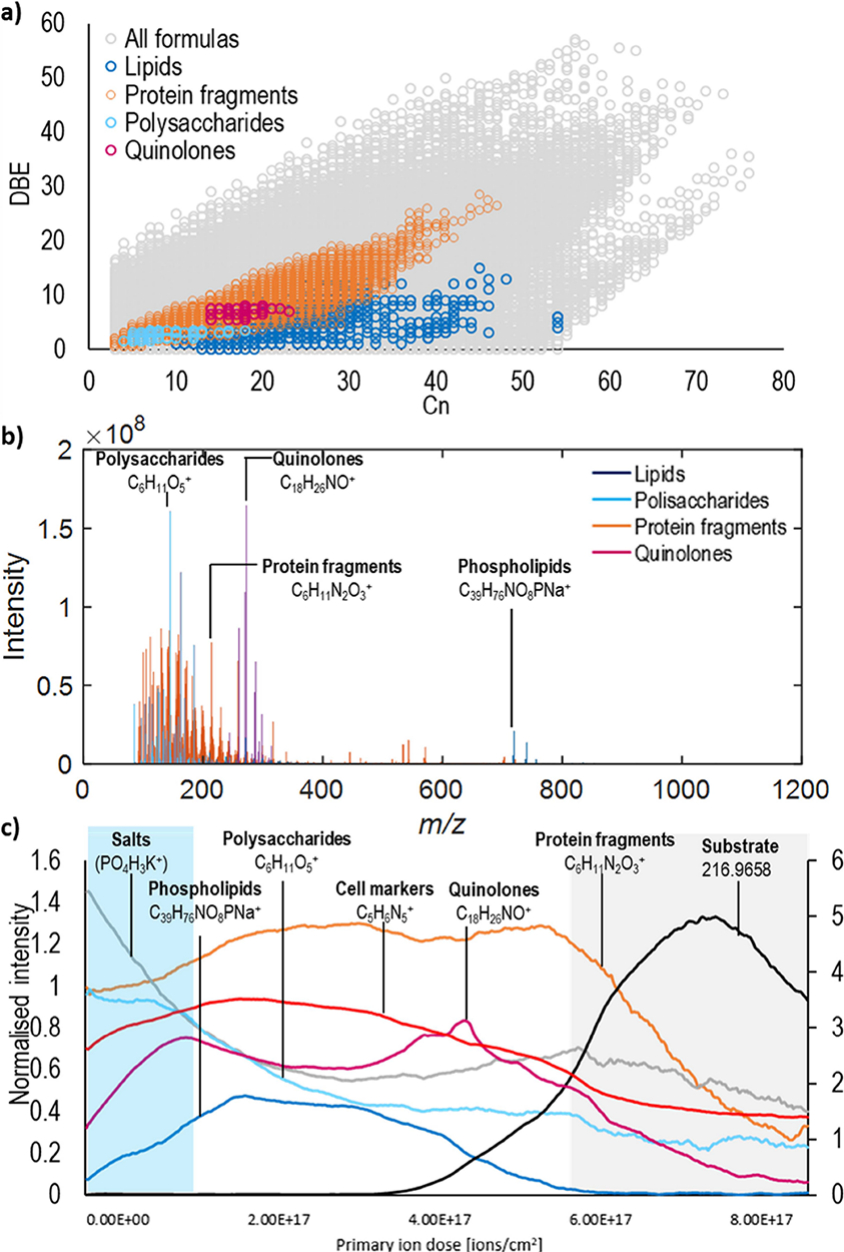
Groups of compounds detected in the OrbiSIMS depth profile analysis of frozen-hydrated *P. aeruginosa* biofilm. (a) All automatically calculated elemental formulas for ions detected in the spectrum can be divided into lipids, protein fragments, polysaccharides, and AQs based on the ratio of double bond equivalent (DBE) to the number of carbon atoms (Cn). (b) Full spectrum is automatically divided into spectra of different classes of compounds. (c) Different compounds can be tracked in the profile throughout the biofilm, shown here using individual ions of the class to which they belong. The intensity is normalized to total ion count. For visibility, the substrate marker (black) is presented on the secondary *y*-axis scale due to its high relative intensity.

The chemical filtering approach calculates possible chemical formulas based on elemental restrictions and enables rapid categorization of different chemistries within the secondary ion data, including that obtained from a depth profile analysis.^17^ To first limit the search to organic compounds within the software, elemental composition restrictions were applied for each secondary ion assignment: C_4–100_, H_8–200_, N_0–20_, O_0–20_, S_0–1_, Na_0–1_ K_0–1._ As a result, 342,314 possible formulas were produced for 8979 of the peaks (Figure 1a, gray) within the mass deviation of ±5 ppm below *m*/*z* 95 and ±2 ppm above *m*/*z* 95. The data are shown as a DBE number for the assignment plotted against the total carbon number in Figure 1a. DBE is a measure of degree of molecular unsaturation and is characteristic of different groups of compounds; for example, cyclic saccharides have higher DBE values at respective C_*n*_ (1:3) and aromatic heterocyclic quinolones are characterized by DBE/C_*n*_ of up to 1:1.5.

The resulting formulas were further filtered by known stoichiometries/ratios of elements for compound groups such as polysaccharides, AQs, salts, and substrate ions, as well as comparison with databases in the case of lipid (LIPIDMAPS^19^) and protein fragments,^17^ as shown in Figure 1a and described in the sections dedicated to each class. Representative ions of each of the assigned groups were highlighted in the OrbiSIMS spectra (Figure 1b) and depth profiles (Figure 1c) through frozen-hydrated *P. aeruginosa* biofilm, which follow the depth distribution of their class. Each of the chemical groups has a characteristic distribution throughout the biofilm, discussed in detail in the sections dedicated to each molecular class.

It has been found that optimization of the target potential for OrbiSIMS analysis has a significant effect on the peak intensity.^20^ In this work, the target potential was only optimized at the subsurface of the sample, but due to the complementary observations of correlated and anticorrelated ion intensity trends for different chemical species, we believe the actual changes observed in the depth profiles are caused by differences in the relative abundance of these compounds.

### Polysaccharides

Exopolysaccharides are largely responsible for the mechanical properties of the biofilm and form a protective layer for the cells.^21^ Depending on the *P. aeruginosa* strain, three different exopolysaccharides (alginate, Pel, or Psl) may be produced.^3^ Recent work by Khateb et al. has identified 17 polysaccharide ions in the OrbiSIMS spectra of *P. aeruginosa* biofilms expressing Psl and Pel.^22^ In the PAO1 strain used in this work, both Psl and Pel but not alginate are produced in biofilms.^23^

Several hexoses were detected in the biofilm spectra by setting the elemental limits and adding a restriction to H_2*n*_,O_*n*_ or H_2*n*+1_O_*n*_, 5 > *n* > 15 which is a typical saccharide composition. Glucose (C_6_H_13_O_6_^+^, C_6_H_12_O_6_Na^+^), rhamnose and rhamnose phosphate (C_6_H_11_O_5_Na^+^, C_6_H_11_O_5_PO_3_^+^), and 92 polysaccharide fragments (two, three, four-membered saccharides) were automatically assigned, including Rha-Rha (C_12_H_20_O_8_Na^+^) chains (Table S1), which are present as rhamnolipid components as shown in the following glycolipid section. In addition, rhamnose is found in the pentasaccharide repeat unit of the Psl exopolysaccharide and as a homopolymer in the *P. aeruginosa* lipopolysaccharide (LPS) common polysaccharide antigen. The depth profile data for these ions within the biofilm suggests that the monosaccharides are found in the outermost layer of the biofilm while the larger structures appear consistently throughout the depth of the sample (Supplementary Figure S1). The presence of the monosaccharides in the outer layer of the biofilm may be caused by diffusion of these compounds from lysed bacterial cells or the compounds remaining in the sample after culturing in glucose-supplemented growth media.

### Lipids

The elemental formulas assigned on the basis of accurate mass comparison were matched with the LIPIDMAPS database,^19^ linked to the SIMS-MFP process. Using this methodology, 1152 lipid species were assigned and separated into different lipid classes, including [M + H]^+^, [M + Na]^+^, and [M + K]^+^ ions (Table S2). The most abundant lipid ions were assigned as fatty acid fragment ions, intact phospholipids, and diradylglycerols. The depth profile of representative ions of these groups, separated into protonated ions, sodium adducts, and potassium adducts is presented in Supplementary Figure S2. Protonated ions and sodium adducts assigned as lipids were detected throughout the biofilm, with phospholipids being more prevalent in the upper part of the bulk of the biofilm and fatty acids present closer to the interface with the substrate (Supplementary Figure S2a and b). Potassium adducts of lipids were present in the bulk of the biofilm (Supplementary Figure S2c).

### Glycolipids

Glycolipids are an important family of virulence factors, conjugates of lipids, and polysaccharides, such as LPS and the rhamnolipids.^24^ Intact bacterial LPSs are macromolecules of molecular masses 10–20 kDa made up of three structural components. Lipid A, consisting of diglucosamine phosphate, O- and N-linked primary and secondary fatty acids, a core polysaccharide chain, and a repeating hydrophilic O-antigen oligosaccharide side chain that is either specific to the O serotype of the strain [serotype O5 for PAO1 where the repeating unit is → 4)-dManNAc3NAmA-(β1 → 4)-d-ManNAc3NAcA-(β1 → 3)-d-FucNAc-(1 → )] or is a rhamnose homopolymer.

**Figure 2:**
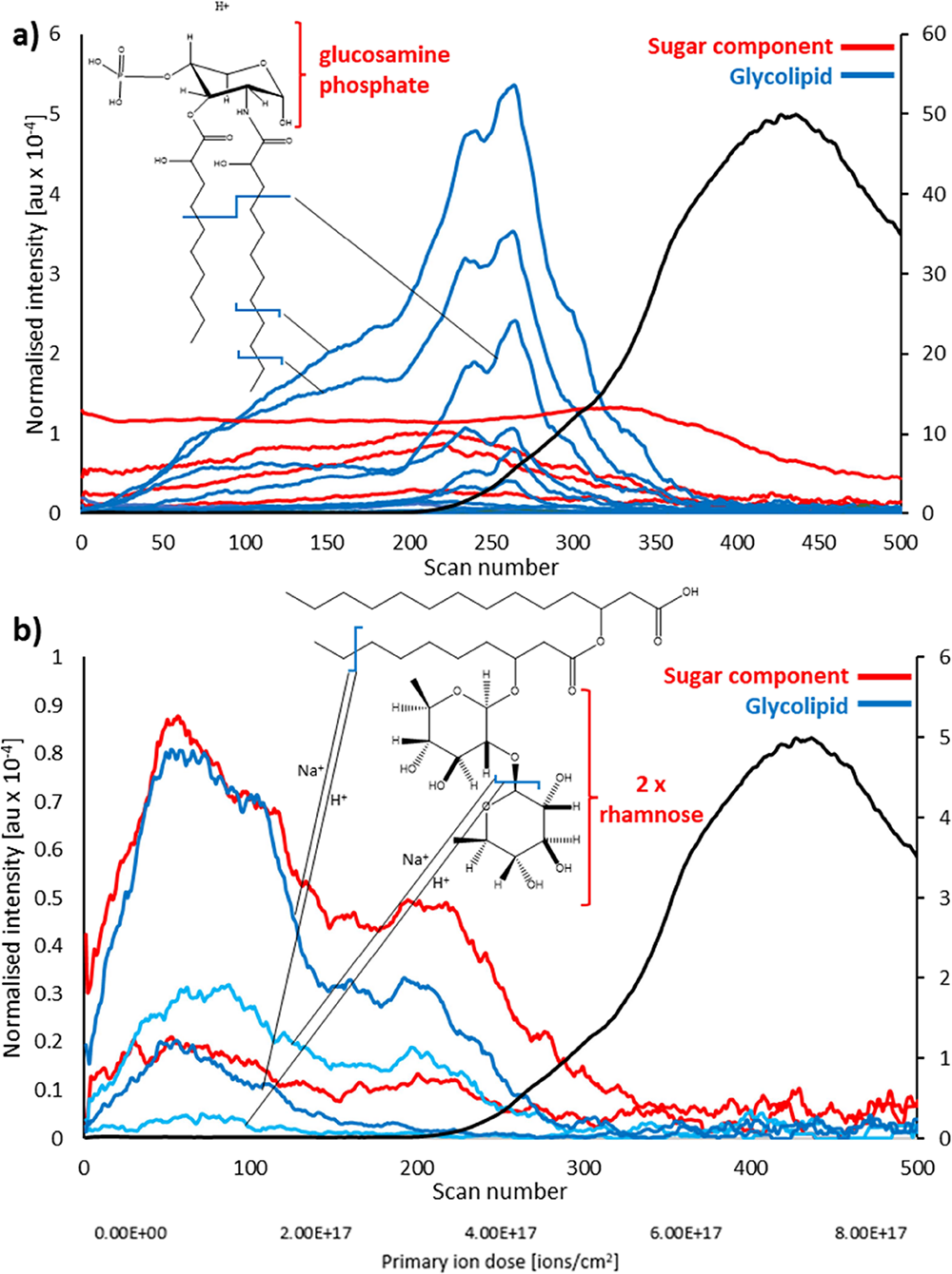
OrbiSIMS depth profile of *P. aeruginosa* biofilm highlighting the glycolipids. (a) Lipid A fragments. Glucosamine with fatty acids: 543.2797 C_23_H_46_NO_11_P^+^, 571.3110 C_25_H_50_NO_11_P^+^, 599.3422 C_27_H_54_NO_11_P^+^, and glucosamine phosphate fragments 230.0423 C_5_H_13_NO_7_P^+^ were assigned. (b) Rhamnolipid fragments, rhamnose fragments (C_12_H_21_O_8_^+^), and rhamnolipid fragments (C_26_H_48_NaO_9_^+^, C_34_H_63_O_13_^+^, C_34_H_62_NaO_13_^+^) are detected under the outermost layer of the biofilm. The intensity is normalized to total ion count. For visibility, the substrate marker (black) is presented on secondary *y*-axis scale due to its high relative intensity.

The hydrophobic lipid A moiety anchors LPS within the outer membrane.^25^ Setting the elemental composition limits to a range that would include glycolipids, C_4–100_, H_8–200_, N_0–1_, O_0–20_, P_0–1_, S_0–0_, Na_0–1_, revealed a range of ions, which have a maximum intensity at the interface between the biofilm and the substrate and appear as a double peak in the depth profile (Figure 2a, blue, glycolipid fragments). This suggests that LPS, a bacterial outer membrane component, is preferentially located in areas of the thick biofilm containing cells and outer membrane vesicles. The ions with this distribution were assigned as glucosamine phosphate and associated lipid A fragments and are found in Table S1.

Rhamnolipids are glycolipids that are involved in maintaining biofilm architecture; however, they also disrupt cell-to-cell and cell-to-surface interactions and contribute to maintaining the channels formed in a biofilm to enable the flow of molecules, water, and oxygen.^26^

In this work, rhamnolipids were assigned in the spectra by searching for structures with additional C_*n*_, elongating the alkyl chain from previously assigned rhamnose ions. As a result, 64 rhamnolipid ions, including phosphorylated ions and sodium adducts, were assigned. Representative ions are shown in the depth profile through the biofilm in Figure 2b, and all assignments are available in Table S1. The depth profile revealed the presence of rhamnolipids toward the surface of the biofilm, declining steadily into the depth of the biofilm. This is consistent with the formation of a rhamnolipid “shield” on the biofilm outer surface that during infection protects biofilm bacteria from host phagocytic cells by inducing their necrotic killing.^27^

### QS Molecules

*P. aeruginosa* produces over 50 distinct AQs compounds^24,28^ and two major AHLs which play different roles in the biofilm, from co-ordinating gene expression leading to changes in biofilm architecture, fitness, community protection, and resistance to environmental stress.^29,30^ By applying further composition restriction of C_4–30_, H_8–200_, N_0–2_, O_0–2_, S_0–0_, Na_0–1_ K_0–1_, and DBE 6–20, we achieved automatic assignment of 54 AQ-associated ions, which represent 2-hydroxy-4-alkylquinolines and 2-alkyl-3-hydroxy-4-quinolones with different alkyl chain lengths, as summarized in Table S3. All ions assigned as quinolones follow the same trend in the depth profile, suggesting that they are present throughout the whole biofilm, most prevalent within the bulk region and on the interface between the cells and the substrate (Supplementary Figure S3). The maximum intensity of the quinolone signal correlated in depth with the maximum intensity of the lipid A ions. This is consistent with the known physical interactions of PQS with the acyl chains and 4′-phosphate of lipid A, which drive the formation and release of outer membrane vesicles into the ECM.^31^

### Salts

The samples were washed with ammonium formate immediately prior to high-pressure freezing to reduce the contribution of salts and the associated matrix effect; however, salts were still detected in the depth profile. These were mainly phosphate salts, sodium, potassium, or ammonium adducts, and they were assigned automatically by searching ions of possible composition C_0_, H_1–20_, N_0–1_, O_0–20_, S_0–0_, Na_0–5_, P_0–5_, K_0–5_ in the spectrum (Table S5). The salts were most prevalent at the very top surface of the biofilm; however, they were generally detected throughout the depth of the biofilm (Supplementary Figure 4). This suggests that the equilibrium level of salts in the biofilm is lower than in the growth medium.

### Protein Fragments

Proteins are an important component of the ECM as well as the bacterial cells, performing functions ranging from biofilm formation and initiation of host immune response,^32^ signaling, maintaining the structure of the biofilm, and responding to stress.^33^

The recently developed method of analyzing intact proteins using OrbiSIMS by de novo sequencing^34^ could enable label-free mapping of macromolecules alongside other components of the biofilm. However, selection and assignment of peptide and protein fragments in complex samples are not readily possible due to these molecules producing large numbers of peptide ions with low intensity of the informative high mass ions. Here, we applied a chemical filtering process to isolate protein-related secondary ions to aid the assignment of peptide and protein fragments in biofilms. The chemically filtered list of organic molecules assigned as peptides, presented in Figure 1a, was matched with a database of theoretical formulas for up to six-membered peptides.^17^ A total of 3637 peaks in the *P. aeruginosa* spectrum matched the formulas of protonated or sodiated peptide ions. These protein fragment peaks were detected in the bulk of the biofilm, including the interface between the biofilm and the substrate (Supplementary Figure S5). Due to the large number of peptide fragments and complex character of the mixture, it is not possible at this stage to confidently identify specific proteins in the biofilm.

### Metabolites and Other Compounds

**Figure 3:**
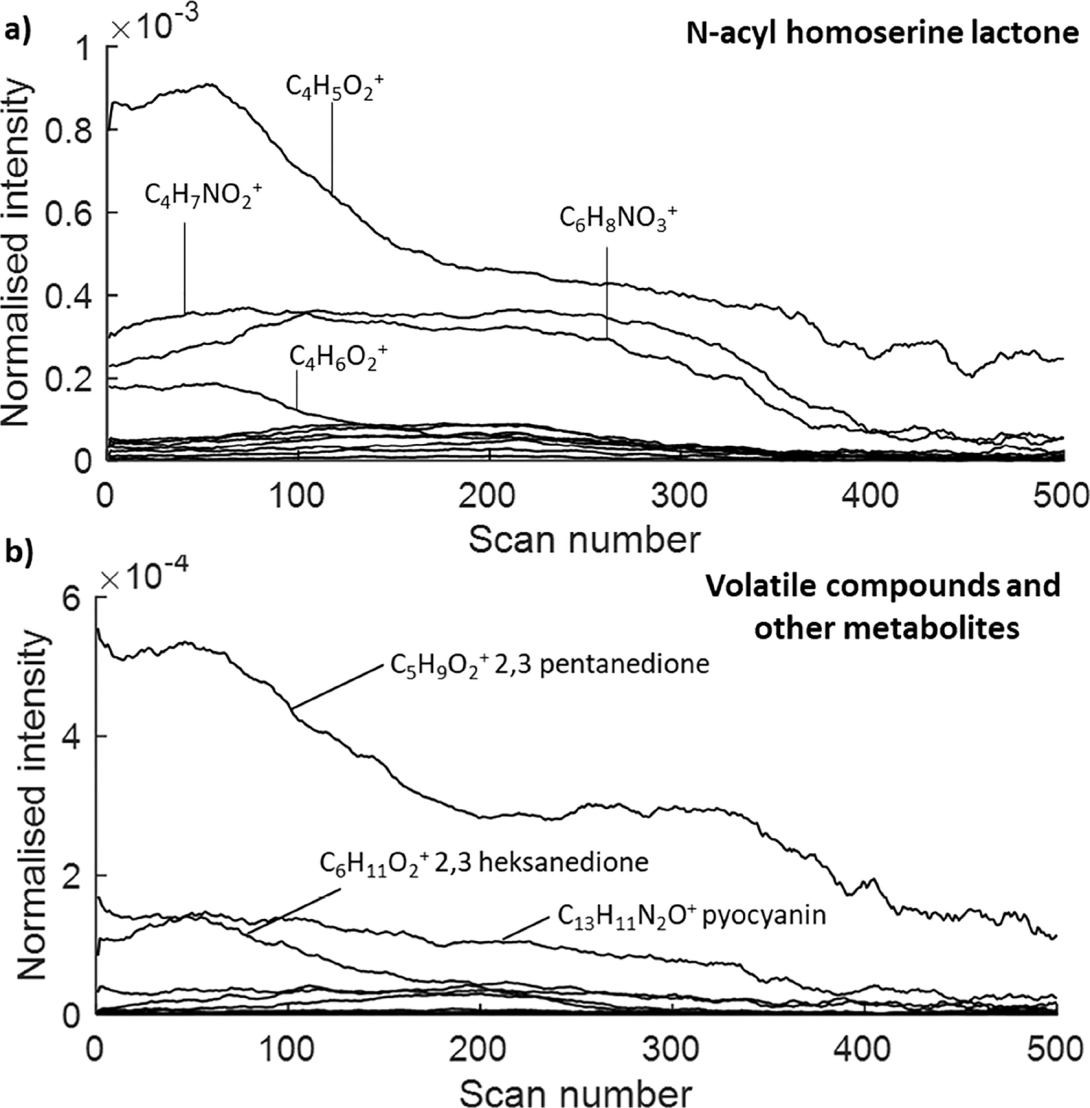
Overview of depth distribution of (a) *N*-acyl homoserine lactone (AHL) fragments and (b) volatile compounds and other metabolites assigned in the frozen hydrated biofilm.

The chemical filtering process allowed the cataloging of major classes of compounds present in the biofilms; however, several metabolites may be missed in this process due to not belonging to a specific chemical category. For example, AHL-type QS signal molecules were detected in the spectra with 13 fragments summarized in Table S4 and presented in the depth profile in Figure 3a. In contrast to quinolones, the AHLs are detected toward the surface of the biofilm rather than in the bulk. Based on LC-MS metabolomics analysis, aside from rhamnolipids and AQs, molecules contributing to the pathogenicity of *P. aeruginosa* include the QS-regulated secondary metabolites: pyocyanin C_13_H_11_N_2_O^+^, the pyocyanin precursor phenazine–1–carboxylic acid C_13_H_8_N_2_O_2_Na^+^,^27^ and the siderophore pyochelin C_14_H_16_N_2_O_3_S_2_^+^,^35^ all of which were detected in the OrbiSIMS data with their presence observed throughout the biofilm shown in the Supplementary Figure S6a. Cryo-OrbiSIMS has been found to enable assignment of volatile molecules^36^ and here 2-aminoacetophenone, 2,3-hexanedione, 2,3-pentanedione, and 2-decanone were assigned for *P. aeruginosa*. These are presented in the Supporting information and are mapped in the biofilm as shown in Figure 3b.

### Remaining Peaks

After automatic assignment of the listed groups of compounds, 1872 peaks remained unexplained in the spectrum, meaning that SIMS-MFP enabled automatic assignment of 81% of the peaks in the frozen-hydrated biofilm by selecting elemental composition and DBE values for groups such as lipids, proteins, saccharides. Importantly, OrbiSIMS allowed the assignment of these different chemistries simultaneously from one data set. Additionally, a total of 236 peaks were assigned as generic organic fragments such as C_21_H_38_N^+^, C_23_H_42_N^+^, C_4_H_7_N_2_^+^, C_4_H_9_N_2_^+^ (Supplementary Figure S6b). These structures cannot be said to originate uniquely from one specific compound or group of compounds.^32^ The unexplained peaks are shown with suggested assignments in an overlay comparison of the original and remaining spectrum (Supplementary Figure S7a) and the depth profile (Supplementary Figure S7b). Several unexplained peaks are detected in the surface, bulk biofilm, and the substrate section of the depth profile (Supplementary Figure S7b).

### Comparison of a Frozen-Hydrated and Freeze-Dried Biofilm

**Figure 4:**
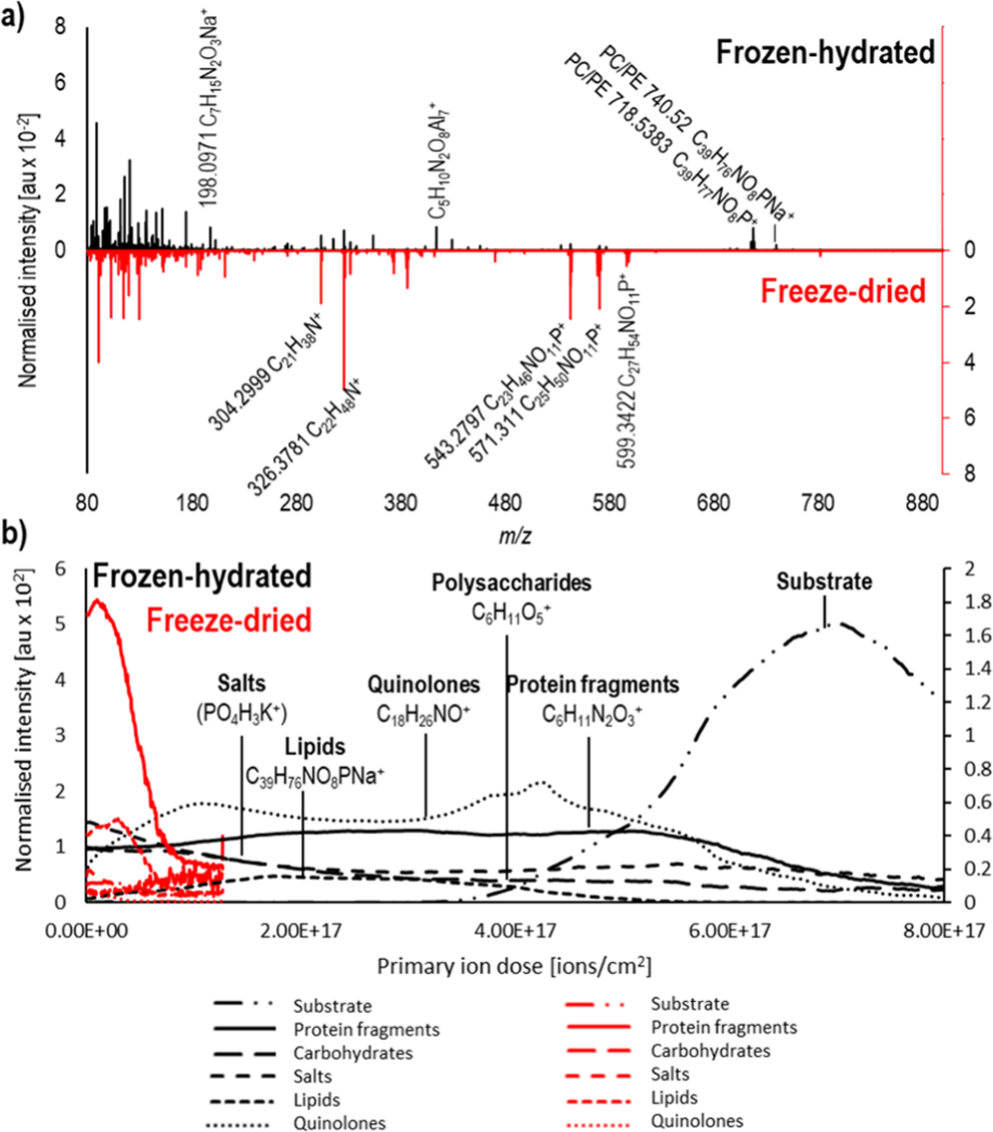
Comparison of (a) spectra and (b) depth profiles of frozen-hydrated (black) and freeze-dried (red) *P. aeruginosa* biofilm. Representative ions from different classes of compounds, lipids, protein fragments, polysaccharides, quinolones, salts, and substrate ions are mapped throughout the biofilm. The intensity is normalized to total ion count. For visibility, the substrate marker is presented on secondary *y*-axis scale due to its high relative intensity.

Zhang et al. demonstrated that the ions assigned in the frozen-hydrated biofilm are of higher intensity than in the spectrum of the same sample after freeze-drying.^15^ Here, we extended that observation by the automatic assignment and mapping of representative ions of assigned chemicals. The majority of the peaks (9704 of 9976 peaks) are more intense in the frozen-hydrated sample (Figure 4a, Supplementary Figure 8), whereas only 272 peaks have higher intensity in the freeze-dried sample. These latter ions belong to the ions designated as salts, glycolipid ions, sodium adducts of protein fragments, and generic organic fragments (such as C_22_H_48_N^+^). A comparison of the depth profiles through the frozen-hydrated and freeze-dried samples shows the physical collapse of the biofilm after freeze-drying reflected in the rapid appearance of the substrate in the profile (Figure 4b). This agrees with the original finding of Zhang et al. that that the frozen hydrated state is the more suitable method for analyzing intact biofilms.

### Comprehensive Analysis of Biofilm Development on Polymers Designed To Modulate Biofilm Formation

By applying the chemical filtering approach, we putatively assigned 81% of peaks in the OrbiSIMS depth profile analysis of a model biofilm sample. This analytical approach aims to guide analysis of more challenging, real-world samples designed to control biofilm in applications such as bioreactors and medical devices. To illustrate its utility, we grew *P. aeruginosa* on two polymers identified as probiofilm (neopentyl glycol diacrylate, NGPDA) and antibiofilm (ethylene glycol dicyclopentenyl ether acrylate, EGdPEA).^37^ The polymer surfaces were sampled at three time points 2, 5, and 24 h post-inoculation to investigate the chemical differences apparent during early-stage biofilm formation which may be key to its subsequent maturation, and the samples were freeze-dried before the analysis. To analyze the OrbiSIMS data, we created a peak list of 3280 peaks by combining the peaks detected on NGPDA and EGdPEA polymers (without exposure to bacteria), and the peaks detected at each exposure time (2, 5, and 24 h). Using the SIMS-MFP approach, we assigned 2131 of these peaks as polymer related, leaving 1149 peaks that are putatively biofilm related. In the sample expected to contain the greatest number of bacteria, i.e., 24 h biofilm on the probiofilm, NGPDA, we assigned 82 peaks as saccharide/polysaccharide related, 29 lipid peaks, 125 salt ions, 40 protein fragments, 60 quinolone peaks, and 68 nonspecific organic fragments such as C_21_H_38_N^+^ (Tables S7–S10).

The spectra of all samples were dominated by polymer-related peaks and as the thickness of the biofilm is different at 2, 5, and 24 h as well as between the biofilms grown on EGdPEA and NGPDA. Statistical analysis of the entire data set reflected mainly these differences in biofilm thickness (Supplementary Figure S9). SIMS-MFP enabled filtering of highly prevalent substrate ions and the analysis of differences in the particular chemical classes.

**Figure 5:**
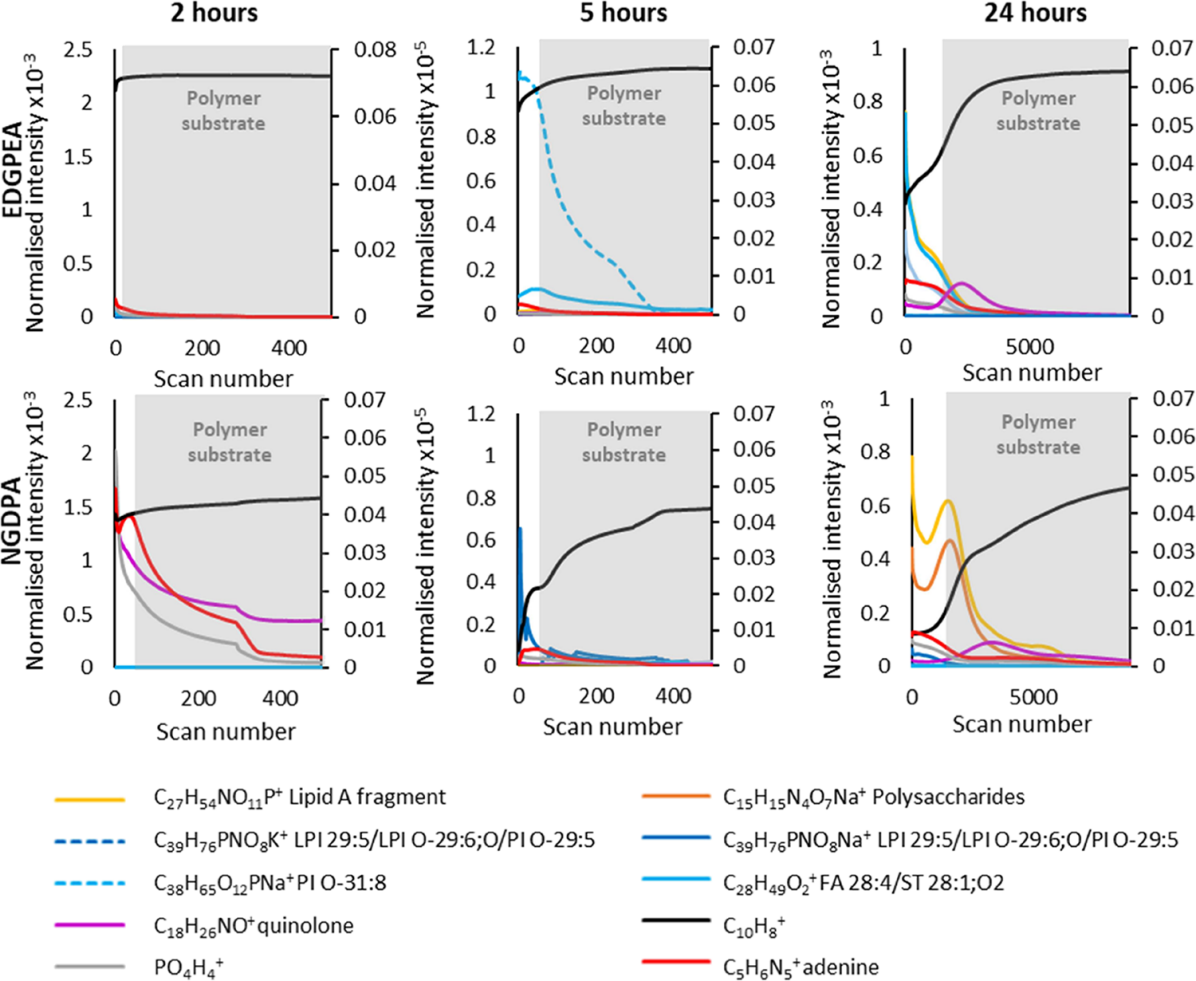
Depth profiles of example chemistries throughout different time points in biofilm formation on EGDPEA and NGPDA polymers. The intensity is normalized to total ion count. For visibility, the C_10_H_8_^+^ ion (polymer substrate marker) is presented on a secondary *y*-axis scale due to its high relative intensity. Note the expansion of the scale at 24 h to accomodate the thicker biofilms.

Principal component analysis (PCA) of the biofilm-related peaks revealed different chemistries of the biofilms grown on NGPDA and EGdPEA and can provide insights into why biofilms form preferentially on the first material and not the latter. Example ions assigned as differences between the samples are presented as depth profiles in Figure 5. It is clear that at every time point, the biofilm growing on the biofilm-promoting material (NGPDA) has more visible cell marker signal (red, adenine, C_5_H_6_N_5_^+^) and quinolone signal (purple, C_38_H_26_NO^+^), while the time taken to reach the polymer marker (black, C_10_H_8_^+^) indicates the increased thickness of the biofilm (Figure 5). Importantly, PCA also revealed differences in chemical classes present on the surface (Supplementary Figure S9). At all time points, the EGdPEA biofilm spectrum contained lipid peaks: C_38_H_65_O_12_PNa^+^ (PI O-31:8) and C_28_H_49_O_2_^+^ (FA 28:4/ST 28:1;O2), which were absent in polymer reference samples and all NGPDA biofilm samples (Figure 5, light blue lines, Supplementary Figure S10). This difference was the most visible at 5 h of biofilm growth. Conversely, the biofilm growing on NGPDA contained C_39_H_76_PNO_8_Na^+^ (PC 31:1/PE 34:1), which was completely absent in all EGdPEA biofilm samples (Figure 5, dark blue lines). Only at 24 h of biofilm growth, lipid A fragments (yellow) and polysaccharide fragments (orange) were starting to be visible on the NGPDA sample. This difference in the chemical composition of the biofilm on EGdPEA and NGPDA may be used to understand the difference in the architecture of the biofilms formed on different materials.^27^

## Conclusions

OrbiSIMS together with the chemical filtering methodology enabled rapid identification and mapping of different chemistries simultaneously in *P. aeruginosa* biofilms, achieving putative structural assignment and classification into chemical classes of 81% of all secondary ions detected. The MFP chemical filtering analysis enabled assignment of polysaccharides, LPSs, and potential protein and peptide fragments alongside routinely assigned AQs and lipids. A total of 1152 ions were putatively assigned as lipids or lipid fragments and separated into lipid classes, with the most abundant being fatty acids, phospholipids, and diradylglycerols. Some 3637 protein fragment ions were characterized, although the method was not able to ascribe them to specific proteins with any degree of certainty due to different protein fragments having similar elemental compositions. Sputter profiling through the frozen-hydrated sample enabled mapping of the molecules in depth (*z*); however, it did not provide information about *x*–*y* heterogeneities of the biofilm. Comparison of frozen-hydrated and freeze-dried spectra and depth profiles of the biofilm confirmed previous observations that the hydrated state enhances the signal of most molecules; however, several chemistries are more prevalent in the freeze-dried sample.

Chemical filtering allowed the assignment of complex samples such as biofilms grown on real-world polymers applied to control infection and revealed changes in the biological composition of the samples grown on biofilm-promoting versus biofilm-preventing materials. This study presents the capability to simultaneously characterize different chemistries in situ in a complex sample using OrbiSIMS and the approach could be applied to providing new insights into the formation and maturation of biofilms and their responses to environmental stresses.

## Supporting Information Available

Supporting Information Available: [Supplementary figures and table and data can be found at https://rdmc.nottingham.ac.uk/.]
Depth profiles of automatically assigned saccharides; depth profile of lipids throughout the frozen-hydrated biofilm; depth profile of quinolones throughout the frozen-hydrated biofilm; depth profile of salt ions throughout the frozen-hydrated biofilm; distribution of protein fragments throughout the biofilm; unassigned ions in the frozen-hydrated biofilm sample; comparison of normalized intensities of identified groups of compounds in cryogenic conditions and freeze-dried sample; principal component analysis results; substrate ion assignments in the biofilm spectrum; quinolone assignments in the biofilm spectrum (PDF)
