# Supplementary material for: Toward Comprehensive Analysis of the 3D Chemistry of Pseudomonas aeruginosa Biofilms
Source: Anal Chem. 2023 Dec 4;95(49):18287–94. doi: 10.1021/acs.analchem.3c04443 (PMC10719885; doi:10.1021/acs.analchem.3c04443)
Supplement: Supplementary file 1 — ac3c04443_si_001.pdf [file ac3c04443_si_001.pdf]

Supplementary information for:

Towards comprehensive analysis of the 3D chemistry of *Pseudomonas aeruginosa* biofilms

Anna M. Kotowska[1], Junting Zhang[2], Alessandro Carabelli[1], Julie Watts[1], Jonathan W. Aylott[1], Ian S. Gilmore[2], Paul Williams[3], David J. Scurr[1], Morgan R. Alexander[1*]

*morgan.alexander@nottingham.ac.uk

[1] *School of Pharmacy, University of Nottingham, Nottingham, NG7 2RD, UK*

[2]*National Physical Laboratory, Hampton Road, Teddington, Middlesex, TW11 0LW, UK*

[3] *National Biofilms Innovation Centre, Biodiscovery Institute and School of Life Sciences, University of Nottingham, University Park, Nottingham NG7 2RD, U.K.*

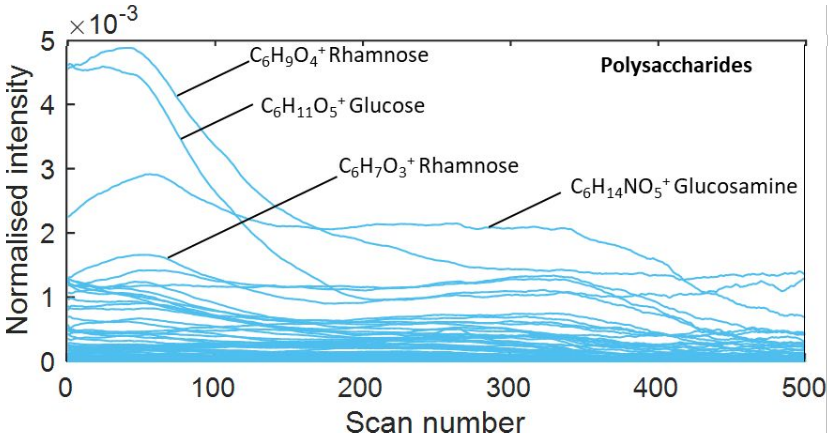

Supplementary Figure 1. Depth profiles of automatically assigned saccharides, including mono and polysaccharides

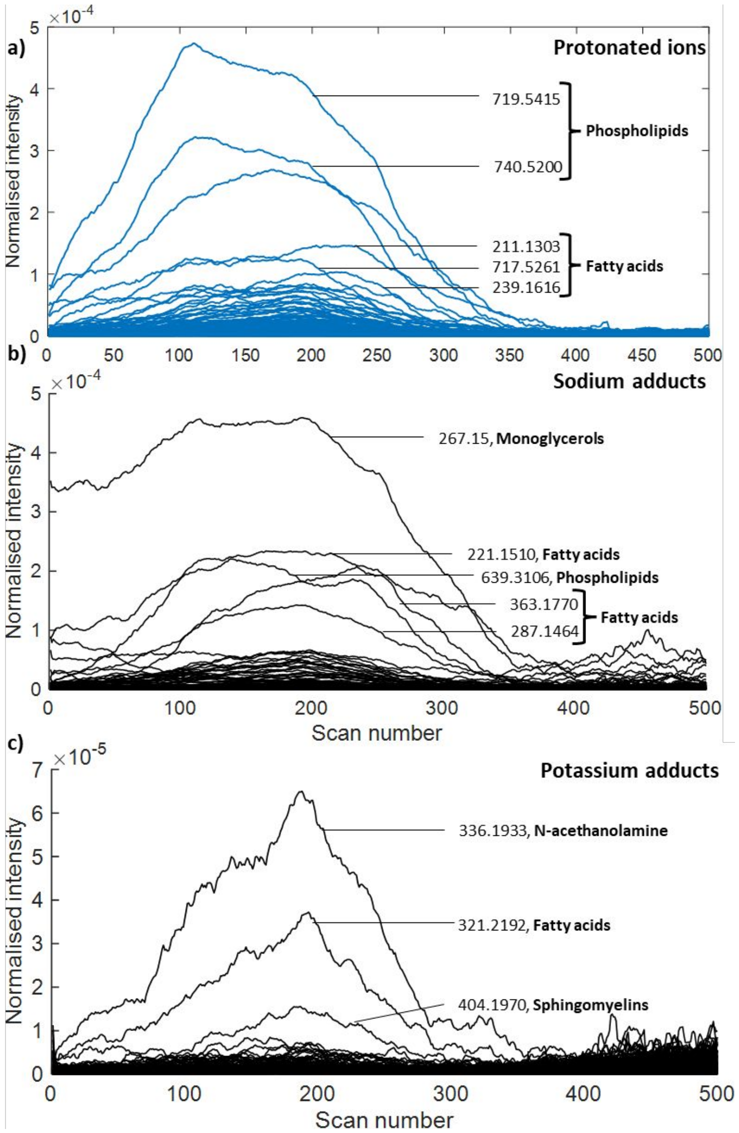

Supplementary Figure 2. Depth profile of lipids throughout the frozen-hydrated biofilm, identified as a) protonated ions, b) sodium adducts and c) potassium adducts.

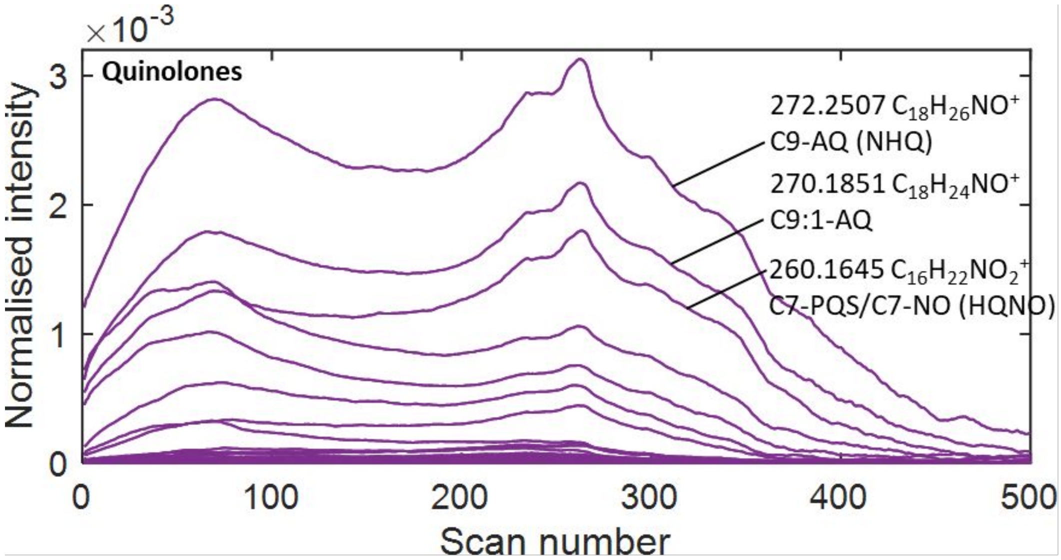

Supplementary Figure 3. Depth profile of quinolones throughout the frozen-hydrated biofilm.

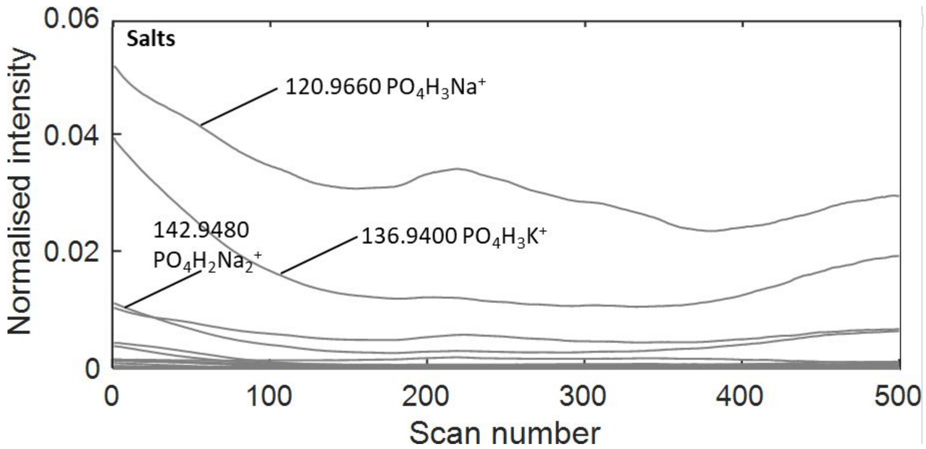

Supplementary Figure 4. Depth profile of salt ions throughout the frozen-hydrated biofilm.

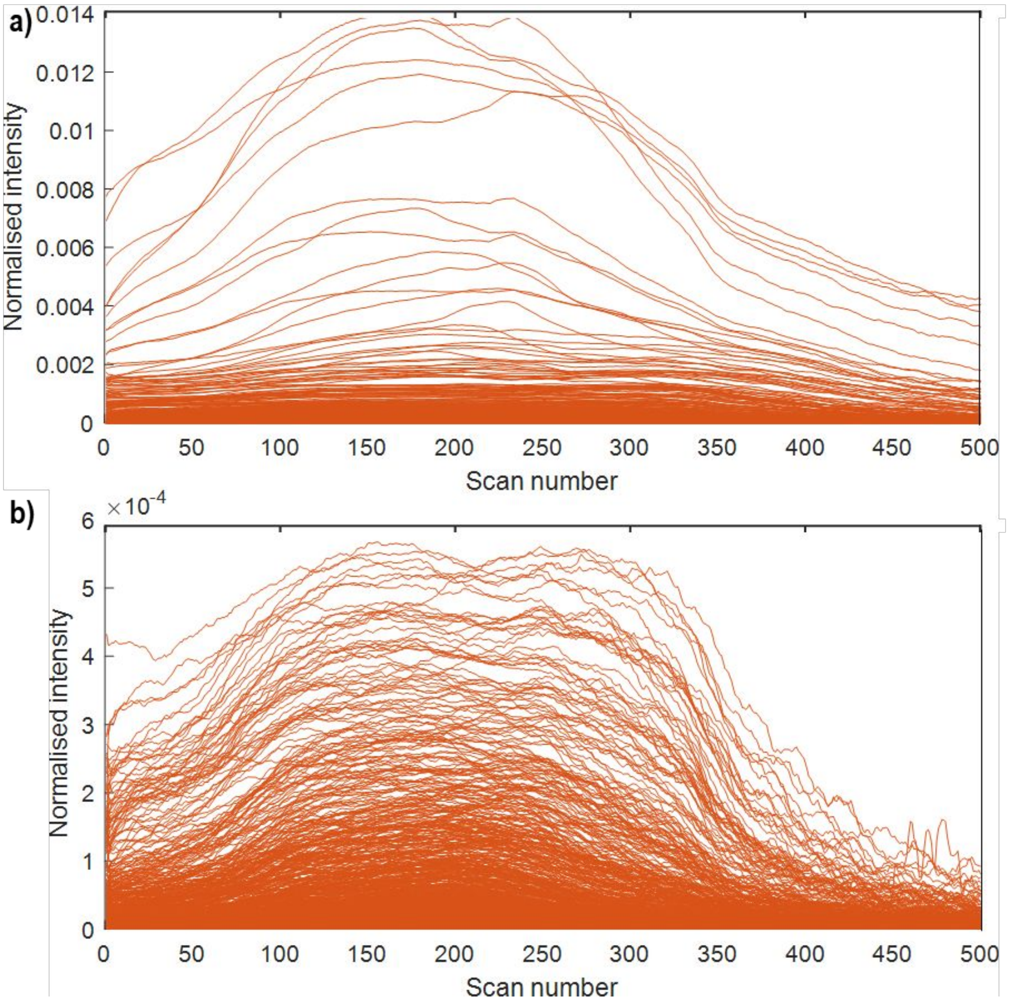

Supplementary Figure 5. Distribution of protein fragments throughout the biofilm. a) high intensity single amino acid ions and b) lower intensity ions.

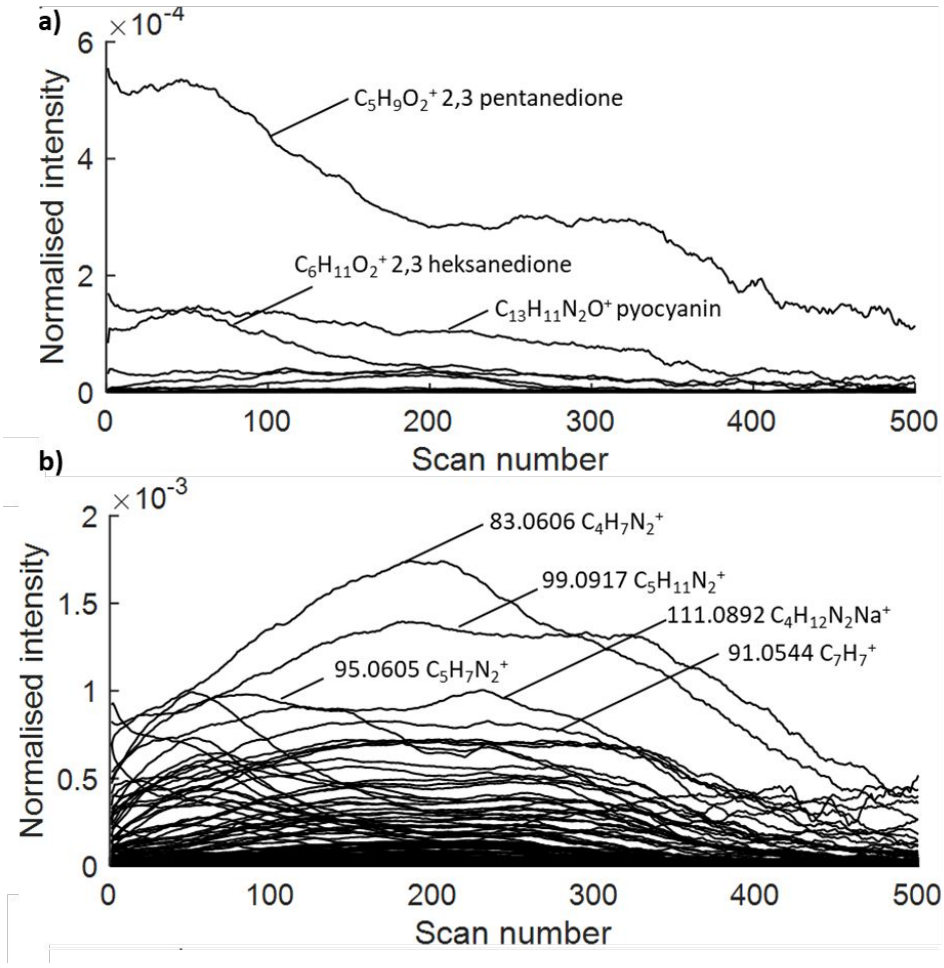

Supplementary Figure 6. Depth profiles of **a)** miscellaneous compounds assigned manually as known *P. aeruginosa* metabolome/volatilome and **b)** generic organic fragments consisting of C,H,N,Na.

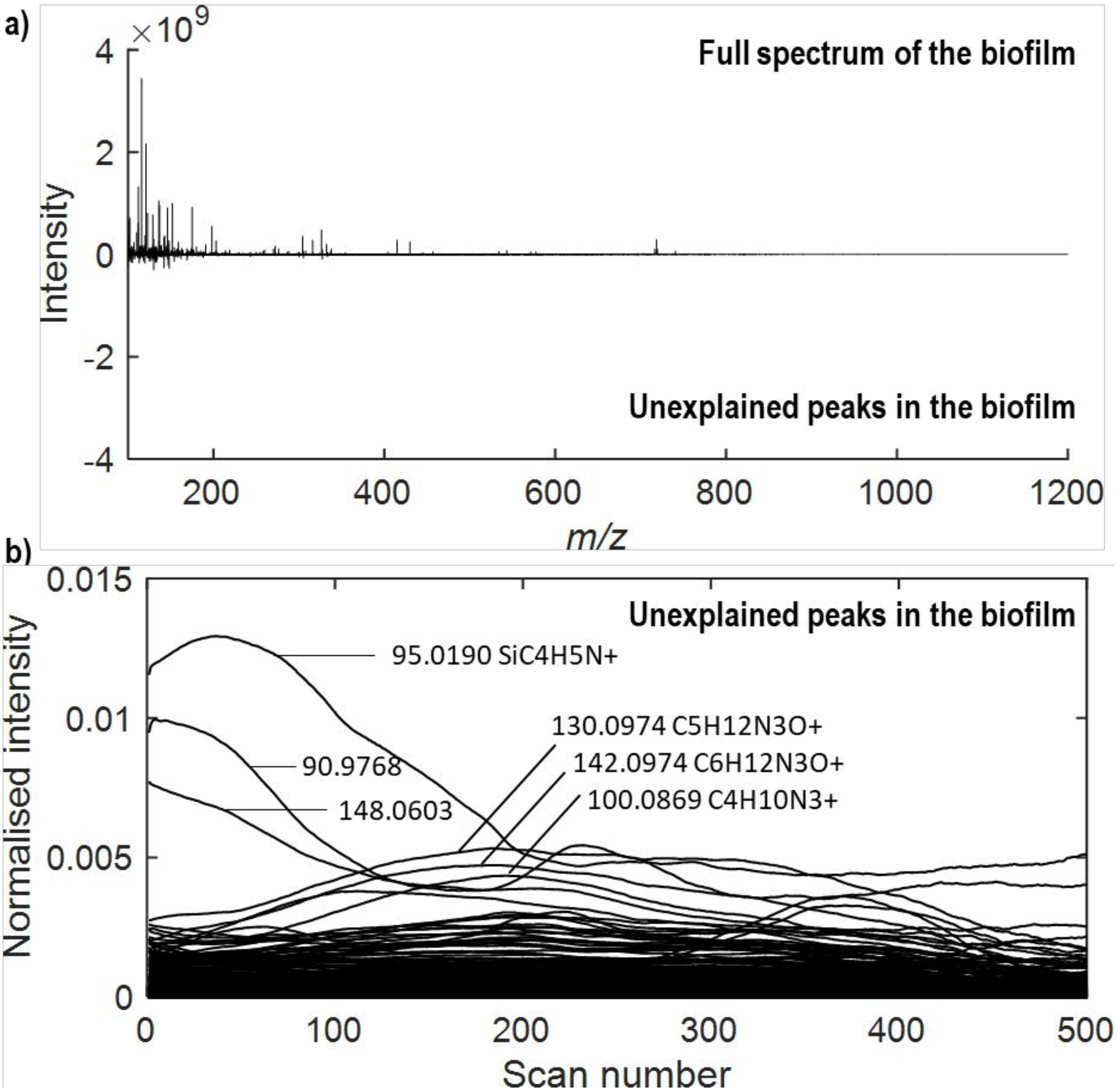

Supplementary Figure 7. Unassigned ions in the frozen-hydrated biofilm sample, shown as a) comparison of full and unassigned spectra and b) depth profile of unassigned peaks.

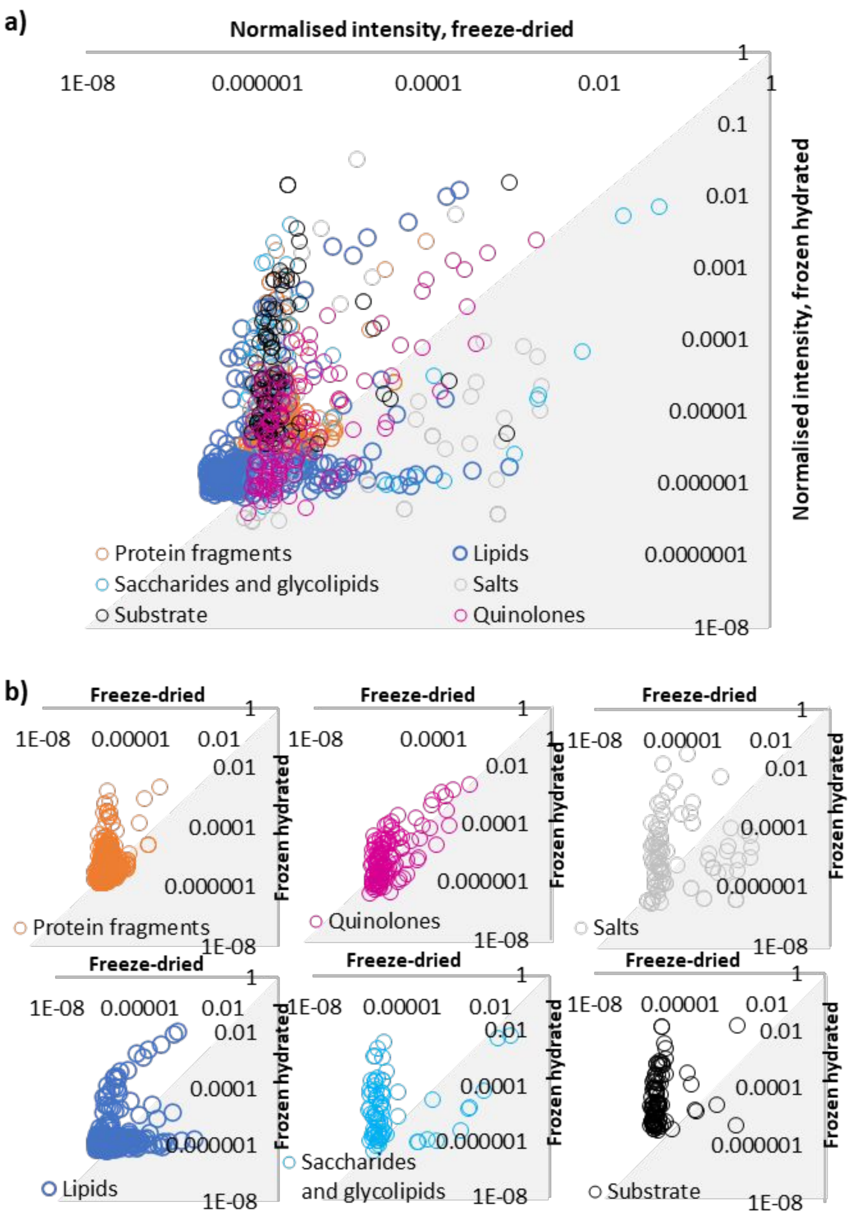

Supplementary Figure 8. Comparison of normalised intensities of identified groups of compounds in cryogenic conditions and freeze-dried sample. a) overlay of major identified groups of compounds with compounds with higher intensities in frozen hydrated state presented on white background, compounds with higher intensities in room temperature presented on grey background. b) Different groups of compounds separated for clarity.

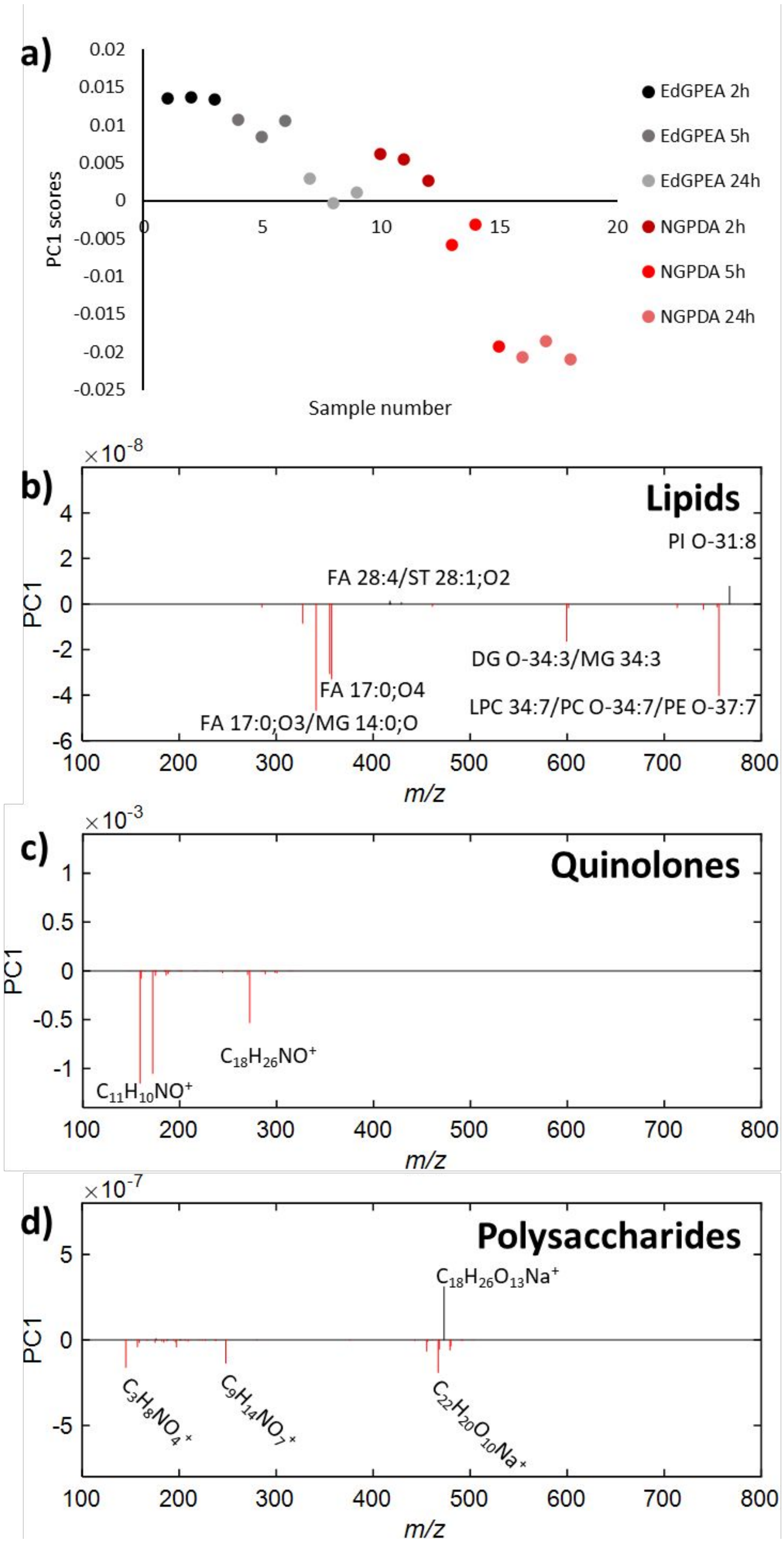

Supplementary Figure 9. Principal component analysis results. a) scores of PC1 b) loadings of PC1 assigned as lipids, c) loadings of PC1 assigned as quinolones, d) loadings of PC1 assigned as polysaccharides.

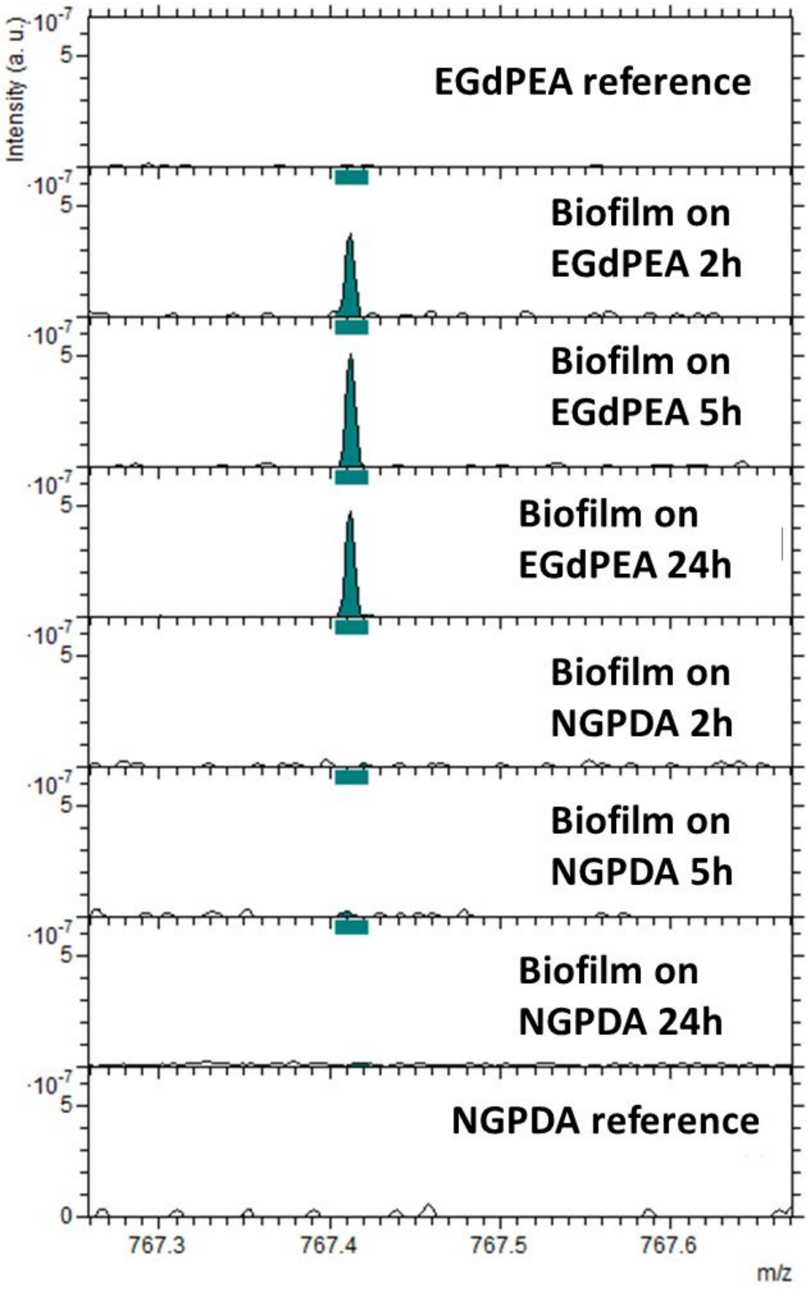

Supplementary Figure 10. Overlay of $C_{38}H_{65}O_{12}PNa^+$ (PI O-31:8), a lipid present in biofilm grown on EGdPEA and not on the biofilm grown on NGPDA.

Table S1. Saccharide and polysaccharide assignments made in the biofilm by setting the elemental limits and adding a restriction to H2n,On or H2n+1On, 5>n>15

m/z	Assignment	Description
293.1229	$C_{12}H_{21}O_8^+$	2 rhamnoses
309.1179	$C_{12}H_{21}O_9^+$	2 rhamnoses
315.1051	$C_{12}H_{20}NaO_8^+$	2 rhamnoses
149.0683	$C_5H_{11}NO_4^+$	glucosamine
150.0760	$C_5H_{12}NO_4^+$	glucosamine

164.0916	$C_6H_{14}NO_4^+$	glucosamine
172.0578	$C_5H_{11}NaNO_4^+$	glucosamine
180.0865	$C_6H_{14}NO_5^+$	glucosamine
220.1543	$C_{10}H_{22}NO_4^+$	glucosamine + C4H8
234.1699	$C_{11}H_{24}NO_4^+$	glucosamine + C5H10
248.1856	$C_{12}H_{26}NO_4^+$	glucosamine + C6H12
262.2013	$C_{13}H_{28}NO_4^+$	glucosamine + C7H14
276.2168	$C_{14}H_{30}NO_4^+$	glucosamine + C8H16
304.2480	$C_{16}H_{34}NO_4^+$	glucosamine + C9H18
228.0267	$C_5H_{11}NO_7P^+$	glucosamine phosphate
229.0346	$C_5H_{12}NO_7P^+$	glucosamine phosphate
230.0423	$C_5H_{13}NO_7P^+$	glucosamine phosphate
251.0165	$C_5H_{11}NaNO_7P^+$	glucosamine phosphate
252.0242	$C_5H_{12}NaNO_7P^+$	glucosamine phosphate
259.0453	$C_6H_{14}NO_8P^+$	glucosamine phosphate
192.1229	$C_8H_{18}NO_4^+$	glucosamine + C2H4
208.1179	$C_8H_{18}NO_5^+$	glucosamine + C2H4
206.1387	$C_9H_{20}NO_4^+$	glucosamine + C3H6
178.1072	$C_7H_{16}NO_4^+$	glucosamine + CH2
165.0757	$C_6H_{13}O_5^+$	glucose
180.0629	$C_6H_{12}O_6^+$	glucose
181.0706	$C_6H_{13}O_6^+$	glucose
187.0576	$C_6H_{12}O_5Na^+$	glucose
202.0447	$C_6H_{11}O_6Na^+$	glucose
203.0525	$C_6H_{12}O_6Na^+$	glucose
259.0214	$C_6H_{12}O_6PO_3^+$	glucose phosphate
281.0031	$C_6H_{11}O_6PO_3Na^+$	glucose phosphate
195.0500	$C_6H_{11}O_7^+$	glucuronic acid
217.0318	$C_6H_{10}O_7Na^+$	glucuronic acid
359.0610	$C_{10}H_{18}NO_{11}P^+$	lipid A
373.0762	$C_{11}H_{20}NO_{11}P^+$	lipid A
387.0918	$C_{12}H_{22}NO_{11}P^+$	lipid A
401.1075	$C_{13}H_{24}NO_{11}P^+$	lipid A
415.1229	$C_{14}H_{26}NO_{11}P^+$	lipid A
429.1385	$C_{15}H_{28}NO_{11}P^+$	lipid A
443.1542	$C_{16}H_{30}NO_{11}P^+$	lipid A
457.1699	$C_{17}H_{32}NO_{11}P^+$	lipid A
469.1699	$C_{18}H_{32}NO_{11}P^+$	lipid A
471.1857	$C_{18}H_{34}NO_{11}P^+$	lipid A
485.2010	$C_{19}H_{36}NO_{11}P^+$	lipid A
497.2010	$C_{20}H_{36}NO_{11}P^+$	lipid A
499.2168	$C_{20}H_{38}NO_{11}P^+$	lipid A
513.2320	$C_{21}H_{40}NO_{11}P^+$	lipid A
527.2478	$C_{22}H_{42}NO_{11}P^+$	lipid A
541.2640	$C_{23}H_{44}NO_{11}P^+$	lipid A
543.2797	$C_{23}H_{46}NO_{11}P^+$	lipid A
569.2953	$C_{25}H_{48}NO_{11}P^+$	lipid A
571.3110	$C_{25}H_{50}NO_{11}P^+$	lipid A

593.2933	$C_{25}H_{49}NO_{11}PNa^+$	lipid A
597.3267	$C_{27}H_{52}NO_{11}P^+$	lipid A
599.3422	$C_{27}H_{54}NO_{11}P^+$	lipid A
621.3246	$C_{27}H_{53}NO_{11}PNa^+$	lipid A
595.3107	$C_{27}H_{50}NO_{11}P^+$	llipid A
132.0417	$C_5H_8O_4^+$	polysaccharide
144.0418	$C_6H_8O_4^+$	polysaccharide
157.0495	$C_7H_9O_4^+$	polysaccharide
159.0650	$C_7H_{11}O_4^+$	polysaccharide
162.0522	$C_6H_{10}O_5^+$	polysaccharide
167.0313	$C_6H_8O_4Na^+$	polysaccharide
174.0524	$C_7H_{10}O_5^+$	polysaccharide
185.0419	$C_6H_{10}O_5Na^+$	polysaccharide
304.2998	$C_{21}H_{38}N^+$	polysaccharide
326.3781	$C_{22}H_{48}N^+$	polysaccharide
365.1054	$C_{12}H_{22}O_{11}Na^+$	polysaccharide
377.1060	$C_{13}H_{22}O_{11}Na^+$	polysaccharide
527.1586	$C_{18}H_{32}O_{16}Na^+$	polysaccharide
637.3784	$C_{31}H_{57}O_{13}^+$	rhamnoliipd
203.0164	$C_5H_8NaO_7^+$	rhamnolipid
217.0318	$C_6H_{10}NaO_7^+$	rhamnolipid
231.0476	$C_7H_{12}NaO_7^+$	rhamnolipid
245.0631	$C_8H_{14}NaO_7^+$	rhamnolipid
277.0529	$C_8H_{14}NaO_9^+$	rhamnolipid
303.0683	$C_{10}H_{16}NaO_9^+$	rhamnolipid
317.0841	$C_{11}H_{18}NaO_9^+$	rhamnolipid
327.1414	$C_{14}H_{24}NaO_7^+$	rhamnolipid
331.0999	$C_{12}H_{20}NaO_9^+$	rhamnolipid
341.1569	$C_{15}H_{26}NaO_7^+$	rhamnolipid
355.1725	$C_{16}H_{28}NaO_7^+$	rhamnolipid
359.1320	$C_{14}H_{24}NaO_9^+$	rhamnolipid
365.1811	$C_{16}H_{29}O_9^+$	rhamnolipid
369.1881	$C_{17}H_{30}NaO_7^+$	rhamnolipid
373.1477	$C_{15}H_{26}NaO_9^+$	rhamnolipid
379.1967	$C_{17}H_{31}O_9^+$	rhamnolipid
383.2037	$C_{18}H_{32}NaO_7^+$	rhamnolipid
387.1626	$C_{16}H_{28}NaO_9^+$	rhamnolipid
393.2122	$C_{18}H_{33}O_9^+$	rhamnolipid
401.1782	$C_{17}H_{30}NaO_9^+$	rhamnolipid
407.2271	$C_{19}H_{35}O_9^+$	rhamnolipid
415.1941	$C_{18}H_{32}NaO_9^+$	rhamnolipid
421.2434	$C_{20}H_{37}O_9^+$	rhamnolipid
429.2093	$C_{19}H_{34}NaO_9^+$	rhamnolipid
443.2249	$C_{20}H_{36}NaO_9^+$	rhamnolipid
449.2744	$C_{22}H_{41}O_9^+$	rhamnolipid
457.2404	$C_{21}H_{38}NaO_9^+$	rhamnolipid
471.2562	$C_{22}H_{40}NaO_9^+$	rhamnolipid

480.2561	$C_{22}H_{40}O_{11}^+$	rhamnolipid	3-O-(alpha-L-rhamnopyranosyl-(1-2)-alpha-L-rhamnopyranosyl)-3-hydroxydecanoic acid
485.2719	$C_{23}H_{42}NaO_9^+$	rhamnolipid	
494.2723	$C_{23}H_{42}O_{11}^+$	rhamnolipid	
499.2874	$C_{24}H_{44}NaO_9^+$	rhamnolipid	3-O-alpha-L-rhamnopyranosyl-3-hydroxynonanoyl-3-hydroxydecanoic acid
505.3372	$C_{26}H_{49}O_9^+$	rhamnolipid	3-O-alpha-L-rhamnopyranosyl-3-hydroxydecanoyl-3-hydroxydecanoic acid
508.2880	$C_{24}H_{44}O_{11}^+$	rhamnolipid	
513.3030	$C_{25}H_{46}NaO_9^+$	rhamnolipid	
519.3520	$C_{27}H_{51}O_9^+$	rhamnolipid	3-O-alpha-L-rhamnopyranosyl-3-hydroxyundecanoyl-3-hydroxydecanoic acid
522.3030	$C_{25}H_{46}O_{11}^+$	rhamnolipid	
525.2534	$C_{23}H_{41}O_{13}^+$	rhamnolipid	
527.3191	$C_{26}H_{48}NaO_9^+$	rhamnolipid	
533.3687	$C_{28}H_{53}O_9^+$	rhamnolipid	3-O-alpha-L-rhamnopyranosyl-3-hydroxyundecanoyl-3-hydroxydecanoic acid
539.2698	$C_{24}H_{43}O_{13}^+$	rhamnolipid	
541.3343	$C_{27}H_{50}NaO_9^+$	rhamnolipid	
553.2854	$C_{25}H_{45}O_{13}^+$	rhamnolipid	
567.3002	$C_{26}H_{47}O_{13}^+$	rhamnolipid	
568.3576	$C_{29}H_{53}NaO_9^+$	rhamnolipid	
577.2832	$C_{25}H_{46}NaO_{13}^+$	rhamnolipid	
581.3160	$C_{27}H_{49}O_{13}^+$	rhamnolipid	
582.3729	$C_{30}H_{55}NaO_9^+$	rhamnolipid	3-O-alpha-L-rhamnopyranosyl-3-hydroxyundecanoyl-3-hydroxydecanoic acid
595.3316	$C_{28}H_{51}O_{13}^+$	rhamnolipid	
609.3472	$C_{29}H_{53}O_{13}^+$	rhamnolipid	
623.3631	$C_{30}H_{55}O_{13}^+$	rhamnolipid	
651.3952	$C_{32}H_{59}O_{13}^+$	rhamnolipid	3-O-(alpha-L-rhamnopyranosyl-(1-2)-alpha-L-rhamnopyranosyl)-3-hydroxydecanoyl-3-hydroxydecanoic acid
665.4100	$C_{33}H_{61}O_{13}^+$	rhamnolipid	
673.3770	$C_{32}H_{58}NaO_{13}^+$	rhamnolipid	
679.4263	$C_{34}H_{63}O_{13}^+$	rhamnolipid	
687.3917	$C_{33}H_{60}NaO_{13}^+$	rhamnolipid	
701.4083	$C_{34}H_{62}NaO_{13}^+$	rhamnolipid	
729.4403	$C_{36}H_{66}NaO_{13}^+$	rhamnolipid	
164.0679	$C_6H_{12}O_5^+$	rhamnose	
186.0498	$C_6H_{11}NaO_5^+$	rhamnose	
242.0187	$C_6H_{11}O_5PO_3^+$	rhamnose phosphate	
244.0347	$C_6H_{13}O_5PO_3^+$	rhamnose phosphate	
264.0005	$C_6H_{10}O_5PO_3Na^+$	rhamnose phosphate	

Table S2. Lipids assigned and categorised by matching with the LipidMaps database, linked to the SIMS-MFP process.

	Identified lipid	Protonated $[M+H]^+$	Sodium adduct $[M+Na]^+$	Potassium adduct $[M+K]^+$
1	ACer 42:1;O6/HexCer 36:1;O2	-	-	$C_{42}H_{81}NO_8K^+$
2	ACer 42:2;O4/Cer 42:3;O5	-	-	$C_{42}H_{79}NO_6K^+$
3	ACer 42:2;O6/HexCer 36:2;O2	-	-	$C_{42}H_{79}NO_8K^+$
4	ACer 42:3;O4/Cer 42:4;O5	-	-	$C_{42}H_{77}NO_6K^+$
5	ACer 42:4;O4/Cer 42:5;O5	-	-	$C_{42}H_{75}NO_6K^+$

6	ACer 42:6;O3	-	$C_{42}H_{71}NO_5Na^+$	-
7	ACer 43:1;O6/HexCer 37:1;O2	-	-	$C_{43}H_{83}NO_8K^+$
8	ACer 43:2;O6/HexCer 37:2;O2	-	-	$C_{43}H_{81}NO_8K^+$
9	ACer 43:3;O4/Cer 43:4;O5	-	-	$C_{43}H_{79}NO_6K^+$
10	ACer 43:4;O4/Cer 43:5;O5	-	-	$C_{43}H_{77}NO_6K^+$
11	ACer 43:4;O6/HexCer 37:4;O2	-	-	$C_{43}H_{77}NO_8K^+$
12	ACer 44:2;O4/Cer 44:3;O5	-	-	$C_{44}H_{83}NO_6K^+$
13	ACer 45:6;O5	-	$C_{45}H_{77}NO_7Na^+$	-
14	ACer 46:6;O5	-	$C_{46}H_{79}NO_7Na^+$	-
15	ACer 54:2;O2/Cer 54:3;O3	-	$C_{54}H_{103}NO_4Na^+$	-
16	ACer 56:4;O3	$C_{56}H_{104}NO_5^+$	-	-
17	BMP 11:2;O	-	$C_{17}H_{29}O_{11}PNa^+$	-
18	BMP 12:1;O	-	$C_{18}H_{33}O_{11}PNa^+$	-
19	BMP 13:0;O/LPI O-10:1	-	$C_{19}H_{37}O_{11}PNa^+$	-
20	BMP 14:0;O/LPI O-11:1	-	$C_{20}H_{39}O_{11}PNa^+$	-
21	BMP 16:1/LPG 16:2;O	-	-	$C_{22}H_{41}O_{10}PK^+$
22	BMP 17:2;O/LPI O-14:3	-	-	$C_{23}H_{41}O_{11}PK^+$
23	BMP 18:0;O/LPI O-15:1	$C_{24}H_{48}O_{11}P^+$	-	-
24	BMP 18:1/LPG 18:2;O	-	$C_{24}H_{45}O_{10}PNa^+$	-
25	BMP 20:0;O/LPI O-17:1/PG 20:0;O	-	$C_{26}H_{51}O_{11}PNa^+$	-
26	BMP 22:0;O/LPI O-19:1/PG 22:0;O	-	$C_{28}H_{55}O_{11}PNa^+$	-
27	BMP 23:0;O/LPI O-20:1/PG 23:0;O	-	$C_{29}H_{57}O_{11}PNa^+$	-
28	BMP 23:1;O/LPI O-20:2/PG 23:1;O	-	-	$C_{29}H_{55}O_{11}PK^+$
29	BMP 26:1;O/LPI O-23:2/PG 26:1;O	-	$C_{32}H_{61}O_{11}PNa^+$	-
30	BMP 26:6/LPG 26:7;O/PG 26:6/PG O-26:7;O	-	$C_{32}H_{51}O_{10}PNa^+$	-
31	BMP 31:1/LPG 31:2;O/PG 31:1/PG O-31:2;O	-	$C_{37}H_{71}O_{10}PNa^+$	-
32	BMP 32:1;O/LPI O-29:2/PG 32:1;O	$C_{38}H_{74}O_{11}P^+$	-	-
33	BMP 33:1/LPG 33:2;O/PG 33:1/PG O-33:2;O	-	$C_{39}H_{75}O_{10}PNa^+$	-
34	BMP 33:2/LPG 33:3;O/PG 33:2/PG O-33:3;O	-	$C_{39}H_{73}O_{10}PNa^+$	-
35	BMP 33:4/LPG 33:5;O/PG 33:4/PG O-33:5;O	$C_{39}H_{70}O_{10}P^+$	-	-
36	BMP 34:1/LPG 34:2;O/PG 34:1/PG O-34:2;O	-	$C_{40}H_{77}O_{10}PNa^+$	$C_{40}H_{77}O_{10}PK^+$
37	BMP 34:2/LPG 34:3;O/PG 34:2/PG O-34:3;O	-	$C_{40}H_{75}O_{10}PNa^+$	-
38	CAR 10:0;O/NAE 15:1;O3	$C_{17}H_{34}NO_5^+$	$C_{17}H_{33}NO_5Na^+$	-
39	CAR 10:0;O2/NAE 15:1;O4	-	$C_{17}H_{33}NO_6Na^+$	-
40	CAR 10:0;O3	$C_{17}H_{34}NO_7^+$	$C_{17}H_{33}NO_7Na^+$	-
41	CAR 10:0;O4	-	$C_{17}H_{33}NO_8Na^+$	-
42	CAR 10:1;O/NAE 15:2;O3	-	$C_{17}H_{31}NO_5Na^+$	-
43	CAR 10:1;O2/NAE 15:2;O4	-	$C_{17}H_{31}NO_6Na^+$	-
44	CAR 10:1;O3	-	$C_{17}H_{31}NO_7Na^+$	-
45	CAR 10:1;O4	$C_{17}H_{32}NO_8^+$	$C_{17}H_{31}NO_8Na^+$	-
46	CAR 10:2;O2/NAE 15:3;O4	-	$C_{17}H_{29}NO_6Na^+$	-
47	CAR 10:2;O3	-	$C_{17}H_{29}NO_7Na^+$	-
48	CAR 10:2;O4	-	$C_{17}H_{29}NO_8Na^+$	-
49	CAR 11:0;O/NAE 16:1;O3	-	$C_{18}H_{35}NO_5Na^+$	-
50	CAR 11:0;O2/NAE 16:1;O4	-	$C_{18}H_{35}NO_6Na^+$	-
51	CAR 11:0;O3	$C_{18}H_{36}NO_7^+$	$C_{18}H_{35}NO_7Na^+$	-
52	CAR 11:0;O4	$C_{18}H_{36}NO_8^+$	$C_{18}H_{35}NO_8Na^+$	-
53	CAR 11:1;O/NAE 16:2;O3	-	$C_{18}H_{33}NO_5Na^+$	-

54	CAR 11:1;O2/NAE 16:2;O4	-	$C_{18}H_{33}NO_6Na^+$	-
55	CAR 11:1;O3	-	$C_{18}H_{33}NO_7Na^+$	-
56	CAR 11:1;O4	$C_{18}H_{34}NO_8^+$	$C_{18}H_{33}NO_8Na^+$	-
57	CAR 11:2;O/NAE 16:3;O3	-	$C_{18}H_{31}NO_5Na^+$	-
58	CAR 11:2;O2/NAE 16:3;O4	-	$C_{18}H_{31}NO_6Na^+$	-
59	CAR 11:2;O3	-	$C_{18}H_{31}NO_7Na^+$	-
60	CAR 11:2;O4	-	$C_{18}H_{31}NO_8Na^+$	-
61	CAR 11:3;O2/NAE 16:4;O4	-	$C_{18}H_{29}NO_6Na^+$	-
62	CAR 11:3;O3	-	$C_{18}H_{29}NO_7Na^+$	-
63	CAR 11:3;O4	-	$C_{18}H_{29}NO_8Na^+$	-
64	CAR 12:0;O/NAE 17:1;O3	-	$C_{19}H_{37}NO_5Na^+$	-
65	CAR 12:0;O2/NAE 17:1;O4	-	$C_{19}H_{37}NO_6Na^+$	-
66	CAR 12:0;O3	-	$C_{19}H_{37}NO_7Na^+$	-
67	CAR 12:0;O4	-	$C_{19}H_{37}NO_8Na^+$	-
68	CAR 12:1;O/NAE 17:2;O3	-	$C_{19}H_{35}NO_5Na^+$	-
69	CAR 12:1;O2/NAE 17:2;O4	-	$C_{19}H_{35}NO_6Na^+$	-
70	CAR 12:1;O3	-	$C_{19}H_{35}NO_7Na^+$	-
71	CAR 12:1;O4	$C_{19}H_{36}NO_8^+$	$C_{19}H_{35}NO_8Na^+$	-
72	CAR 12:2;O/NAE 17:3;O3	-	$C_{19}H_{33}NO_5Na^+$	-
73	CAR 12:2;O2/NAE 17:3;O4	-	$C_{19}H_{33}NO_6Na^+$	-
74	CAR 12:2;O3	-	$C_{19}H_{33}NO_7Na^+$	-
75	CAR 12:2;O4	-	$C_{19}H_{33}NO_8Na^+$	-
76	CAR 12:3;O2/NAE 17:4;O4	-	$C_{19}H_{31}NO_6Na^+$	-
77	CAR 12:3;O3	-	$C_{19}H_{31}NO_7Na^+$	-
78	CAR 12:3;O4	-	$C_{19}H_{31}NO_8Na^+$	-
79	CAR 13:0;O/NAE 18:1;O3	$C_{20}H_{40}NO_5^+$	$C_{20}H_{39}NO_5Na^+$	-
80	CAR 13:0;O2/NAE 18:1;O4	-	$C_{20}H_{39}NO_6Na^+$	-
81	CAR 13:0;O3	-	$C_{20}H_{39}NO_7Na^+$	-
82	CAR 13:0;O4	-	$C_{20}H_{39}NO_8Na^+$	-
83	CAR 13:1;O/NAE 18:2;O3	-	$C_{20}H_{37}NO_5Na^+$	-
84	CAR 13:1;O2/NAE 18:2;O4	-	$C_{20}H_{37}NO_6Na^+$	-
85	CAR 13:1;O3	-	$C_{20}H_{37}NO_7Na^+$	-
86	CAR 13:1;O4	$C_{20}H_{38}NO_8^+$	$C_{20}H_{37}NO_8Na^+$	-
87	CAR 13:2;O/NAE 18:3;O3	-	$C_{20}H_{35}NO_5Na^+$	-
88	CAR 13:2;O2/NAE 18:3;O4	-	$C_{20}H_{35}NO_6Na^+$	$C_{20}H_{35}NO_6K^+$
89	CAR 13:2;O3	-	$C_{20}H_{35}NO_7Na^+$	-
90	CAR 13:2;O4	-	$C_{20}H_{35}NO_8Na^+$	-
91	CAR 13:3;O2/NAE 18:4;O4/ST 18:0;O5;G	-	$C_{20}H_{33}NO_6Na^+$	-
92	CAR 13:3;O3/ST 18:0;O6;G	-	$C_{20}H_{33}NO_7Na^+$	-
93	CAR 13:3;O4/ST 18:0;O7;G	-	$C_{20}H_{33}NO_8Na^+$	-
94	CAR 14:0;O/NAE 19:1;O3	-	$C_{21}H_{41}NO_5Na^+$	-
95	CAR 14:0;O2/NAE 19:1;O4	-	$C_{21}H_{41}NO_6Na^+$	-
96	CAR 14:0;O3	-	$C_{21}H_{41}NO_7Na^+$	-
97	CAR 14:0;O4	-	$C_{21}H_{41}NO_8Na^+$	$C_{21}H_{41}NO_8K^+$
98	CAR 14:1;O/NAE 19:2;O3	-	$C_{21}H_{39}NO_5Na^+$	-
99	CAR 14:1;O2/NAE 19:2;O4	-	$C_{21}H_{39}NO_6Na^+$	-
100	CAR 14:1;O3	-	$C_{21}H_{39}NO_7Na^+$	-
101	CAR 14:1;O4	-	$C_{21}H_{39}NO_8Na^+$	-

102	CAR 14:2;O/NAE 19:3;O3	-	$C_{21}H_{37}NO_5Na^+$	-
103	CAR 14:2;O2/NAE 19:3;O4	-	$C_{21}H_{37}NO_6Na^+$	-
104	CAR 14:2;O3	-	$C_{21}H_{37}NO_7Na^+$	-
105	CAR 14:2;O4	-	$C_{21}H_{37}NO_8Na^+$	-
106	CAR 14:3;O2/NAE 19:4;O4/ST 19:0;O5;G	-	$C_{21}H_{35}NO_6Na^+$	-
107	CAR 14:3;O3/ST 19:0;O6;G	-	$C_{21}H_{35}NO_7Na^+$	-
108	CAR 14:3;O4/ST 19:0;O7;G	-	$C_{21}H_{35}NO_8Na^+$	-
109	CAR 14:4;O3/ST 19:1;O6;G	-	$C_{21}H_{33}NO_7Na^+$	-
110	CAR 14:4;O4/ST 19:1;O7;G	-	$C_{21}H_{33}NO_8Na^+$	-
111	CAR 15:0;O/NAE 20:1;O3	$C_{22}H_{44}NO_5^+$	$C_{22}H_{43}NO_5Na^+$	-
112	CAR 15:0;O2/NAE 20:1;O4	-	$C_{22}H_{43}NO_6Na^+$	-
113	CAR 15:0;O3	-	$C_{22}H_{43}NO_7Na^+$	-
114	CAR 15:0;O4	-	$C_{22}H_{43}NO_8Na^+$	-
115	CAR 15:1;O2/NAE 20:2;O4	-	$C_{22}H_{41}NO_6Na^+$	-
116	CAR 15:1;O3	-	$C_{22}H_{41}NO_7Na^+$	-
117	CAR 15:1;O4	-	$C_{22}H_{41}NO_8Na^+$	-
118	CAR 15:2;O2/NAE 20:3;O4	-	$C_{22}H_{39}NO_6Na^+$	-
119	CAR 15:2;O3	-	$C_{22}H_{39}NO_7Na^+$	-
120	CAR 15:2;O4	-	$C_{22}H_{39}NO_8Na^+$	-
121	CAR 15:3;O2/NAE 20:4;O4/ST 20:0;O5;G	-	$C_{22}H_{37}NO_6Na^+$	-
122	CAR 15:3;O3/ST 20:0;O6;G	-	$C_{22}H_{37}NO_7Na^+$	-
123	CAR 15:3;O4/ST 20:0;O7;G	-	$C_{22}H_{37}NO_8Na^+$	-
124	CAR 15:4;O2/NAE 20:5;O4/ST 20:1;O5;G	-	$C_{22}H_{35}NO_6Na^+$	-
125	CAR 15:4;O3/ST 20:1;O6;G	-	$C_{22}H_{35}NO_7Na^+$	-
126	CAR 15:4;O4/ST 20:1;O7;G	-	$C_{22}H_{35}NO_8Na^+$	-
127	CAR 16:0;O/NAE 21:1;O3	-	-	$C_{23}H_{45}NO_5K^+$
128	CAR 16:0;O2/NAE 21:1;O4	-	$C_{23}H_{45}NO_6Na^+$	-
129	CAR 16:0;O3	-	$C_{23}H_{45}NO_7Na^+$	-
130	CAR 16:0;O4	-	$C_{23}H_{45}NO_8Na^+$	-
131	CAR 16:1/NAE 21:2;O2	-	-	$C_{23}H_{43}NO_4K^+$
132	CAR 16:1;O/NAE 21:2;O3	-	-	$C_{23}H_{43}NO_5K^+$
133	CAR 16:1;O2/NAE 21:2;O4	-	$C_{23}H_{43}NO_6Na^+$	-
134	CAR 16:1;O3	-	$C_{23}H_{43}NO_7Na^+$	-
135	CAR 16:1;O4	-	$C_{23}H_{43}NO_8Na^+$	-
136	CAR 16:2/NAE 21:3;O2	-	-	$C_{23}H_{41}NO_4K^+$
137	CAR 16:2;O2/NAE 21:3;O4	-	$C_{23}H_{41}NO_6Na^+$	-
138	CAR 16:2;O3	-	$C_{23}H_{41}NO_7Na^+$	-
139	CAR 16:2;O4	-	$C_{23}H_{41}NO_8Na^+$	-
140	CAR 16:3;O2/NAE 21:4;O4/ST 21:0;O5;G	-	$C_{23}H_{39}NO_6Na^+$	-
141	CAR 16:3;O3/ST 21:0;O6;G	-	$C_{23}H_{39}NO_7Na^+$	-
142	CAR 16:3;O4/ST 21:0;O7;G	-	$C_{23}H_{39}NO_8Na^+$	-
143	CAR 16:4;O3/ST 21:1;O6;G	-	$C_{23}H_{37}NO_7Na^+$	-
144	CAR 16:4;O4/ST 21:1;O7;G	-	$C_{23}H_{37}NO_8Na^+$	-
145	CAR 17:0;O3	-	$C_{24}H_{47}NO_7Na^+$	-
146	CAR 17:0;O4	-	$C_{24}H_{47}NO_8Na^+$	-
147	CAR 17:1;O2/NAE 22:2;O4	-	$C_{24}H_{45}NO_6Na^+$	-
148	CAR 17:1;O3	-	$C_{24}H_{45}NO_7Na^+$	-
149	CAR 17:1;O4	-	$C_{24}H_{45}NO_8Na^+$	-

150	CAR 17:2;O2/NAE 22:3;O4	-	$C_{24}H_{43}NO_6Na^+$	-
151	CAR 17:2;O3	-	$C_{24}H_{43}NO_7Na^+$	-
152	CAR 17:2;O4	-	$C_{24}H_{43}NO_8Na^+$	-
153	CAR 17:3/NAE 22:4;O2/ST 22:0;O3;G	$C_{24}H_{42}NO_4^+$	-	-
154	CAR 17:3;O2/NAE 22:4;O4/ST 22:0;O5;G	-	$C_{24}H_{41}NO_6Na^+$	-
155	CAR 17:3;O3/ST 22:0;O6;G	-	$C_{24}H_{41}NO_7Na^+$	-
156	CAR 17:3;O4/ST 22:0;O7;G	-	$C_{24}H_{41}NO_8Na^+$	-
157	CAR 17:4;O3/ST 22:1;O6;G	-	$C_{24}H_{39}NO_7Na^+$	-
158	CAR 17:4;O4/ST 22:1;O7;G	-	$C_{24}H_{39}NO_8Na^+$	-
159	CAR 18:0;O3	-	$C_{25}H_{49}NO_7Na^+$	-
160	CAR 18:0;O4	-	$C_{25}H_{49}NO_8Na^+$	-
161	CAR 18:1;O/NAE 23:2;O3	-	-	$C_{25}H_{47}NO_5K^+$
162	CAR 18:1;O3	-	$C_{25}H_{47}NO_7Na^+$	-
163	CAR 18:1;O4	-	$C_{25}H_{47}NO_8Na^+$	-
164	CAR 18:2/NAE 23:3;O2	-	-	$C_{25}H_{45}NO_4K^+$
165	CAR 18:2;O/NAE 23:3;O3	-	-	$C_{25}H_{45}NO_5K^+$
166	CAR 18:2;O2/NAE 23:3;O4	-	$C_{25}H_{45}NO_6Na^+$	$C_{25}H_{45}NO_6K^+$
167	CAR 18:2;O3	-	$C_{25}H_{45}NO_7Na^+$	-
168	CAR 18:2;O4	-	$C_{25}H_{45}NO_8Na^+$	-
169	CAR 18:3;O2/NAE 23:4;O4/ST 23:0;O5;G	-	$C_{25}H_{43}NO_6Na^+$	-
170	CAR 18:3;O3/ST 23:0;O6;G	-	$C_{25}H_{43}NO_7Na^+$	-
171	CAR 18:3;O4/ST 23:0;O7;G	-	$C_{25}H_{43}NO_8Na^+$	-
172	CAR 18:4;O2/NAE 23:5;O4/ST 23:1;O5;G	-	$C_{25}H_{41}NO_6Na^+$	-
173	CAR 18:4;O3/ST 23:1;O6;G	-	$C_{25}H_{41}NO_7Na^+$	-
174	CAR 18:4;O4/ST 23:1;O7;G	-	$C_{25}H_{41}NO_8Na^+$	-
175	CAR 18:5;O4/ST 23:2;O7;G	-	$C_{25}H_{39}NO_8Na^+$	-
176	CAR 19:0;O4	-	$C_{26}H_{51}NO_8Na^+$	-
177	CAR 19:1;O/NAE 24:2;O3	-	-	$C_{26}H_{49}NO_5K^+$
178	CAR 19:1;O2/NAE 24:2;O4	-	-	$C_{26}H_{49}NO_6K^+$
179	CAR 19:1;O3	-	$C_{26}H_{49}NO_7Na^+$	-
180	CAR 19:1;O4	-	$C_{26}H_{49}NO_8Na^+$	-
181	CAR 19:2;O2/NAE 24:3;O4	-	-	$C_{26}H_{47}NO_6K^+$
182	CAR 19:2;O4	-	$C_{26}H_{47}NO_8Na^+$	-
183	CAR 19:3;O3/ST 24:0;O6;G	-	$C_{26}H_{45}NO_7Na^+$	-
184	CAR 19:3;O4/ST 24:0;O7;G	-	$C_{26}H_{45}NO_8Na^+$	-
185	CAR 19:4;O3/ST 18:0;O2;HexNAC/ST 24:1;O6;G	-	$C_{26}H_{43}NO_7Na^+$	-
186	CAR 19:4;O4/ST 18:0;O3;HexNAC/ST 24:1;O7;G	-	$C_{26}H_{43}NO_8Na^+$	-
187	CAR 19:5/NAE 24:6;O2/ST 24:2;O3;G	-	$C_{26}H_{41}NO_4Na^+$	-
188	CAR 19:5;O4/ST 18:1;O3;HexNAC/ST 24:2;O7;G	-	$C_{26}H_{41}NO_8Na^+$	-
189	CAR 20:1;O4	-	$C_{27}H_{51}NO_8Na^+$	-
190	CAR 20:2;O2/NAE 25:3;O4	-	-	$C_{27}H_{49}NO_6K^+$
191	CAR 20:2;O4	-	$C_{27}H_{49}NO_8Na^+$	-
192	CAR 20:3;O2/NAE 25:4;O4/ST 25:0;O5;G	-	-	$C_{27}H_{47}NO_6K^+$
193	CAR 20:3;O4/ST 25:0;O7;G	-	$C_{27}H_{47}NO_8Na^+$	-
194	CAR 20:4;O3/ST 19:0;O2;HexNAC/ST 25:1;O6;G	-	$C_{27}H_{45}NO_7Na^+$	-
195	CAR 20:4;O4/ST 19:0;O3;HexNAC/ST 25:1;O7;G	-	$C_{27}H_{45}NO_8Na^+$	-
196	CAR 21:2;O2/Cer 28:3;O5/NAE 26:3;O4	-	-	$C_{28}H_{51}NO_6K^+$

197	CAR 21:3;O2/Cer 28:4;O5/NAE 26:4;O4/ST 26:0;O5;G	-	-	$C_{28}H_{49}NO_6K^+$
198	CAR 21:3;O4/ST 26:0;O7;G	-	$C_{28}H_{49}NO_8Na^+$	-
199	CAR 21:6/NAE 26:7;O2/ST 26:3;O3;G	-	$C_{28}H_{43}NO_4Na^+$	-
200	CAR 22:2;O2/Cer 29:3;O5/NAE 27:3;O4	-	-	$C_{29}H_{53}NO_6K^+$
201	CAR 22:2;O4	-	$C_{29}H_{53}NO_8Na^+$	-
202	CAR 22:4;O2/Cer 29:5;O5/NAE 27:5;O4/ST 21:0;O;HexNAC/ST 27:1;O5;G	-	$C_{29}H_{49}NO_6Na^+$	-
203	CAR 22:4;O3/Cer 29:5;O6/ST 21:0;O2;HexNAC/ST 27:1;O6;G	-	$C_{29}H_{49}NO_7Na^+$	-
204	CAR 22:6/NAE 27:7;O2/ST 27:3;O3;G	-	$C_{29}H_{45}NO_4Na^+$	-
205	CAR 22:6;O/NAE 27:7;O3/ST 27:3;O4;G	-	$C_{29}H_{45}NO_5Na^+$	-
206	CAR 23:2;O2/Cer 30:3;O5/NAE 28:3;O4	-	-	$C_{30}H_{55}NO_6K^+$
207	CAR 23:2;O4	-	$C_{30}H_{55}NO_8Na^+$	-
208	CAR 23:3;O2/Cer 30:4;O5/NAE 28:4;O4/ST 28:0;O5;G	-	-	$C_{30}H_{53}NO_6K^+$
209	CAR 23:4;O3/Cer 30:5;O6/ST 22:0;O2;HexNAC/ST 28:1;O6;G	$C_{30}H_{52}NO_7^+$	-	-
210	CAR 24:2;O2/Cer 31:3;O5/NAE 29:3;O4	-	-	$C_{31}H_{57}NO_6K^+$
211	CAR 24:3;O2/Cer 31:4;O5/NAE 29:4;O4/ST 29:0;O5;G	-	-	$C_{31}H_{55}NO_6K^+$
212	CAR 25:2;O2/Cer 32:3;O5/NAE 30:3;O4	-	-	$C_{32}H_{59}NO_6K^+$
213	CAR 25:3;O2/Cer 32:4;O5/NAE 30:4;O4/ST 30:0;O5;G	-	-	$C_{32}H_{57}NO_6K^+$
214	CAR 25:7;O/ST 30:4;O4;G	-	$C_{32}H_{49}NO_5Na^+$	-
215	CAR 26:1;O2/Cer 33:2;O5/NAE 31:2;O4	-	-	$C_{33}H_{63}NO_6K^+$
216	CAR 26:2;O2/Cer 33:3;O5/NAE 31:3;O4	-	-	$C_{33}H_{61}NO_6K^+$
217	CAR 26:3;O2/Cer 33:4;O5/NAE 31:4;O4	-	-	$C_{33}H_{59}NO_6K^+$
218	CAR 26:7;O	-	$C_{33}H_{51}NO_5Na^+$	-
219	CAR 27:3;O2/Cer 34:4;O5/NAE 32:4;O4	-	-	$C_{34}H_{61}NO_6K^+$
220	CAR 27:7;O	-	$C_{34}H_{53}NO_5Na^+$	-
221	CAR 28:3;O2/Cer 35:4;O5/NAE 33:4;O4	-	-	$C_{35}H_{63}NO_6K^+$
222	CAR 28:4;O2/Cer 35:5;O5/NAE 33:5;O4/ST 27:0;O;HexNAC	-	-	$C_{35}H_{61}NO_6K^+$
223	CAR 28:7;O	-	$C_{35}H_{55}NO_5Na^+$	-
224	CAR 29:2;O2/Cer 36:3;O5/NAE 34:3;O4	-	-	$C_{36}H_{67}NO_6K^+$
225	CAR 29:3;O2/Cer 36:4;O5/NAE 34:4;O4	-	-	$C_{36}H_{65}NO_6K^+$
226	CAR 29:6;O/NAE 34:7;O3	-	$C_{36}H_{59}NO_5Na^+$	-
227	CAR 29:6;O2/NAE 34:7;O4/ST 28:2;O;HexNAC	-	-	$C_{36}H_{59}NO_6K^+$
228	CAR 29:7;O	-	$C_{36}H_{57}NO_5Na^+$	-
229	CAR 30:1;O/Cer 37:2;O4	$C_{37}H_{72}NO_5^+$	-	-
230	CAR 30:2;O2/Cer 37:3;O5	-	-	$C_{37}H_{69}NO_6K^+$
231	CAR 30:3;O2/Cer 37:4;O5	-	-	$C_{37}H_{67}NO_6K^+$
232	CAR 31:2;O2/Cer 38:3;O5	-	-	$C_{38}H_{71}NO_6K^+$
233	CAR 31:3;O2/Cer 38:4;O5	-	-	$C_{38}H_{69}NO_6K^+$
234	CAR 32:1;O2/Cer 39:2;O5	-	-	$C_{39}H_{75}NO_6K^+$
235	CAR 32:2;O2/Cer 39:3;O5	-	-	$C_{39}H_{73}NO_6K^+$
236	CAR 32:3;O2/Cer 39:4;O5	-	-	$C_{39}H_{71}NO_6K^+$
237	CAR 32:4;O2/Cer 39:5;O5	-	-	$C_{39}H_{69}NO_6K^+$
238	CAR 32:6;O4/HexCer 33:6;O2	-	$C_{39}H_{65}NO_8Na^+$	-
239	CAR 32:7;O	-	$C_{39}H_{63}NO_5Na^+$	-
240	CAR 32:7;O2	-	-	$C_{39}H_{63}NO_6K^+$
241	CAR 33:2;O2/Cer 40:3;O5	-	-	$C_{40}H_{75}NO_6K^+$

242	CAR 33:3;O2/Cer 40:4;O5	-	-	$C_{40}H_{73}NO_6K^+$
243	CAR 33:4;O/Cer 40:5;O4	$C_{40}H_{72}NO_5^+$	-	-
244	CAR 33:7;O	-	$C_{40}H_{65}NO_5Na^+$	-
245	CAR 34:2;O2/Cer 41:3;O5	-	-	$C_{41}H_{77}NO_6K^+$
246	CAR 34:2;O3/Cer 41:3;O6	-	-	$C_{41}H_{77}NO_7K^+$
247	CAR 34:3;O2/Cer 41:4;O5	-	-	$C_{41}H_{75}NO_6K^+$
248	CAR 34:3;O3/Cer 41:4;O6	-	-	$C_{41}H_{75}NO_7K^+$
249	CAR 34:4;O2/Cer 41:5;O5	-	-	$C_{41}H_{73}NO_6K^+$
250	CAR 34:7;O	-	$C_{41}H_{67}NO_5Na^+$	-
251	CE 10:0;O2/DG O-34:5/MG 34:5/MG O-34:6;O	$C_{37}H_{65}O_4^+$	-	-
252	CE 15:0;O2/DG O-39:5	$C_{42}H_{75}O_4^+$	-	-
253	CE 20:5;O2/DG O-44:10	-	-	$C_{47}H_{74}O_4K^+$
254	CE 21:6;O2/DG O-45:11	-	-	$C_{48}H_{74}O_4K^+$
255	CE 22:6;O2/DG O-46:11	-	-	$C_{49}H_{76}O_4K^+$
256	CE 27:5	-	-	$C_{54}H_{88}O_2K^+$
257	Cer 51:0;O2	-	-	$C_{51}H_{103}NO_3K^+$
258	Cer 51:1;O	-	-	$C_{51}H_{101}NO_2K^+$
259	Cer 53:4;O	-	-	$C_{53}H_{99}NO_2K^+$
260	Cer 54:4;O2	-	$C_{54}H_{101}NO_3Na^+$	-
261	Cer 54:5;O	-	$C_{54}H_{99}NO_2Na^+$	-
262	CerP 28:2;O4/LPC 20:2;O/LPE 23:2;O/LPS O-22:2/PC 20:1/PC O-20:2;O/PE 23:1/PE O-23:2;O	$C_{28}H_{55}NO_8P^+$	-	-
263	CerP 28:3;O4/LPC 20:3;O/LPE 23:3;O/LPS O-22:3/PC 20:2/PC O-20:3;O/PE 23:2/PE O-23:3;O	$C_{28}H_{53}NO_8P^+$	-	-
264	CerP 29:2;O4/LPC 21:2;O/LPE 24:2;O/LPS O-23:2/PC 21:1/PC O-21:2;O/PE 24:1/PE O-24:2;O	$C_{29}H_{57}NO_8P^+$	-	-
265	CerP 29:3;O4/LPC 21:3;O/LPE 24:3;O/LPS O-23:3/PC 21:2/PC O-21:3;O/PE 24:2/PE O-24:3;O	$C_{29}H_{55}NO_8P^+$	-	-
266	CerP 30:0;O6	-	$C_{30}H_{62}NO_{10}PNa^+$	-
267	CerP 30:2;O4/LPC 22:2;O/LPE 25:2;O/LPS O-24:2/PC 22:1/PC O-22:2;O/PE 25:1/PE O-25:2;O	$C_{30}H_{59}NO_8P^+$	-	-
268	CerP 30:3;O4/LPC 22:3;O/LPE 25:3;O/LPS O-24:3/PC 22:2/PC O-22:3;O/PE 25:2/PE O-25:3;O	$C_{30}H_{57}NO_8P^+$	-	-
269	CerP 31:1;O4/LPC 23:1;O/LPE 26:1;O/LPS O-25:1/PC 23:0/PC O-23:1;O/PE 26:0/PE O-26:1;O	$C_{31}H_{63}NO_8P^+$	-	-
270	CerP 31:2;O4/LPC 23:2;O/LPE 26:2;O/LPS O-25:2/PC 23:1/PC O-23:2;O/PE 26:1/PE O-26:2;O	$C_{31}H_{61}NO_8P^+$	-	-
271	CerP 31:3;O4/LPC 23:3;O/LPE 26:3;O/LPS O-25:3/PC 23:2/PC O-23:3;O/PE 26:2/PE O-26:3;O	$C_{31}H_{59}NO_8P^+$	-	-
272	CerP 32:2;O5/LPS 26:1/LPS O-26:2;O/PC 24:1;O/PE 27:1;O/PS O-26:1	-	-	$C_{32}H_{62}NO_9PK^+$
273	CerP 32:3;O4/LPC 24:3;O/LPE 27:3;O/LPS O-26:3/PC 24:2/PC O-24:3;O/PE 27:2/PE O-27:3;O	$C_{32}H_{61}NO_8P^+$	-	-
274	CerP 33:2;O4/LPC 25:2;O/LPE 28:2;O/LPS O-27:2/PC 25:1/PC O-25:2;O/PE 28:1/PE O-28:2;O	-	$C_{33}H_{64}NO_8PNa^+$	-
275	CerP 33:3;O4/LPC 25:3;O/LPE 28:3;O/LPS O-27:3/PC 25:2/PC O-25:3;O/PE 28:2/PE O-28:3;O	$C_{33}H_{63}NO_8P^+$	-	-
276	CerP 33:4;O4/LPC 25:4;O/LPE 28:4;O/LPS O-27:4/PC 25:3/PC O-25:4;O/PE 28:3/PE O-28:4;O	$C_{33}H_{61}NO_8P^+$	-	-
277	CerP 34:2;O4/LPC 26:2;O/LPE 29:2;O/LPS O-28:2/PC 26:1/PC O-26:2;O/PE 29:1/PE O-29:2;O	$C_{34}H_{67}NO_8P^+$	-	-
278	CerP 34:2;O5/LPS 28:1/LPS O-28:2;O/PC 26:1;O/PE 29:1;O/PS O-28:1	-	-	$C_{34}H_{66}NO_9PK^+$
279	CerP 34:3;O4/LPC 26:3;O/LPE 29:3;O/LPS O-28:3/PC 26:2/PC O-26:3;O/PE 29:2/PE O-29:3;O	$C_{34}H_{65}NO_8P^+$	-	-
280	CerP 34:3;O5/LPS 28:2/LPS O-28:3;O/PC 26:2;O/PE 29:2;O/PS O-28:2	-	-	$C_{34}H_{64}NO_9PK^+$
281	CerP 34:6;O4/LPC 26:6;O/LPE 29:6;O/LPS O-28:6/PC 26:5/PC O-26:6;O/PE 29:5/PE O-29:6;O	$C_{34}H_{59}NO_8P^+$	-	-
282	CerP 35:1;O4/LPC 27:1;O/LPE 30:1;O/LPS O-29:1/PC 27:0/PC O-27:1;O/PE 30:0/PE O-30:1;O	-	$C_{35}H_{70}NO_8PNa^+$	-
283	CerP 35:2;O4/LPC 27:2;O/LPE 30:2;O/LPS O-29:2/PC 27:1/PC O-27:2;O/PE 30:1/PE O-30:2;O	$C_{35}H_{69}NO_8P^+$	-	-

284	CerP 35:3;O4/LPC 27:3;O/LPE 30:3;O/LPS O-29:3/PC 27:2/PC O-27:3;O/PE 30:2/PE O-30:3;O	$C_{35}H_{67}NO_8P^+$	-	-
285	CerP 36:0;O4/LPC 28:0;O/LPE 31:0;O/LPS O-30:0/PC O-28:0;O/PE O-31:0;O	-	$C_{36}H_{74}NO_8PNa^+$	-
286	CerP 36:2;O4/LPC 28:2;O/LPE 31:2;O/LPS O-30:2/PC 28:1/PC O-28:2;O/PE 31:1/PE O-31:2;O	$C_{36}H_{71}NO_8P^+$	-	-
287	CerP 36:3;O4/LPC 28:3;O/LPE 31:3;O/LPS O-30:3/PC 28:2/PC O-28:3;O/PE 31:2/PE O-31:3;O	$C_{36}H_{69}NO_8P^+$	-	-
288	CerP 37:1;O4/LPC 29:1;O/LPE 32:1;O/LPS O-31:1/PC 29:0/PC O-29:1;O/PE 32:0/PE O-32:1;O	$C_{37}H_{75}NO_8P^+$	-	-
289	CerP 37:2;O4/LPC 29:2;O/LPE 32:2;O/LPS O-31:2/PC 29:1/PC O-29:2;O/PE 32:1/PE O-32:2;O	$C_{37}H_{73}NO_8P^+$	$C_{37}H_{72}NO_8PNa^+$	$C_{37}H_{72}NO_8PK^+$
290	CerP 37:3;O4/LPC 29:3;O/LPE 32:3;O/LPS O-31:3/PC 29:2/PC O-29:3;O/PE 32:2/PE O-32:3;O	$C_{37}H_{71}NO_8P^+$	-	$C_{37}H_{70}NO_8PK^+$
291	CerP 37:4;O4/LPC 29:4;O/LPE 32:4;O/LPS O-31:4/PC 29:3/PC O-29:4;O/PE 32:3/PE O-32:4;O	$C_{37}H_{69}NO_8P^+$	-	-
292	CerP 38:2;O4/LPC 30:2;O/LPE 33:2;O/LPS O-32:2/PC 30:1/PC O-30:2;O/PE 33:1/PE O-33:2;O	$C_{38}H_{75}NO_8P^+$	$C_{38}H_{74}NO_8PNa^+$	-
293	CerP 38:3;O4/LPC 30:3;O/LPE 33:3;O/LPS O-32:3/PC 30:2/PC O-30:3;O/PE 33:2/PE O-33:3;O	$C_{38}H_{73}NO_8P^+$	-	-
294	CerP 39:1;O6/LPS 33:0;O/PS O-33:0;O	-	$C_{39}H_{78}NO_{10}PNa^+$	-
295	CerP 39:2;O4/LPC 31:2;O/LPE 34:2;O/LPS O-33:2/PC 31:1/PC O-31:2;O/PE 34:1/PE O-34:2;O	$C_{39}H_{77}NO_8P^+$	$C_{39}H_{76}NO_8PNa^+$	$C_{39}H_{76}NO_8PK^+$
296	CerP 39:2;O5/LPS 33:1/LPS O-33:2;O/PC 31:1;O/PE 34:1;O/PS O-33:1	$C_{39}H_{77}NO_9P^+$	-	-
297	CerP 39:3;O4/LPC 31:3;O/LPE 34:3;O/LPS O-33:3/PC 31:2/PC O-31:3;O/PE 34:2/PE O-34:3;O	$C_{39}H_{75}NO_8P^+$	$C_{39}H_{74}NO_8PNa^+$	$C_{39}H_{74}NO_8PK^+$
298	CerP 39:3;O5/LPS 33:2/LPS O-33:3;O/PC 31:2;O/PE 34:2;O/PS O-33:2	$C_{39}H_{75}NO_9P^+$	-	-
299	CerP 39:4;O4/LPC 31:4;O/LPE 34:4;O/LPS O-33:4/PC 31:3/PC O-31:4;O/PE 34:3/PE O-34:4;O	$C_{39}H_{73}NO_8P^+$	$C_{39}H_{72}NO_8PNa^+$	-
300	CerP 40:1;O6/LPS 34:0;O/PS O-34:0;O	$C_{40}H_{81}NO_{10}P^+$	-	-
301	CerP 40:2;O4/LPC 32:2;O/LPS O-34:2/PC 32:1/PC O-32:2;O/PE 35:1/PE O-35:2;O	$C_{40}H_{79}NO_8P^+$	$C_{40}H_{78}NO_8PNa^+$	-
302	CerP 40:2;O6/LPS 34:1;O/PS 34:0/PS O-34:1;O	$C_{40}H_{79}NO_{10}P^+$	-	-
303	CerP 40:3;O4/LPC 32:3;O/LPS O-34:3/PC 32:2/PC O-32:3;O/PE 35:2/PE O-35:3;O	$C_{40}H_{77}NO_8P^+$	$C_{40}H_{76}NO_8PNa^+$	$C_{40}H_{76}NO_8PK^+$
304	CerP 40:4;O4/LPC 32:4;O/LPS O-34:4/PC 32:3/PC O-32:4;O/PE 35:3/PE O-35:4;O	$C_{40}H_{75}NO_8P^+$	-	-
305	CerP 41:1;O6/PS O-35:0;O	$C_{41}H_{83}NO_{10}P^+$	-	-
306	CerP 41:2;O6/PS 35:0/PS O-35:1;O	$C_{41}H_{81}NO_{10}P^+$	-	$C_{41}H_{80}NO_{10}PK^+$
307	CerP 41:3;O4/LPC 33:3;O/PC 33:2/PC O-33:3;O/PE 36:2/PE O-36:3;O	$C_{41}H_{79}NO_8P^+$	$C_{41}H_{78}NO_8PNa^+$	-
308	CerP 41:3;O6/PS 35:1/PS O-35:2;O	-	-	$C_{41}H_{78}NO_{10}PK^+$
309	CerP 41:4;O4/LPC 33:4;O/PC 33:3/PC O-33:4;O/PE 36:3/PE O-36:4;O	$C_{41}H_{77}NO_8P^+$	-	-
310	CerP 41:4;O6/PS 35:2/PS O-35:3;O	$C_{41}H_{77}NO_{10}P^+$	-	-
311	CerP 41:6;O4/LPC 33:6;O/PC 33:5/PC O-33:6;O/PE 36:5/PE O-36:6;O	-	-	$C_{41}H_{72}NO_8PK^+$
312	CerP 42:2;O4/LPC 34:2;O/PC 34:1/PC O-34:2;O/PE 37:1/PE O-37:2;O	$C_{42}H_{83}NO_8P^+$	-	-
313	CerP 43:5;O6/PS 37:3/PS O-37:4;O	-	-	$C_{43}H_{78}NO_{10}PK^+$
314	CerP 46:2;O2	-	$C_{46}H_{90}NO_6PNa^+$	-
315	DG 20:0;O/DG O-20:1;O2/FA 23:1;O4	-	$C_{23}H_{44}O_6Na^+$	-
316	DG 20:0;O2	-	$C_{23}H_{44}O_7Na^+$	-
317	DG 20:1;O/DG O-20:2;O2/FA 23:2;O4	-	$C_{23}H_{42}O_6Na^+$	-
318	DG 20:1;O2	-	$C_{23}H_{42}O_7Na^+$	-
319	DG 20:2;O/DG O-20:3;O2/FA 23:3;O4/ST 23:0;O6	-	$C_{23}H_{40}O_6Na^+$	-
320	DG 20:2;O2/ST 23:0;O7	-	$C_{23}H_{40}O_7Na^+$	-
321	DG 20:3;O/DG O-20:4;O2/FA 23:4;O4/ST 23:1;O6	-	$C_{23}H_{38}O_6Na^+$	-
322	DG 20:3;O2/ST 23:1;O7	-	$C_{23}H_{38}O_7Na^+$	-
323	DG 21:0;O2	-	$C_{24}H_{46}O_7Na^+$	-
324	DG 21:1;O/DG O-21:2;O2/FA 24:2;O4	-	$C_{24}H_{44}O_6Na^+$	-
325	DG 21:1;O2	-	$C_{24}H_{44}O_7Na^+$	-

326	DG 21:2;O2/ST 24:0;O7	-	$C_{24}H_{42}O_7Na^+$	-
327	DG 21:3;O2/ST 18:0;O2;Hex/ST 24:1;O7	-	$C_{24}H_{40}O_7Na^+$	-
328	DG 21:5;O2/ST 18:1;O;GlCA/ST 18:2;O2;Hex/ST 24:3;O7	-	$C_{24}H_{36}O_7Na^+$	-
329	DG 22:0;O2	-	$C_{25}H_{48}O_7Na^+$	-
330	DG 22:1;O2	-	$C_{25}H_{46}O_7Na^+$	-
331	DG 22:2;O2/ST 25:0;O7	-	$C_{25}H_{44}O_7Na^+$	-
332	DG 23:1;O2	-	$C_{26}H_{48}O_7Na^+$	-
333	DG 23:2;O2/ST 26:0;O7	-	$C_{26}H_{46}O_7Na^+$	-
334	DG 23:3;O/DG O-23:4;O2/FA 26:4;O4/ST 20:0;O;Hex/ST 26:1;O6	-	$C_{26}H_{44}O_6Na^+$	-
335	DG 23:3;O2/ST 20:0;O2;Hex/ST 26:1;O7	-	$C_{26}H_{44}O_7Na^+$	-
336	DG 24:5/DG O-24:6;O/FA 27:6;O3/MG 24:6;O/ST 27:3;O5	-	-	$C_{27}H_{42}O_5K^+$
337	DG 28:5;O2/ST 25:1;O;GlCA/ST 25:2;O2;Hex	-	-	$C_{31}H_{50}O_7K^+$
338	DG 28:6;O2/ST 25:2;O;GlCA/ST 25:3;O2;Hex	-	-	$C_{31}H_{48}O_7K^+$
339	DG 30:5;O2/ST 27:1;O;GlCA/ST 27:2;O2;Hex/TG 30:4;O/TG O-30:5;O2	-	-	$C_{33}H_{54}O_7K^+$
340	DG 37:10/TG O-37:10	-	-	$C_{40}H_{58}O_5K^+$
341	DG 37:10;O2/TG 37:9;O/TG O-37:10;O2	-	-	$C_{40}H_{58}O_7K^+$
342	DG 41:10/DG O-41:11;O/TG O-41:10	-	-	$C_{44}H_{66}O_5K^+$
343	DG 41:10;O2/TG 41:9;O/TG O-41:10;O2	-	$C_{44}H_{66}O_7Na^+$	-
344	DG 41:11;O/TG 41:10/TG O-41:11;O	-	-	$C_{44}H_{64}O_6K^+$
345	DG 48:13/TG O-48:13	$C_{51}H_{75}O_5^+$	-	-
346	DG 49:14/TG O-49:14	-	-	$C_{52}H_{74}O_5K^+$
347	DG O-21:5/FA 24:5;O2/MG 21:5/MG O-21:6;O/ST 24:2;O4	$C_{24}H_{39}O_4^+$	-	-
348	DG O-32:3/MG 32:3/MG O-32:4;O	$C_{35}H_{65}O_4^+$	-	-
349	DG O-34:3/MG 34:3/MG O-34:4;O	$C_{37}H_{69}O_4^+$	$C_{37}H_{68}O_4Na^+$	-
350	DG O-34:4/MG 34:4/MG O-34:5;O	$C_{37}H_{67}O_4^+$	$C_{37}H_{66}O_4Na^+$	-
351	DG O-35:3	$C_{38}H_{71}O_4^+$	-	-
352	DG O-35:4	$C_{38}H_{69}O_4^+$	-	-
353	DG O-36:10	-	-	$C_{39}H_{58}O_4K^+$
354	DG O-36:4	$C_{39}H_{71}O_4^+$	-	-
355	DG O-44:11	-	-	$C_{47}H_{72}O_4K^+$
356	DG O-46:13	-	-	$C_{49}H_{72}O_4K^+$
357	FA 10:0	-	$C_{10}H_{20}O_2Na^+$	-
358	FA 10:0;O	$C_{10}H_{21}O_3^+$	$C_{10}H_{20}O_3Na^+$	$C_{10}H_{20}O_3K^+$
359	FA 10:1	$C_{10}H_{19}O_2^+$	$C_{10}H_{18}O_2Na^+$	-
360	FA 10:1;O	$C_{10}H_{19}O_3^+$	$C_{10}H_{18}O_3Na^+$	-
361	FA 10:1;O2	-	$C_{10}H_{18}O_4Na^+$	-
362	FA 10:2	$C_{10}H_{17}O_2^+$	-	-
363	FA 10:2;O	-	$C_{10}H_{16}O_3Na^+$	-
364	FA 10:2;O2	-	$C_{10}H_{16}O_4Na^+$	-
365	FA 11:0;O	-	$C_{11}H_{22}O_3Na^+$	-
366	FA 11:0;O2	-	$C_{11}H_{22}O_4Na^+$	-
367	FA 11:1	-	$C_{11}H_{20}O_2Na^+$	-
368	FA 11:1;O	-	$C_{11}H_{20}O_3Na^+$	-
369	FA 11:2;O	-	$C_{11}H_{18}O_3Na^+$	-
370	FA 11:2;O2	-	$C_{11}H_{18}O_4Na^+$	-
371	FA 12:0	$C_{12}H_{25}O_2^+$	$C_{12}H_{24}O_2Na^+$	-

372	FA 12:0;O	$C_{12}H_{25}O_3^+$	$C_{12}H_{24}O_3Na^+$	-
373	FA 12:0;O2	-	$C_{12}H_{24}O_4Na^+$	-
374	FA 12:0;O3	-	$C_{12}H_{24}O_5Na^+$	-
375	FA 12:0;O4	-	$C_{12}H_{24}O_6Na^+$	-
376	FA 12:1	$C_{12}H_{23}O_2^+$	$C_{12}H_{22}O_2Na^+$	-
377	FA 12:1;O	$C_{12}H_{23}O_3^+$	$C_{12}H_{22}O_3Na^+$	-
378	FA 12:1;O2	-	$C_{12}H_{22}O_4Na^+$	-
379	FA 12:1;O3	-	$C_{12}H_{22}O_5Na^+$	-
380	FA 12:2;O	-	$C_{12}H_{20}O_3Na^+$	-
381	FA 13:0;O/MG O-10:1	-	$C_{13}H_{26}O_3Na^+$	-
382	FA 13:0;O2/MG 10:0/MG O-10:1;O	-	$C_{13}H_{26}O_4Na^+$	-
383	FA 13:0;O3/MG 10:0;O	-	$C_{13}H_{26}O_5Na^+$	-
384	FA 13:0;O4	-	$C_{13}H_{26}O_6Na^+$	-
385	FA 13:1;O/MG O-10:2	-	$C_{13}H_{24}O_3Na^+$	-
386	FA 13:1;O2/MG 10:1/MG O-10:2;O	-	$C_{13}H_{24}O_4Na^+$	-
387	FA 13:1;O3/MG 10:1;O	-	$C_{13}H_{24}O_5Na^+$	-
388	FA 13:1;O4	-	$C_{13}H_{24}O_6Na^+$	-
389	FA 14:0;O2/MG 11:0/MG O-11:1;O	-	$C_{14}H_{28}O_4Na^+$	-
390	FA 14:0;O3/MG 11:0;O	-	$C_{14}H_{28}O_5Na^+$	-
391	FA 14:0;O4	-	$C_{14}H_{28}O_6Na^+$	-
392	FA 14:1;O/MG O-11:2	-	$C_{14}H_{26}O_3Na^+$	-
393	FA 14:1;O2/MG 11:1/MG O-11:2;O	-	$C_{14}H_{26}O_4Na^+$	-
394	FA 14:1;O3/MG 11:1;O	-	$C_{14}H_{26}O_5Na^+$	-
395	FA 14:1;O4	-	$C_{14}H_{26}O_6Na^+$	-
396	FA 14:2;O2/MG 11:2/MG O-11:3;O	-	$C_{14}H_{24}O_4Na^+$	-
397	FA 14:2;O3/MG 11:2;O	-	$C_{14}H_{24}O_5Na^+$	-
398	FA 14:2;O4	-	$C_{14}H_{24}O_6Na^+$	-
399	FA 14:3;O4	-	$C_{14}H_{22}O_6Na^+$	-
400	FA 15:0;O2/MG 12:0/MG O-12:1;O	-	$C_{15}H_{30}O_4Na^+$	-
401	FA 15:0;O3/MG 12:0;O	-	$C_{15}H_{30}O_5Na^+$	-
402	FA 15:0;O4	-	$C_{15}H_{30}O_6Na^+$	-
403	FA 15:1;O3/MG 12:1;O	-	$C_{15}H_{28}O_5Na^+$	-
404	FA 15:1;O4	-	$C_{15}H_{28}O_6Na^+$	-
405	FA 15:2;O4	-	$C_{15}H_{26}O_6Na^+$	-
406	FA 15:3;O4	-	$C_{15}H_{24}O_6Na^+$	-
407	FA 16:0	$C_{16}H_{33}O_2^+$	$C_{16}H_{32}O_2Na^+$	-
408	FA 16:0;O2/MG 13:0/MG O-13:1;O	-	$C_{16}H_{32}O_4Na^+$	-
409	FA 16:0;O3/MG 13:0;O	-	$C_{16}H_{32}O_5Na^+$	-
410	FA 16:0;O4	-	$C_{16}H_{32}O_6Na^+$	-
411	FA 16:1	$C_{16}H_{31}O_2^+$	$C_{16}H_{30}O_2Na^+$	-
412	FA 16:1;O/MG O-13:2	-	$C_{16}H_{30}O_3Na^+$	-
413	FA 16:1;O2/MG 13:1/MG O-13:2;O	-	$C_{16}H_{30}O_4Na^+$	-
414	FA 16:1;O3/MG 13:1;O	-	$C_{16}H_{30}O_5Na^+$	-
415	FA 16:1;O4	-	$C_{16}H_{30}O_6Na^+$	-
416	FA 16:2	$C_{16}H_{29}O_2^+$	-	-
417	FA 16:2;O2/MG 13:2/MG O-13:3;O	-	$C_{16}H_{28}O_4Na^+$	-
418	FA 16:2;O3/MG 13:2;O	-	$C_{16}H_{28}O_5Na^+$	-
419	FA 16:2;O4	-	$C_{16}H_{28}O_6Na^+$	-

420	FA 16:3;O2/MG 13:3	-	-	$C_{16}H_{26}O_4K^+$
421	FA 16:3;O3/MG 13:3;O	-	$C_{16}H_{26}O_5Na^+$	-
422	FA 16:3;O4	-	$C_{16}H_{26}O_6Na^+$	-
423	FA 17:0;O2/MG 14:0/MG O-14:1;O	-	$C_{17}H_{34}O_4Na^+$	-
424	FA 17:0;O3/MG 14:0;O	-	$C_{17}H_{34}O_5Na^+$	-
425	FA 17:0;O4	-	$C_{17}H_{34}O_6Na^+$	-
426	FA 17:1	$C_{17}H_{33}O_2^+$	-	-
427	FA 17:1;O2/MG 14:1/MG O-14:2;O	-	$C_{17}H_{32}O_4Na^+$	-
428	FA 17:1;O3/MG 14:1;O	-	$C_{17}H_{32}O_5Na^+$	-
429	FA 17:1;O4	-	$C_{17}H_{32}O_6Na^+$	-
430	FA 17:2;O3/MG 14:2;O	-	$C_{17}H_{30}O_5Na^+$	-
431	FA 17:2;O4	-	$C_{17}H_{30}O_6Na^+$	-
432	FA 17:3;O3/MG 14:3;O	-	$C_{17}H_{28}O_5Na^+$	-
433	FA 17:3;O4	-	$C_{17}H_{28}O_6Na^+$	-
434	FA 18:0;O2/MG 15:0/MG O-15:1;O	-	$C_{18}H_{36}O_4Na^+$	-
435	FA 18:0;O3/MG 15:0;O	-	$C_{18}H_{36}O_5Na^+$	-
436	FA 18:0;O4	-	$C_{18}H_{36}O_6Na^+$	-
437	FA 18:1	$C_{18}H_{35}O_2^+$	$C_{18}H_{34}O_2Na^+$	$C_{18}H_{34}O_2K^+$
438	FA 18:1;O2/MG 15:1/MG O-15:2;O	-	$C_{18}H_{34}O_4Na^+$	-
439	FA 18:1;O3/MG 15:1;O	-	$C_{18}H_{34}O_5Na^+$	-
440	FA 18:1;O4	-	$C_{18}H_{34}O_6Na^+$	-
441	FA 18:2	$C_{18}H_{33}O_2^+$	$C_{18}H_{32}O_2Na^+$	-
442	FA 18:2;O2/MG 15:2/MG O-15:3;O	-	$C_{18}H_{32}O_4Na^+$	-
443	FA 18:2;O3/MG 15:2;O	-	$C_{18}H_{32}O_5Na^+$	-
444	FA 18:2;O4	-	$C_{18}H_{32}O_6Na^+$	-
445	FA 18:3;O2/MG 15:3/MG O-15:4;O/ST 18:0;O4	-	-	$C_{18}H_{30}O_4K^+$
446	FA 18:3;O3/MG 15:3;O/ST 18:0;O5	-	$C_{18}H_{30}O_5Na^+$	-
447	FA 18:3;O4/ST 18:0;O6	-	$C_{18}H_{30}O_6Na^+$	-
448	FA 18:4;O4/ST 18:1;O6	-	$C_{18}H_{28}O_6Na^+$	-
449	FA 19:0;O2/MG 16:0/MG O-16:1;O	-	$C_{19}H_{38}O_4Na^+$	-
450	FA 19:0;O3/MG 16:0;O	-	$C_{19}H_{38}O_5Na^+$	-
451	FA 19:0;O4	-	$C_{19}H_{38}O_6Na^+$	-
452	FA 19:1	$C_{19}H_{37}O_2^+$	-	-
453	FA 19:1;O/MG O-16:2	$C_{19}H_{37}O_3^+$	-	-
454	FA 19:1;O2/MG 16:1/MG O-16:2;O	-	$C_{19}H_{36}O_4Na^+$	-
455	FA 19:1;O3/MG 16:1;O	-	$C_{19}H_{36}O_5Na^+$	-
456	FA 19:1;O4	-	$C_{19}H_{36}O_6Na^+$	-
457	FA 19:2;O/MG O-16:3	$C_{19}H_{35}O_3^+$	-	-
458	FA 19:2;O3/MG 16:2;O	-	$C_{19}H_{34}O_5Na^+$	-
459	FA 19:2;O4	-	$C_{19}H_{34}O_6Na^+$	-
460	FA 19:3;O3/MG 16:3;O/ST 19:0;O5	-	$C_{19}H_{32}O_5Na^+$	-
461	FA 19:3;O4/ST 19:0;O6	-	$C_{19}H_{32}O_6Na^+$	-
462	FA 19:4;O4/ST 19:1;O6	-	$C_{19}H_{30}O_6Na^+$	-
463	FA 20:0;O3/MG 17:0;O	-	$C_{20}H_{40}O_5Na^+$	-
464	FA 20:0;O4	-	$C_{20}H_{40}O_6Na^+$	-
465	FA 20:1;O3/MG 17:1;O	$C_{20}H_{39}O_5^+$	$C_{20}H_{38}O_5Na^+$	-
466	FA 20:1;O4	-	$C_{20}H_{38}O_6Na^+$	-
467	FA 20:2;O3/MG 17:2;O	-	$C_{20}H_{36}O_5Na^+$	-

468	FA 20:2;O4	-	$C_{20}H_{36}O_6Na^+$	-
469	FA 20:3;O4/ST 20:0;O6	-	$C_{20}H_{34}O_6Na^+$	-
470	FA 20:4;O4/ST 20:1;O6	-	$C_{20}H_{32}O_6Na^+$	-
471	FA 21:0;O3/MG 18:0;O	-	$C_{21}H_{42}O_5Na^+$	-
472	FA 21:0;O4	-	$C_{21}H_{42}O_6Na^+$	-
473	FA 21:1;O3/MG 18:1;O	-	$C_{21}H_{40}O_5Na^+$	-
474	FA 21:1;O4	-	$C_{21}H_{40}O_6Na^+$	-
475	FA 21:2;O/MG O-18:3	$C_{21}H_{39}O_3^+$	$C_{21}H_{38}O_3Na^+$	-
476	FA 21:2;O3/MG 18:2;O	-	$C_{21}H_{38}O_5Na^+$	-
477	FA 21:2;O4	-	$C_{21}H_{38}O_6Na^+$	-
478	FA 21:3;O3/MG 18:3;O/ST 21:0;O5	-	$C_{21}H_{36}O_5Na^+$	-
479	FA 21:3;O4/ST 21:0;O6	-	$C_{21}H_{36}O_6Na^+$	-
480	FA 21:4;O4/ST 21:1;O6	-	$C_{21}H_{34}O_6Na^+$	-
481	FA 22:0;O3/MG 19:0;O	-	$C_{22}H_{44}O_5Na^+$	-
482	FA 22:0;O4	-	$C_{22}H_{44}O_6Na^+$	-
483	FA 22:1;O3/MG 19:1;O	$C_{22}H_{43}O_5^+$	$C_{22}H_{42}O_5Na^+$	-
484	FA 22:1;O4	-	$C_{22}H_{42}O_6Na^+$	-
485	FA 22:2;O2/MG 19:2/MG O-19:3;O	$C_{22}H_{41}O_4^+$	-	-
486	FA 22:2;O3/MG 19:2;O	$C_{22}H_{41}O_5^+$	$C_{22}H_{40}O_5Na^+$	$C_{22}H_{40}O_5K^+$
487	FA 22:2;O4	-	$C_{22}H_{40}O_6Na^+$	-
488	FA 22:3;O4/ST 22:0;O6	-	$C_{22}H_{38}O_6Na^+$	-
489	FA 22:4;O4/ST 22:1;O6	-	$C_{22}H_{36}O_6Na^+$	-
490	FA 25:7;O2/ST 25:4;O4	-	$C_{25}H_{36}O_4Na^+$	-
491	Hex2Cer 28:2;O4	-	-	$C_{40}H_{73}NO_{15}K^+$
492	Hex2Cer 28:5;O3	-	-	$C_{40}H_{67}NO_{14}K^+$
493	Hex2Cer 30:2;O3	-	-	$C_{42}H_{77}NO_{14}K^+$
494	Hex2Cer 30:3;O2	-	-	$C_{42}H_{75}NO_{13}K^+$
495	Hex2Cer 30:5;O3	-	$C_{42}H_{71}NO_{14}Na^+$	$C_{42}H_{71}NO_{14}K^+$
496	Hex2Cer 30:6;O3	-	-	$C_{42}H_{69}NO_{14}K^+$
497	Hex2Cer 32:6;O3	-	-	$C_{44}H_{73}NO_{14}K^+$
498	Hex2Cer 42:2;O2	$C_{54}H_{102}NO_{13}^+$	-	-
499	HexCer 28:6;O4/ST 26:2;O5;HexNAC	$C_{34}H_{56}NO_{10}^+$	-	-
500	HexCer 30:1;O4	-	$C_{36}H_{69}NO_{10}Na^+$	-
501	HexCer 33:1;O6	-	$C_{39}H_{75}NO_{12}Na^+$	-
502	HexCer 33:2;O3	-	-	$C_{39}H_{73}NO_9K^+$
503	HexCer 34:1;O5	-	-	$C_{40}H_{77}NO_{11}K^+$
504	HexCer 35:5;O6	$C_{41}H_{72}NO_{12}^+$	-	-
505	HexCer 37:6;O4	-	$C_{43}H_{73}NO_{10}Na^+$	-
506	HexCer 48:4;O6	$C_{54}H_{100}NO_{12}^+$	-	-
507	HexCer 48:5;O5	$C_{54}H_{98}NO_{11}^+$	-	-
508	HexCer 48:6;O4	$C_{54}H_{96}NO_{10}^+$	-	-
509	LPA 17:1/LPA O-17:2;O	$C_{20}H_{40}O_7P^+$	-	-
510	LPA 18:2/LPA O-18:3;O	-	-	$C_{21}H_{39}O_7PK^+$
511	LPA 18:2;O/LPG O-15:3	-	-	$C_{21}H_{39}O_8PK^+$
512	LPA 21:0;O/LPG O-18:1/PA O-21:0;O	-	$C_{24}H_{49}O_8PNa^+$	-
513	LPA 22:5/LPA O-22:6;O/PA O-22:5	$C_{25}H_{42}O_7P^+$	-	-
514	LPA 30:7/PA O-30:7	-	-	$C_{33}H_{53}O_7PK^+$
515	LPA 32:2/LPA O-32:3;O/PA O-32:2	-	-	$C_{35}H_{67}O_7PK^+$

516	LPA 34:2/LPA O-34:3;O/PA O-34:2	-	-	$C_{37}H_{71}O_7PK^+$
517	LPA 34:3/LPA O-34:4;O/PA O-34:3	-	-	$C_{37}H_{69}O_7PK^+$
518	LPA O-11:1	-	-	$C_{14}H_{29}O_6PK^+$
519	LPA O-11:3	$C_{14}H_{26}O_6P^+$	-	-
520	LPA O-13:3	$C_{16}H_{30}O_6P^+$	-	-
521	LPA O-14:2	-	-	$C_{17}H_{33}O_6PK^+$
522	LPC 10:2;O/LPE 13:2;O/LPS O-12:2	$C_{18}H_{35}NO_8P^+$	-	-
523	LPC 11:0;O/LPE 14:0;O/LPS O-13:0	-	$C_{19}H_{40}NO_8PNa^+$	-
524	LPC 13:0/LPC O-13:1;O/LPE 16:0/LPE O-16:1;O	$C_{21}H_{45}NO_7P^+$	$C_{21}H_{44}NO_7PNa^+$	-
525	LPC 13:1/LPC O-13:2;O/LPE 16:1/LPE O-16:2;O	$C_{21}H_{43}NO_7P^+$	-	-
526	LPC 14:0;O/LPE 17:0;O/LPS O-16:0	-	$C_{22}H_{46}NO_8PNa^+$	-
527	LPC 14:1/LPC O-14:2;O/LPE 17:1/LPE O-17:2;O	-	-	$C_{22}H_{44}NO_7PK^+$
528	LPC 15:1/LPC O-15:2;O/LPE 18:1/LPE O-18:2;O	$C_{23}H_{47}NO_7P^+$	$C_{23}H_{46}NO_7PNa^+$	-
529	LPC 15:2/LPC O-15:3;O/LPE 18:2/LPE O-18:3;O	$C_{23}H_{45}NO_7P^+$	-	-
530	LPC 15:2;O/LPE 18:2;O/LPS O-17:2	$C_{23}H_{45}NO_8P^+$	-	-
531	LPC 16:1/LPC O-16:2;O/LPE 19:1/LPE O-19:2;O	$C_{24}H_{49}NO_7P^+$	-	-
532	LPC 16:1;O/LPE 19:1;O/LPS O-18:1	$C_{24}H_{49}NO_8P^+$	-	-
533	LPC 16:2;O/LPE 19:2;O/LPS O-18:2	$C_{24}H_{47}NO_8P^+$	-	-
534	LPC 17:2;O/LPE 20:2;O/LPS O-19:2/PE 20:1/PE O-20:2;O	$C_{25}H_{49}NO_8P^+$	-	-
535	LPC 17:3;O/LPE 20:3;O/LPS O-19:3/PE 20:2/PE O-20:3;O	$C_{25}H_{47}NO_8P^+$	-	-
536	LPC 18:2;O/LPE 21:2;O/LPS O-20:2/PE 21:1/PE O-21:2;O	$C_{26}H_{51}NO_8P^+$	$C_{26}H_{50}NO_8PNa^+$	-
537	LPC 18:3;O/LPE 21:3;O/LPS O-20:3/PE 21:2/PE O-21:3;O	$C_{26}H_{49}NO_8P^+$	-	-
538	LPC 19:0;O/LPE 22:0;O/LPS O-21:0/PE O-22:0;O	-	$C_{27}H_{56}NO_8PNa^+$	-
539	LPC 19:2;O/LPE 22:2;O/LPS O-21:2/PE 22:1/PE O-22:2;O	$C_{27}H_{53}NO_8P^+$	-	-
540	LPC 27:7;O/LPE 30:7;O/LPS O-29:7/PC 27:6/PC O-27:7;O/PE 30:6/PE O-30:7;O	-	$C_{35}H_{58}NO_8PNa^+$	-
541	LPC 29:7;O/LPE 32:7;O/LPS O-31:7/PC 29:6/PC O-29:7;O/PE 32:6/PE O-32:7;O	$C_{37}H_{63}NO_8P^+$	$C_{37}H_{62}NO_8PNa^+$	$C_{37}H_{62}NO_8PK^+$
542	LPC 32:7/PC O-32:7/PE O-35:7	-	$C_{40}H_{68}NO_7PNa^+$	-
543	LPC 34:7/PC O-34:7/PE O-37:7	-	$C_{42}H_{72}NO_7PNa^+$	-
544	LPC O-13:2/LPE O-16:2	$C_{21}H_{43}NO_6P^+$	-	-
545	LPC O-13:3/LPE O-16:3	$C_{21}H_{41}NO_6P^+$	-	-
546	LPC O-15:3/LPE O-18:3	$C_{23}H_{45}NO_6P^+$	-	-
547	LPE 10:0/LPE O-10:1;O/SPBP 15:1;O4	-	-	$C_{15}H_{32}NO_7PK^+$
548	LPE 10:0;O	-	$C_{15}H_{32}NO_8PNa^+$	-
549	LPE 11:0;O/LPS O-10:0	-	$C_{16}H_{34}NO_8PNa^+$	-
550	LPE 11:2;O/LPS O-10:2	-	$C_{16}H_{30}NO_8PNa^+$	-
551	LPE O-10:0;O/SPBP 15:0;O4	-	$C_{15}H_{34}NO_7PNa^+$	-
552	LPE O-10:1/SPBP 15:1;O3	-	-	$C_{15}H_{32}NO_6PK^+$
553	LPE O-10:2/SPBP 15:2;O3	-	-	$C_{15}H_{30}NO_6PK^+$
554	LPG 11:0;O	-	$C_{17}H_{35}O_{10}PNa^+$	-
555	LPG 13:0;O	$C_{19}H_{40}O_{10}P^+$	-	-
556	LPG 14:1/LPG O-14:2;O	-	-	$C_{20}H_{39}O_9PK^+$
557	LPG 16:3/LPG O-16:4;O	-	-	$C_{22}H_{39}O_9PK^+$
558	LPG 19:2/LPG O-19:3;O/PA 22:1;O	-	$C_{25}H_{47}O_9PNa^+$	-
559	LPG 20:1/LPG O-20:2;O/PA 23:0;O/PG O-20:1	-	$C_{26}H_{51}O_9PNa^+$	-
560	LPG 21:0/LPG O-21:1;O/PG O-21:0	-	$C_{27}H_{55}O_9PNa^+$	-
561	LPG 21:0;O/PG O-21:0;O	-	$C_{27}H_{55}O_{10}PNa^+$	-

562	LPG 21:5/LPG O-21:6;O/PA 24:4;O/PG O-21:5	$C_{27}H_{46}O_9P^+$	-	-
563	LPG 23:5/LPG O-23:6;O/PA 26:4;O/PG O-23:5	$C_{29}H_{50}O_9P^+$	-	-
564	LPG 23:6/PA 26:5;O/PG O-23:6	$C_{29}H_{48}O_9P^+$	-	-
565	LPG 25:5/LPG O-25:6;O/PA 28:4;O/PG O-25:5	$C_{31}H_{54}O_9P^+$	-	-
566	LPG O-10:0;O	-	$C_{16}H_{35}O_9PNa^+$	-
567	LPG O-11:0;O	-	$C_{17}H_{37}O_9PNa^+$	-
568	LPI 10:0;O	-	$C_{19}H_{37}O_{13}PNa^+$	-
569	LPI 10:1/LPI O-10:2;O	-	$C_{19}H_{35}O_{12}PNa^+$	-
570	LPI 11:0/LPI O-11:1;O	$C_{20}H_{40}O_{12}P^+$	-	-
571	LPI 11:0;O	$C_{20}H_{40}O_{13}P^+$	-	-
572	LPI 11:1;O	-	$C_{20}H_{37}O_{13}PNa^+$	-
573	LPI 12:0;O	-	$C_{21}H_{41}O_{13}PNa^+$	-
574	LPI 13:0;O	$C_{22}H_{44}O_{13}P^+$	$C_{22}H_{43}O_{13}PNa^+$	-
575	LPI 13:1;O	-	$C_{22}H_{41}O_{13}PNa^+$	-
576	LPI 14:0;O	$C_{23}H_{46}O_{13}P^+$	-	-
577	LPI 14:1;O	-	$C_{23}H_{43}O_{13}PNa^+$	-
578	LPI 15:1/LPI O-15:2;O	-	$C_{24}H_{45}O_{12}PNa^+$	-
579	LPI 16:1/LPI O-16:2;O	-	$C_{25}H_{47}O_{12}PNa^+$	-
580	LPI 16:2;O	-	$C_{25}H_{45}O_{13}PNa^+$	-
581	LPI 17:0;O	-	$C_{26}H_{51}O_{13}PNa^+$	-
582	LPI 19:0/LPI O-19:1;O	-	$C_{28}H_{55}O_{12}PNa^+$	-
583	LPI 19:0;O	-	$C_{28}H_{55}O_{13}PNa^+$	-
584	LPI 19:2/LPI O-19:3;O	-	$C_{28}H_{51}O_{12}PNa^+$	-
585	LPI 20:0;O/PI O-20:0;O	-	$C_{29}H_{57}O_{13}PNa^+$	-
586	LPI 20:2/LPI O-20:3;O/PI O-20:2	-	$C_{29}H_{53}O_{12}PNa^+$	-
587	LPI 21:1/LPI O-21:2;O/PI O-21:1	-	$C_{30}H_{57}O_{12}PNa^+$	-
588	LPI 28:4;O/PI 28:3/PI O-28:4;O	-	-	$C_{37}H_{65}O_{13}PK^+$
589	LPI 29:0;O/PI O-29:0;O	$C_{38}H_{76}O_{13}P^+$	-	-
590	LPI 31:7/PI O-31:7	$C_{40}H_{66}O_{12}P^+$	-	-
591	LPI 32:1/LPI O-32:2;O/PI O-32:1	-	$C_{41}H_{79}O_{12}PNa^+$	-
592	LPI 32:2/LPI O-32:3;O/PI O-32:2	-	$C_{41}H_{77}O_{12}PNa^+$	-
593	LPI 33:6;O/PI 33:5/PI O-33:6;O	$C_{42}H_{72}O_{13}P^+$	-	-
594	LPI 34:5/LPI O-34:6;O/PI O-34:5	$C_{43}H_{76}O_{12}P^+$	-	-
595	LPI O-10:0	-	$C_{19}H_{39}O_{11}PNa^+$	-
596	LPI O-10:0;O	$C_{19}H_{40}O_{12}P^+$	$C_{19}H_{39}O_{12}PNa^+$	-
597	LPI O-11:0	-	$C_{20}H_{41}O_{11}PNa^+$	-
598	LPI O-11:0;O	$C_{20}H_{42}O_{12}P^+$	$C_{20}H_{41}O_{12}PNa^+$	-
599	LPI O-12:0	-	$C_{21}H_{43}O_{11}PNa^+$	-
600	LPI O-13:0	-	$C_{22}H_{45}O_{11}PNa^+$	-
601	LPI O-15:0	-	$C_{24}H_{49}O_{11}PNa^+$	-
602	LPI O-15:0;O	$C_{24}H_{50}O_{12}P^+$	-	-
603	LPI O-16:0	-	$C_{25}H_{51}O_{11}PNa^+$	-
604	LPI O-16:0;O	-	$C_{25}H_{51}O_{12}PNa^+$	-
605	LPI O-17:0	-	$C_{26}H_{53}O_{11}PNa^+$	-
606	LPI O-17:0;O	$C_{26}H_{54}O_{12}P^+$	$C_{26}H_{53}O_{12}PNa^+$	-
607	LPI O-18:0;O	-	$C_{27}H_{55}O_{12}PNa^+$	-
608	LPI O-19:0	-	$C_{28}H_{57}O_{11}PNa^+$	-
609	LPI O-19:0;O	-	$C_{28}H_{57}O_{12}PNa^+$	-

610	LPI O-20:0	-	$C_{29}H_{59}O_{11}PNa^+$	-
611	LPI O-20:0;O	-	$C_{29}H_{59}O_{12}PNa^+$	-
612	LPI O-24:0	-	$C_{33}H_{67}O_{11}PNa^+$	-
613	LPS 10:0/LPS O-10:1;O	-	$C_{16}H_{32}NO_9PNa^+$	-
614	LPS 10:0;O	-	$C_{16}H_{32}NO_{10}PNa^+$	-
615	LPS 11:0;O	-	$C_{17}H_{34}NO_{10}PNa^+$	-
616	LPS 13:0;O	-	$C_{19}H_{38}NO_{10}PNa^+$	-
617	LPS 18:0;O	-	$C_{24}H_{48}NO_{10}PNa^+$	-
618	LPS 34:7/PC 32:7;O/PE 35:7;O/PS O-34:7	-	-	$C_{40}H_{66}NO_9PK^+$
619	LPS O-10:0;O	-	$C_{16}H_{34}NO_9PNa^+$	-
620	LPS O-11:0;O	-	$C_{17}H_{36}NO_9PNa^+$	-
621	LPS O-12:0;O	-	$C_{18}H_{38}NO_9PNa^+$	-
622	LPS O-16:0;O	-	$C_{22}H_{46}NO_9PNa^+$	-
623	LPS O-19:0;O	-	$C_{25}H_{52}NO_9PNa^+$	-
624	LPS O-20:0;O	-	$C_{26}H_{54}NO_9PNa^+$	-
625	MG O-10:0;O	-	$C_{13}H_{28}O_4Na^+$	-
626	MG O-11:0	-	$C_{14}H_{30}O_3Na^+$	-
627	MG O-11:0;O	-	$C_{14}H_{30}O_4Na^+$	-
628	MG O-12:0;O	-	$C_{15}H_{32}O_4Na^+$	-
629	MG O-13:0;O	-	$C_{16}H_{34}O_4Na^+$	-
630	MG O-14:0;O	-	$C_{17}H_{36}O_4Na^+$	-
631	MG O-15:0;O	-	$C_{18}H_{38}O_4Na^+$	-
632	MG O-16:0;O	-	$C_{19}H_{40}O_4Na^+$	-
633	MIPC 28:1;O2	$C_{40}H_{77}NO_{16}P^+$	-	-
634	MIPC 28:4;O2	$C_{40}H_{71}NO_{16}P^+$	-	-
635	MIPC 28:5;O2	$C_{40}H_{69}NO_{16}P^+$	-	-
636	MIPC 30:5;O2	$C_{42}H_{73}NO_{16}P^+$	-	-
637	NAE 10:0	$C_{12}H_{26}NO_2^+$	-	-
638	NAE 10:0;O	$C_{12}H_{26}NO_3^+$	-	-
639	NAE 10:0;O2	$C_{12}H_{26}NO_4^+$	-	-
640	NAE 10:0;O3	-	$C_{12}H_{25}NO_5Na^+$	-
641	NAE 10:1	$C_{12}H_{24}NO_2^+$	-	-
642	NAE 10:1;O	$C_{12}H_{24}NO_3^+$	-	-
643	NAE 10:1;O2	$C_{12}H_{24}NO_4^+$	-	-
644	NAE 10:2	$C_{12}H_{22}NO_2^+$	-	-
645	NAE 10:2;O	$C_{12}H_{22}NO_3^+$	-	-
646	NAE 11:0	$C_{13}H_{28}NO_2^+$	-	-
647	NAE 11:0;O	$C_{13}H_{28}NO_3^+$	-	-
648	NAE 11:0;O2	$C_{13}H_{28}NO_4^+$	-	-
649	NAE 11:0;O3	-	$C_{13}H_{27}NO_5Na^+$	-
650	NAE 11:0;O4	-	$C_{13}H_{27}NO_6Na^+$	-
651	NAE 11:1	$C_{13}H_{26}NO_2^+$	-	-
652	NAE 11:1;O	$C_{13}H_{26}NO_3^+$	$C_{13}H_{25}NO3Na^+$	$C_{13}H_{25}NO3K^+$
653	NAE 11:1;O2	$C_{13}H_{26}NO_4^+$	-	-
654	NAE 11:1;O3	-	$C_{13}H_{25}NO_5Na^+$	-
655	NAE 11:2	$C_{13}H_{24}NO_2^+$	-	-
656	NAE 11:2;O	$C_{13}H_{24}NO_3^+$	-	-
657	NAE 11:3	$C_{13}H_{22}NO_2^+$	-	-

658	NAE 12:0/SPB 14:1;O2	$C_{14}H_{30}NO_2^+$	-	-
659	NAE 12:0;O/SPB 14:1;O3	$C_{14}H_{30}NO_3^+$	-	-
660	NAE 12:0;O2/SPB 14:1;O4	$C_{14}H_{30}NO_4^+$	$C_{14}H_{29}NO_4Na^+$	-
661	NAE 12:0;O3	-	$C_{14}H_{29}NO_5Na^+$	-
662	NAE 12:0;O4	-	$C_{14}H_{29}NO_6Na^+$	-
663	NAE 12:1/SPB 14:2;O2	$C_{14}H_{28}NO_2^+$	-	-
664	NAE 12:1;O/SPB 14:2;O3	$C_{14}H_{28}NO_3^+$	-	-
665	NAE 12:1;O2/SPB 14:2;O4	$C_{14}H_{28}NO_4^+$	$C_{14}H_{27}NO_4Na^+$	-
666	NAE 12:1;O3	$C_{14}H_{28}NO_5^+$	$C_{14}H_{27}NO_5Na^+$	-
667	NAE 12:1;O4	$C_{14}H_{28}NO_6^+$	$C_{14}H_{27}NO_6Na^+$	-
668	NAE 12:2/SPB 14:3;O2	$C_{14}H_{26}NO_2^+$	-	-
669	NAE 12:2;O2/SPB 14:3;O4	-	$C_{14}H_{25}NO_4Na^+$	-
670	NAE 12:2;O4	-	$C_{14}H_{25}NO_6Na^+$	-
671	NAE 12:3	$C_{14}H_{24}NO_2^+$	-	-
672	NAE 12:3;O4	-	-	$C_{14}H_{23}NO_6K^+$
673	NAE 13:0/SPB 15:1;O2	$C_{15}H_{32}NO_2^+$	-	-
674	NAE 13:0;O/SPB 15:1;O3	$C_{15}H_{32}NO_3^+$	-	-
675	NAE 13:0;O2/SPB 15:1;O4	-	$C_{15}H_{31}NO_4Na^+$	-
676	NAE 13:0;O3	-	$C_{15}H_{31}NO_5Na^+$	-
677	NAE 13:0;O4	$C_{15}H_{32}NO_6^+$	$C_{15}H_{31}NO_6Na^+$	-
678	NAE 13:1/SPB 15:2;O2	$C_{15}H_{30}NO_2^+$	-	$C_{15}H_{29}NO_2K^+$
679	NAE 13:1;O/SPB 15:2;O3	$C_{15}H_{30}NO_3^+$	-	$C_{15}H_{29}NO_3K^+$
680	NAE 13:1;O3	-	$C_{15}H_{29}NO_5Na^+$	-
681	NAE 13:1;O4	$C_{15}H_{30}NO_6^+$	$C_{15}H_{29}NO_6Na^+$	-
682	NAE 13:2/SPB 15:3;O2	$C_{15}H_{28}NO_2^+$	-	$C_{15}H_{27}NO_2K^+$
683	NAE 13:2;O/SPB 15:3;O3	$C_{15}H_{28}NO_3^+$	-	$C_{15}H_{27}NO_3K^+$
684	NAE 13:2;O2/SPB 15:3;O4	-	$C_{15}H_{27}NO_4Na^+$	-
685	NAE 13:2;O3	-	$C_{15}H_{27}NO_5Na^+$	-
686	NAE 13:2;O4	-	$C_{15}H_{27}NO_6Na^+$	-
687	NAE 13:3	$C_{15}H_{26}NO_2^+$	-	-
688	NAE 13:3;O4	-	$C_{15}H_{25}NO_6Na^+$	-
689	NAE 14:0/SPB 16:1;O2	$C_{16}H_{34}NO_2^+$	-	-
690	NAE 14:0;O/SPB 16:1;O3	$C_{16}H_{34}NO_3^+$	-	-
691	NAE 14:0;O3	-	$C_{16}H_{33}NO_5Na^+$	-
692	NAE 14:0;O4	-	$C_{16}H_{33}NO_6Na^+$	-
693	NAE 14:1/SPB 16:2;O2	$C_{16}H_{32}NO_2^+$	-	-
694	NAE 14:1;O/SPB 16:2;O3	$C_{16}H_{32}NO_3^+$	-	-
695	NAE 14:1;O3	-	$C_{16}H_{31}NO_5Na^+$	-
696	NAE 14:1;O4	-	$C_{16}H_{31}NO_6Na^+$	-
697	NAE 14:2/SPB 16:3;O2	$C_{16}H_{30}NO_2^+$	-	-
698	NAE 14:2;O/SPB 16:3;O3	$C_{16}H_{30}NO_3^+$	-	-
699	NAE 14:2;O2/SPB 16:3;O4	-	$C_{16}H_{29}NO_4Na^+$	-
700	NAE 14:2;O3	-	$C_{16}H_{29}NO_5Na^+$	-
701	NAE 14:2;O4	-	$C_{16}H_{29}NO_6Na^+$	-
702	NAE 14:3	$C_{16}H_{28}NO_2^+$	-	-
703	NAE 14:3;O	$C_{16}H_{28}NO_3^+$	-	-
704	NAE 14:3;O3	-	$C_{16}H_{27}NO_5Na^+$	-
705	NAE 14:3;O4	-	$C_{16}H_{27}NO_6Na^+$	-

706	NAE 14:4	$C_{16}H_{26}NO_2^+$	-	-
707	NAE 14:4;O4	-	$C_{16}H_{25}NO_6Na^+$	-
708	NAE 15:0/SPB 17:1;O2	$C_{17}H_{36}NO_2^+$	-	-
709	NAE 15:0;O3	-	$C_{17}H_{35}NO_5Na^+$	-
710	NAE 15:0;O4	-	$C_{17}H_{35}NO_6Na^+$	-
711	NAE 15:1/SPB 17:2;O2	$C_{17}H_{34}NO_2^+$	-	-
712	NAE 15:2/SPB 17:3;O2	$C_{17}H_{32}NO_2^+$	-	$C_{17}H_{31}NO_2K^+$
713	NAE 15:2;O/SPB 17:3;O3	-	-	$C_{17}H_{31}NO_3K^+$
714	NAE 15:3	$C_{17}H_{30}NO_2^+$	-	-
715	NAE 15:3;O	$C_{17}H_{30}NO_3^+$	-	-
716	NAE 15:4	$C_{17}H_{28}NO_2^+$	-	-
717	NAE 16:0/SPB 18:1;O2	$C_{18}H_{38}NO_2^+$	-	-
718	NAE 16:0;O3	-	$C_{18}H_{37}NO_5Na^+$	-
719	NAE 16:0;O4	-	$C_{18}H_{37}NO_6Na^+$	-
720	NAE 16:1/SPB 18:2;O2	$C_{18}H_{36}NO_2^+$	-	-
721	NAE 16:2/SPB 18:3;O2	$C_{18}H_{34}NO_2^+$	-	-
722	NAE 17:0/SPB 19:1;O2	$C_{19}H_{40}NO_2^+$	-	-
723	NAE 17:0;O/SPB 19:1;O3	$C_{19}H_{40}NO_3^+$	-	-
724	NAE 17:0;O3	-	$C_{19}H_{39}NO_5Na^+$	-
725	NAE 17:0;O4	-	$C_{19}H_{39}NO_6Na^+$	-
726	NAE 17:1/SPB 19:2;O2	$C_{19}H_{38}NO_2^+$	-	-
727	NAE 18:0;O3	-	$C_{20}H_{41}NO_5Na^+$	-
728	NAE 18:0;O4	-	$C_{20}H_{41}NO_6Na^+$	-
729	NAE 18:1;O/SPB 20:2;O3	$C_{20}H_{40}NO_3^+$	-	$C_{20}H_{39}NO_3K^+$
730	NAE 18:5;O4/ST 18:1;O5;G	-	$C_{20}H_{31}NO_6Na^+$	-
731	NAE 19:0;O3	-	$C_{21}H_{43}NO_5Na^+$	-
732	NAE 19:0;O4	-	$C_{21}H_{43}NO_6Na^+$	-
733	NAE 19:1	$C_{21}H_{42}NO_2^+$	-	-
734	NAE 19:1;O	$C_{21}H_{42}NO_3^+$	-	-
735	NAE 19:2	$C_{21}H_{40}NO_2^+$	-	-
736	NAE 19:3	$C_{21}H_{38}NO_2^+$	-	-
737	NAE 20:0	$C_{22}H_{46}NO_2^+$	-	-
738	NAE 20:4	$C_{22}H_{38}NO_2^+$	-	-
739	NAE 21:0	$C_{23}H_{48}NO_2^+$	-	-
740	NAE 21:2	$C_{23}H_{44}NO_2^+$	-	-
741	NAE 21:3	$C_{23}H_{42}NO_2^+$	-	-
742	NAE 21:3;O	$C_{23}H_{42}NO_3^+$	-	-
743	NAE 21:4	$C_{23}H_{40}NO_2^+$	-	-
744	NAE 21:6	-	$C_{23}H_{35}NO_2Na^+$	-
745	NAE 22:0	$C_{24}H_{50}NO_2^+$	-	-
746	NAT 12:1;O3	-	$C_{14}H_{27}NO_7SNa^+$	-
747	NAT 12:2;O2	-	$C_{14}H_{25}NO_6SNa^+$	-
748	NAT 12:2;O3	$C_{14}H_{26}NO_7S^+$	-	-
749	NAT 17:0;O3	-	-	$C_{19}H_{39}NO_7SK^+$
750	NAT 18:4	-	-	$C_{20}H_{33}NO_4SK^+$
751	NAT 20:5;O/ST 20:2;O3;T	-	-	$C_{22}H_{35}NO_5SK^+$
752	NAT 20:5;O2/ST 20:2;O4;T	-	$C_{22}H_{35}NO_6SNa^+$	-
753	NAT 22:5;O2/ST 22:2;O4;T	-	-	$C_{24}H_{39}NO_6SK^+$

754	NAT 22:6;O/ST 22:3;O3;T	-	-	$C_{24}H_{37}NO_5SK^+$
755	NAT 23:5;O2/ST 23:2;O4;T	-	-	$C_{25}H_{41}NO_6SK^+$
756	NAT 24:5;O4/ST 24:2;O6;T	-	-	$C_{26}H_{43}NO_8SK^+$
757	NAT 24:6	-	-	$C_{26}H_{41}NO_4SK^+$
758	NAT 24:6;O/ST 24:3;O3;T	-	-	$C_{26}H_{41}NO_5SK^+$
759	NAT 24:6;O2/ST 24:3;O4;T	-	-	$C_{26}H_{41}NO_6SK^+$
760	NAT 25:5;O3/ST 25:2;O5;T	-	-	$C_{27}H_{45}NO_7SK^+$
761	NAT 25:6	-	-	$C_{27}H_{43}NO_4SK^+$
762	NAT 25:7	-	-	$C_{27}H_{41}NO_4SK^+$
763	NAT 26:4;O3/ST 26:1;O5;T	-	-	$C_{28}H_{49}NO_7SK^+$
764	NAT 26:5;O3/ST 26:2;O5;T	-	-	$C_{28}H_{47}NO_7SK^+$
765	NAT 26:6;O3/ST 26:3;O5;T	-	-	$C_{28}H_{45}NO_7SK^+$
766	NAT 26:7	-	-	$C_{28}H_{43}NO_4SK^+$
767	NAT 26:7;O/ST 26:4;O3;T	-	-	$C_{28}H_{43}NO_5SK^+$
768	NAT 27:4;O2/ST 27:1;O4;T	-	-	$C_{29}H_{51}NO_6SK^+$
769	NAT 27:4;O4/ST 27:1;O6;T	-	-	$C_{29}H_{51}NO_8SK^+$
770	NAT 27:5	-	-	$C_{29}H_{49}NO_4SK^+$
771	NAT 27:5;O2/ST 27:2;O4;T	-	-	$C_{29}H_{49}NO_6SK^+$
772	NAT 27:7;O/ST 27:4;O3;T	-	-	$C_{29}H_{45}NO_5SK^+$
773	NAT 27:7;O2/ST 27:4;O4;T	-	-	$C_{29}H_{45}NO_6SK^+$
774	NAT 28:4;O/ST 28:1;O3;T	-	-	$C_{30}H_{53}NO_5SK^+$
775	NAT 28:4;O3/ST 28:1;O5;T	-	-	$C_{30}H_{53}NO_7SK^+$
776	NAT 28:5;O/ST 28:2;O3;T	-	-	$C_{30}H_{51}NO_5SK^+$
777	NAT 28:6;O/ST 28:3;O3;T	-	-	$C_{30}H_{49}NO_5SK^+$
778	NAT 28:6;O2/ST 28:3;O4;T	-	-	$C_{30}H_{49}NO_6SK^+$
779	NAT 28:7	-	-	$C_{30}H_{47}NO_4SK^+$
780	NAT 29:2;O	-	-	$C_{31}H_{59}NO_5SK^+$
781	NAT 29:3;O2/ST 29:0;O4;T	-	-	$C_{31}H_{57}NO_6SK^+$
782	NAT 29:3;O4/ST 29:0;O6;T	-	-	$C_{31}H_{57}NO_8SK^+$
783	NAT 29:4;O3/ST 29:1;O5;T	-	-	$C_{31}H_{55}NO_7SK^+$
784	NAT 29:4;O4/ST 29:1;O6;T	-	-	$C_{31}H_{55}NO_8SK^+$
785	NAT 29:5	-	-	$C_{31}H_{53}NO_4SK^+$
786	NAT 29:5;O2/ST 29:2;O4;T	-	-	$C_{31}H_{53}NO_6SK^+$
787	NAT 29:5;O3/ST 29:2;O5;T	-	-	$C_{31}H_{53}NO_7SK^+$
788	NAT 29:5;O4/ST 29:2;O6;T	-	-	$C_{31}H_{53}NO_8SK^+$
789	NAT 29:6;O3/ST 29:3;O5;T	$C_{31}H_{52}NO_7S^+$	-	-
790	NAT 30:5;O2/ST 30:2;O4;T	-	-	$C_{32}H_{55}NO_6SK^+$
791	NAT 30:6;O2/ST 30:3;O4;T	-	-	$C_{32}H_{53}NO_6SK^+$
792	NAT 30:6;O4/ST 30:3;O6;T	-	-	$C_{32}H_{53}NO_8SK^+$
793	NAT 30:7;O/ST 30:4;O3;T	-	-	$C_{32}H_{51}NO_5SK^+$
794	NAT 30:7;O2/ST 30:4;O4;T	-	-	$C_{32}H_{51}NO_6SK^+$
795	NAT 30:7;O3/ST 30:4;O5;T	-	-	$C_{32}H_{51}NO_7SK^+$
796	NAT 31:3;O3	-	-	$C_{33}H_{61}NO_7SK^+$
797	NAT 31:5;O	-	-	$C_{33}H_{57}NO_5SK^+$
798	NAT 31:6;O2	-	-	$C_{33}H_{55}NO_6SK^+$
799	NAT 31:6;O4	-	-	$C_{33}H_{55}NO_8SK^+$
800	NAT 31:7;O2	-	-	$C_{33}H_{53}NO_6SK^+$
801	NAT 31:7;O3	-	-	$C_{33}H_{53}NO_7SK^+$

802	NAT 31:7;O4	$C_{33}H_{54}NO_8S^+$	-	-
803	NAT 32:4;O2	-	-	$C_{34}H_{61}NO_6SK^+$
804	NAT 32:6	-	-	$C_{34}H_{57}NO_4SK^+$
805	NAT 32:7;O	-	-	$C_{34}H_{55}NO_5SK^+$
806	NAT 32:7;O4	$C_{34}H_{56}NO_8S^+$	-	-
807	NAT 33:6	-	-	$C_{35}H_{59}NO_4SK^+$
808	NAT 33:6;O	-	-	$C_{35}H_{59}NO_5SK^+$
809	NAT 33:7;O2	-	-	$C_{35}H_{57}NO_6SK^+$
810	NAT 34:4;O2	-	-	$C_{36}H_{65}NO_6SK^+$
811	NAT 34:7;O2	-	-	$C_{36}H_{59}NO_6SK^+$
812	PA 20:5	-	$C_{23}H_{35}O_8PNa^+$	-
813	PA 22:6;O	-	$C_{25}H_{37}O_9PNa^+$	-
814	PA 35:9;O	$C_{38}H_{58}O_9P^+$	-	-
815	PA 36:10	$C_{39}H_{58}O_8P^+$	-	-
816	PA 36:8/PA O-36:9;O	-	-	$C_{39}H_{61}O_8PK^+$
817	PA 38:10	-	-	$C_{41}H_{61}O_8PK^+$
818	PA 39:10/PA O-39:11;O	$C_{42}H_{64}O_8P^+$	-	-
819	PA 40:6;O/PG O-37:7	-	$C_{43}H_{73}O_9PNa^+$	-
820	PA 40:7/PA O-40:8;O	-	-	$C_{43}H_{71}O_8PK^+$
821	PA 41:10;O	-	-	$C_{44}H_{67}O_9PK^+$
822	PA 42:8/PA O-42:9;O	-	-	$C_{45}H_{73}O_8PK^+$
823	PA 42:9;O/PG O-39:10	$C_{45}H_{72}O_9P^+$	-	-
824	PA 43:11;O	-	$C_{46}H_{69}O_9PNa^+$	-
825	PA 45:12	-	-	$C_{48}H_{71}O_8PK^+$
826	PA O-31:8	-	-	$C_{34}H_{53}O_7PK^+$
827	PA O-33:8	-	-	$C_{36}H_{57}O_7PK^+$
828	PA O-34:8	-	-	$C_{37}H_{59}O_7PK^+$
829	PA O-35:10	$C_{38}H_{58}O_7P^+$	-	-
830	PA O-35:7	-	-	$C_{38}H_{63}O_7PK^+$
831	PA O-36:10	-	-	$C_{39}H_{59}O_7PK^+$
832	PA O-36:3	-	-	$C_{39}H_{73}O_7PK^+$
833	PA O-36:9	-	-	$C_{39}H_{61}O_7PK^+$
834	PA O-37:8	-	-	$C_{40}H_{65}O_7PK^+$
835	PA O-39:10	$C_{42}H_{66}O_7P^+$	-	$C_{42}H_{65}O_7PK^+$
836	PA O-39:4	-	-	$C_{42}H_{77}O_7PK^+$
837	PA O-42:11	-	-	$C_{45}H_{69}O_7PK^+$
838	PA O-43:12	-	-	$C_{46}H_{69}O_7PK^+$
839	PA O-45:12	-	-	$C_{48}H_{73}O_7PK^+$
840	PC 29:7/PC O-29:8;O/PE 32:7/PE O-32:8;O	-	$C_{37}H_{60}NO_8PNa^+$	-
841	PC 32:9/PE 35:9/PE O-35:10;O	-	-	$C_{40}H_{62}NO_8PK^+$
842	PC 35:9/PC O-35:10;O/PE 38:9/PE O-38:10;O	-	-	$C_{43}H_{68}NO_8PK^+$
843	PC 36:6;O/PE 39:6;O/PS O-38:6	-	$C_{44}H_{76}NO_9PNa^+$	-
844	PC 36:7;O/PE 39:7;O/PS O-38:7	-	$C_{44}H_{74}NO_9PNa^+$	-
845	PC 38:9;O/PE 41:9;O/PS O-40:9	-	$C_{46}H_{74}NO_9PNa^+$	-
846	PC O-32:8/PE O-35:8	-	$C_{40}H_{66}NO_7PNa^+$	-
847	PC O-32:9/PE O-35:9	-	-	$C_{40}H_{64}NO_7PK^+$
848	PC O-34:8/PE O-37:8	-	$C_{42}H_{70}NO_7PNa^+$	$C_{42}H_{70}NO_7PK^+$
849	PC O-35:8/PE O-38:8	-	$C_{43}H_{72}NO_7PNa^+$	-

850	PC O-36:10/PE O-39:10	-	-	$C_{44}H_{70}NO_7PK^+$
851	PE 32:9	$C_{37}H_{57}NO_8P^+$	-	-
852	PE 33:9	-	-	$C_{38}H_{58}NO_8PK^+$
853	PE 34:9	$C_{39}H_{61}NO_8P^+$	-	-
854	PE 35:10;O	$C_{40}H_{61}NO_9P^+$	-	-
855	PE 36:10	$C_{41}H_{63}NO_8P^+$	-	-
856	PE 37:10;O/PS O-36:10	-	-	$C_{42}H_{64}NO_9PK^+$
857	PE O-30:8	-	-	$C_{35}H_{56}NO_7PK^+$
858	PE O-37:10	$C_{42}H_{67}NO_7P^+$	-	-
859	PE O-39:11	-	$C_{44}H_{68}NO_7PNa^+$	-
860	PE-Cer 28:3;O5	-	$C_{30}H_{57}N_2O_9PNa^+$	-
861	PE-Cer 29:0;O6	-	$C_{31}H_{65}N_2O_{10}PNa^+$	-
862	PE-Cer 29:1;O6	-	$C_{31}H_{63}N_2O_{10}PNa^+$	-
863	PE-Cer 30:2;O6	$C_{32}H_{64}N_2O_{10}P^+$	-	-
864	PE-Cer 30:3;O6	-	$C_{32}H_{61}N_2O_{10}PNa^+$	-
865	PE-Cer 31:1;O6/SM 28:1;O6	-	$C_{33}H_{67}N_2O_{10}PNa^+$	-
866	PE-Cer 33:6;O4/SM 30:6;O4	$C_{35}H_{62}N_2O_8P^+$	-	-
867	PE-Cer 35:0;O2/SM 32:0;O2	-	-	$C_{37}H_{77}N_2O_6PK^+$
868	PE-Cer 35:6;O4/SM 32:6;O4	$C_{37}H_{66}N_2O_8P^+$	-	-
869	PG 35:1/PG O-35:2;O	-	$C_{41}H_{79}O_{10}PNa^+$	-
870	PG 35:2/PG O-35:3;O	-	$C_{41}H_{77}O_{10}PNa^+$	-
871	PI 31:7;O	-	$C_{40}H_{63}O_{14}PNa^+$	-
872	PI-Cer 28:1;O4	-	$C_{34}H_{66}NO_{13}PNa^+$	-
873	PI-Cer 28:2;O4	-	$C_{34}H_{64}NO_{13}PNa^+$	-
874	PI-Cer 31:1;O2/PS 31:0;O	$C_{37}H_{73}NO_{11}P^+$	-	-
875	PI-Cer 32:0;O4	$C_{38}H_{77}NO_{13}P^+$	-	-
876	PI-Cer 33:0;O4	-	-	$C_{39}H_{78}NO_{13}PK^+$
877	PI-Cer 34:3;O6	$C_{40}H_{75}NO_{15}P^+$	-	-
878	PI-Cer 35:2;O2/PS 35:1;O	-	-	$C_{41}H_{78}NO_{11}PK^+$
879	PI-Cer 36:5;O3	-	$C_{42}H_{74}NO_{12}PNa^+$	-
880	PI-Cer 48:4;O6	-	$C_{54}H_{100}NO_{15}PNa^+$	-
881	PIP 26:1;O	$C_{35}H_{67}O_{17}P_2^+$	-	-
882	PIP 33:9;O	-	$C_{42}H_{64}O_{17}P_2Na^+$	-
883	PIP2 26:0;O	-	$C_{35}H_{69}O_{20}P_3Na^+$	-
884	PIP2 28:3;O	$C_{37}H_{68}O_{20}P_3^+$	-	-
885	PS 22:0;O	-	$C_{28}H_{54}NO_{11}PNa^+$	-
886	PS 22:1;O	-	$C_{28}H_{52}NO_{11}PNa^+$	-
887	PS 23:1;O	-	$C_{29}H_{54}NO_{11}PNa^+$	-
888	PS 24:6	-	-	$C_{30}H_{46}NO_{10}PK^+$
889	PS 24:6;O	-	-	$C_{30}H_{46}NO_{11}PK^+$
890	PS 38:6/PS O-38:7;O	-	$C_{44}H_{74}NO_{10}PNa^+$	-
891	PS 40:9/PS O-40:10;O	$C_{46}H_{73}NO_{10}P^+$	-	-
892	SHexCer 29:1;O3	$C_{35}H_{68}NO_{12}S^+$	-	-
893	SHexCer 29:4;O4	-	-	$C_{35}H_{61}NO_{13}SK^+$
894	SHexCer 31:0;O3	$C_{37}H_{74}NO_{12}S^+$	$C_{37}H_{73}NO_{12}SNa^+$	-
895	SHexCer 31:1;O3	$C_{37}H_{72}NO_{12}S^+$	$C_{37}H_{71}NO_{12}SNa^+$	-
896	SHexCer 32:1;O3	$C_{38}H_{74}NO_{12}S^+$	-	-
897	SHexCer 32:3;O4	-	$C_{38}H_{69}NO_{13}SNa^+$	-

898	SHexCer 33:0;O5	$C_{39}H_{78}NO_{14}S^+$	$C_{39}H_{77}NO_{14}SNa^+$	$C_{39}H_{77}NO_{14}SK^+$
899	SHexCer 33:1;O5	$C_{39}H_{76}NO_{14}S^+$	-	-
900	SHexCer 33:3;O3	$C_{39}H_{72}NO_{12}S^+$	-	-
901	SHexCer 33:4;O3	$C_{39}H_{70}NO_{12}S^+$	-	-
902	SHexCer 34:3;O4	-	$C_{40}H_{73}NO_{13}SNa^+$	-
903	SHexCer 34:4;O4	-	$C_{40}H_{71}NO_{13}SNa^+$	-
904	SHexCer 34:6;O2	$C_{40}H_{68}NO_{11}S^+$	-	-
905	SHexCer 35:3;O4	-	-	$C_{41}H_{75}NO_{13}SK^+$
906	SHexCer 35:3;O5	$C_{41}H_{76}NO_{14}S^+$	$C_{41}H_{75}NO_{14}SNa^+$	-
907	SHexCer 36:4;O3	-	$C_{42}H_{75}NO_{12}SNa^+$	$C_{42}H_{75}NO_{12}SK^+$
908	SHexCer 36:4;O4	-	$C_{42}H_{75}NO_{13}SNa^+$	-
909	SHexCer 36:5;O4	-	$C_{42}H_{73}NO_{13}SNa^+$	-
910	SHexCer 45:0;O2	$C_{51}H_{102}NO_{11}S^+$	-	-
911	SPB 14:1;O	$C_{14}H_{30}NO^+$	-	-
912	SPB 14:2;O	$C_{14}H_{28}NO^+$	-	-
913	SPB 14:3;O	$C_{14}H_{26}NO^+$	-	-
914	SPB 15:1;O	$C_{15}H_{32}NO^+$	-	-
915	SPB 15:2;O	$C_{15}H_{30}NO^+$	-	-
916	SPB 15:3;O	$C_{15}H_{28}NO^+$	-	-
917	SPB 16:1;O	$C_{16}H_{34}NO^+$	-	-
918	SPB 16:2;O	$C_{16}H_{32}NO^+$	-	-
919	SPB 16:3;O	$C_{16}H_{30}NO^+$	-	-
920	SPB 17:1;O	$C_{17}H_{36}NO^+$	-	-
921	SPB 17:2;O	$C_{17}H_{34}NO^+$	-	-
922	SPB 17:3;O	$C_{17}H_{32}NO^+$	-	-
923	SPB 18:1;O	$C_{18}H_{38}NO^+$	-	-
924	SPB 18:2;O	$C_{18}H_{36}NO^+$	-	-
925	SPB 18:3;O	$C_{18}H_{34}NO^+$	-	-
926	SPB 19:1;O	$C_{19}H_{40}NO^+$	-	-
927	SPB 19:2;O	$C_{19}H_{38}NO^+$	-	-
928	SPB 19:3;O	$C_{19}H_{36}NO^+$	-	-
929	SPB 20:3;O	$C_{20}H_{38}NO^+$	-	-
930	SPBP 14:0;O4	-	$C_{14}H_{32}NO_7PNa^+$	-
931	SPBP 14:1;O3	-	$C_{14}H_{30}NO_6PNa^+$	-
932	SPBP 14:1;O4	-	$C_{14}H_{30}NO_7PNa^+$	-
933	SPBP 15:2;O2	$C_{15}H_{31}NO_5P^+$	-	-
934	SPBP 17:1;O2	-	-	$C_{17}H_{36}NO_5PK^+$
935	SPBP 18:1;O2	$C_{18}H_{39}NO_5P^+$	-	-
936	ST 18:0;O2;GlCA/ST 18:1;O3;Hex/ST 24:2;O8	-	$C_{24}H_{38}O_8Na^+$	-
937	ST 18:0;O3;GlCA/ST 18:1;O4;Hex	-	$C_{24}H_{38}O_9Na^+$	-
938	ST 18:0;O3;Hex/ST 24:1;O8	-	$C_{24}H_{40}O_8Na^+$	-
939	ST 18:0;O4;Hex	-	$C_{24}H_{40}O_9Na^+$	-
940	ST 18:0;O4;HexNAC/ST 24:1;O8;G	-	$C_{26}H_{43}NO_9Na^+$	-
941	ST 18:0;O5;GlCA/ST 18:1;O6;Hex	-	$C_{24}H_{38}O_{11}Na^+$	-
942	ST 18:0;O5;Hex	-	$C_{24}H_{40}O_{10}Na^+$	-
943	ST 18:0;O5;HexNAC	-	$C_{26}H_{43}NO_{10}Na^+$	-
944	ST 18:0;O6;GlCA/ST 18:1;O7;Hex	-	$C_{24}H_{38}O_{12}Na^+$	-
945	ST 18:0;O6;HexNAC	-	$C_{26}H_{43}NO_{11}Na^+$	-

946	ST 18:0;O7	-	$C_{18}H_{30}O_7Na^+$	-
947	ST 18:0;O7;GlCA/ST 18:1;O8;Hex	-	$C_{24}H_{38}O_{13}Na^+$	-
948	ST 18:0;O7;Hex	$C_{24}H_{41}O_{12}^+$	$C_{24}H_{40}O_{12}Na^+$	-
949	ST 18:0;O7;HexNAC	-	$C_{26}H_{43}NO_{12}Na^+$	-
950	ST 18:0;O8	-	$C_{18}H_{30}O_8Na^+$	-
951	ST 18:0;O8;G	-	$C_{20}H_{33}NO_9Na^+$	-
952	ST 18:0;O8;GlCA	$C_{24}H_{39}O_{14}^+$	-	-
953	ST 18:0;O8;HexNAC	$C_{26}H_{44}NO_{13}^+$	-	-
954	ST 18:1;O3;GlCA/ST 18:2;O4;Hex	-	$C_{24}H_{36}O_9Na^+$	-
955	ST 18:1;O3;S	-	-	$C_{18}H_{28}NO_6SK^+$
956	ST 18:1;O4;GlCA/ST 18:2;O5;Hex	-	$C_{24}H_{36}O_{10}Na^+$	-
957	ST 18:1;O4;HexNAC/ST 24:2;O8;G	-	$C_{26}H_{41}NO_9Na^+$	-
958	ST 18:1;O5;HexNAC	-	$C_{26}H_{41}NO_{10}Na^+$	-
959	ST 18:1;O6;G	-	$C_{20}H_{31}NO_7Na^+$	-
960	ST 18:1;O6;GlCA/ST 18:2;O7;Hex	-	$C_{24}H_{36}O_{12}Na^+$	-
961	ST 18:1;O6;HexNAC	-	$C_{26}H_{41}NO_{11}Na^+$	-
962	ST 18:1;O7;G	-	$C_{20}H_{31}NO_8Na^+$	-
963	ST 18:1;O7;HexNAC	-	$C_{26}H_{41}NO_{12}Na^+$	-
964	ST 18:1;O8;G	-	$C_{20}H_{31}NO_9Na^+$	-
965	ST 18:2;O;S	-	-	$C_{18}H_{26}NO_4SK^+$
966	ST 18:2;O3;HexNAC/ST 24:3;O7;G	-	$C_{26}H_{39}NO_8Na^+$	-
967	ST 18:2;O3;S	-	-	$C_{18}H_{26}NO_6SK^+$
968	ST 18:2;O5;GlCA/ST 18:3;O6;Hex	$C_{24}H_{35}O_{11}^+$	-	-
969	ST 18:2;O6;HexNAC	-	$C_{26}H_{39}NO_{11}Na^+$	-
970	ST 18:2;O8;G	-	$C_{20}H_{29}NO_9Na^+$	-
971	ST 18:2;O8;HexNAC	$C_{26}H_{40}NO_{13}^+$	-	-
972	ST 18:3;O5;GlCA/ST 18:4;O6;Hex	$C_{24}H_{33}O_{11}^+$	-	-
973	ST 18:3;O7;HexNAC	-	$C_{26}H_{37}NO_{12}Na^+$	-
974	ST 18:3;O8;HexNAC	$C_{26}H_{38}NO_{13}^+$	-	-
975	ST 18:4;O5;GlCA/ST 18:5;O6;Hex	$C_{24}H_{31}O_{11}^+$	-	-
976	ST 18:4;O8;GlCA	$C_{24}H_{31}O_{14}^+$	-	-
977	ST 19:0;O3;GlCA/ST 19:1;O4;Hex	-	$C_{25}H_{40}O_9Na^+$	-
978	ST 19:0;O3;Hex/ST 25:1;O8	-	$C_{25}H_{42}O_8Na^+$	-
979	ST 19:0;O4;GlCA/ST 19:1;O5;Hex	-	$C_{25}H_{40}O_{10}Na^+$	-
980	ST 19:0;O4;Hex	-	$C_{25}H_{42}O_9Na^+$	-
981	ST 19:0;O4;HexNAC/ST 25:1;O8;G	-	$C_{27}H_{45}NO_9Na^+$	-
982	ST 19:0;O5;GlCA/ST 19:1;O6;Hex	-	$C_{25}H_{40}O_{11}Na^+$	-
983	ST 19:0;O5;Hex	-	$C_{25}H_{42}O_{10}Na^+$	-
984	ST 19:0;O5;HexNAC	-	$C_{27}H_{45}NO_{10}Na^+$	-
985	ST 19:0;O6;GlCA/ST 19:1;O7;Hex	-	$C_{25}H_{40}O_{12}Na^+$	-
986	ST 19:0;O6;HexNAC	-	$C_{27}H_{45}NO_{11}Na^+$	-
987	ST 19:0;O7	-	$C_{19}H_{32}O_7Na^+$	-
988	ST 19:0;O7;GlCA/ST 19:1;O8;Hex	$C_{25}H_{41}O_{13}^+$	$C_{25}H_{40}O_{13}Na^+$	-
989	ST 19:0;O7;Hex	-	$C_{25}H_{42}O_{12}Na^+$	-
990	ST 19:0;O7;HexNAC	$C_{27}H_{46}NO_{12}^+$	-	-
991	ST 19:0;O8	-	$C_{19}H_{32}O_8Na^+$	-
992	ST 19:0;O8;G	-	$C_{21}H_{35}NO_9Na^+$	-
993	ST 19:0;O8;GlCA	$C_{25}H_{41}O_{14}^+$	-	-

994	ST 19:0;O8;Hex	$C_{25}H_{43}O_{13}^+$	$C_{25}H_{42}O_{13}Na^+$	-
995	ST 19:1;O3;GlCA/ST 19:2;O4;Hex	-	$C_{25}H_{38}O_9Na^+$	-
996	ST 19:1;O4;GlCA/ST 19:2;O5;Hex	-	$C_{25}H_{38}O_{10}Na^+$	-
997	ST 19:1;O5;GlCA/ST 19:2;O6;Hex	-	$C_{25}H_{38}O_{11}Na^+$	-
998	ST 19:1;O5;HexNAC	-	$C_{27}H_{43}NO_{10}Na^+$	-
999	ST 19:1;O6;HexNAC	-	$C_{27}H_{43}NO_{11}Na^+$	-
1000	ST 19:1;O7	-	$C_{19}H_{30}O_7Na^+$	-
1001	ST 19:1;O7;HexNAC	-	$C_{27}H_{43}NO_{12}Na^+$	-
1002	ST 19:1;O8	-	$C_{19}H_{30}O_8Na^+$	-
1003	ST 19:1;O8;G	-	$C_{21}H_{33}NO_9Na^+$	-
1004	ST 19:2;O3;GlCA/ST 19:3;O4;Hex	-	$C_{25}H_{36}O_9Na^+$	-
1005	ST 19:2;O4;GlCA/ST 19:3;O5;Hex	-	$C_{25}H_{36}O_{10}Na^+$	-
1006	ST 19:2;O5;GlCA/ST 19:3;O6;Hex	$C_{25}H_{37}O_{11}^+$	-	-
1007	ST 19:2;O6;GlCA/ST 19:3;O7;Hex	-	$C_{25}H_{36}O_{12}Na^+$	-
1008	ST 19:2;O7;G	-	$C_{21}H_{31}NO_8Na^+$	-
1009	ST 19:2;O8;HexNAC	-	$C_{27}H_{41}NO_{13}Na^+$	-
1010	ST 19:3;O;S	-	-	$C_{19}H_{26}NO_4SK^+$
1011	ST 19:3;O2;S	-	-	$C_{19}H_{26}NO_5SK^+$
1012	ST 19:3;O7;HexNAC	-	$C_{27}H_{39}NO_{12}Na^+$	-
1013	ST 19:5;O;S	-	$C_{19}H_{22}NO_4SNa^+$	-
1014	ST 19:5;O3	-	$C_{19}H_{22}O_3Na^+$	-
1015	ST 19:5;O7;HexNAC	$C_{27}H_{36}NO_{12}^+$	-	-
1016	ST 20:0;O3;Hex/ST 26:1;O8	-	$C_{26}H_{44}O_8Na^+$	-
1017	ST 20:0;O4;GlCA/ST 20:1;O5;Hex	-	$C_{26}H_{42}O_{10}Na^+$	-
1018	ST 20:0;O4;Hex	-	$C_{26}H_{44}O_9Na^+$	-
1019	ST 20:0;O4;HexNAC/ST 26:1;O8;G	-	$C_{28}H_{47}NO_9Na^+$	-
1020	ST 20:0;O5;GlCA/ST 20:1;O6;Hex	-	$C_{26}H_{42}O_{11}Na^+$	-
1021	ST 20:0;O5;Hex	-	$C_{26}H_{44}O_{10}Na^+$	-
1022	ST 20:0;O5;HexNAC	-	$C_{28}H_{47}NO_{10}Na^+$	-
1023	ST 20:0;O6;GlCA/ST 20:1;O7;Hex	$C_{26}H_{43}O_{12}^+$	$C_{26}H_{42}O_{12}Na^+$	-
1024	ST 20:0;O6;Hex	-	$C_{26}H_{44}O_{11}Na^+$	-
1025	ST 20:0;O6;HexNAC	-	$C_{28}H_{47}NO_{11}Na^+$	-
1026	ST 20:0;O7	-	$C_{20}H_{34}O_7Na^+$	-
1027	ST 20:0;O7;GlCA/ST 20:1;O8;Hex	-	$C_{26}H_{42}O_{13}Na^+$	-
1028	ST 20:0;O7;Hex	-	$C_{26}H_{44}O_{12}Na^+$	-
1029	ST 20:0;O7;HexNAC	$C_{28}H_{48}NO_{12}^+$	-	-
1030	ST 20:0;O8	-	$C_{20}H_{34}O_8Na^+$	-
1031	ST 20:0;O8;G	-	$C_{22}H_{37}NO_9Na^+$	-
1032	ST 20:0;O8;GlCA	$C_{26}H_{43}O_{14}^+$	$C_{26}H_{42}O_{14}Na^+$	-
1033	ST 20:0;O8;Hex	-	$C_{26}H_{44}O_{13}Na^+$	-
1034	ST 20:0;O8;HexNAC	$C_{28}H_{48}NO_{13}^+$	-	-
1035	ST 20:1;O5;GlCA/ST 20:2;O6;Hex	-	$C_{26}H_{40}O_{11}Na^+$	-
1036	ST 20:1;O6;HexNAC	-	$C_{28}H_{45}NO_{11}Na^+$	-
1037	ST 20:1;O7	-	$C_{20}H_{32}O_7Na^+$	-
1038	ST 20:1;O8	-	$C_{20}H_{32}O_8Na^+$	-
1039	ST 20:1;O8;G	-	$C_{22}H_{35}NO_9Na^+$	-
1040	ST 20:1;O8;GlCA	$C_{26}H_{41}O_{14}^+$	-	-
1041	ST 20:2;O;S	-	-	$C_{20}H_{30}NO_4SK^+$

1042	ST 20:2;O5;GlCA/ST 20:3;O6;Hex	$C_{26}H_{39}O_{11}^{+}$	-	-
1043	ST 20:2;O7;G	-	$C_{22}H_{33}NO_{8}Na^{+}$	-
1044	ST 20:2;O8;G	-	$C_{22}H_{33}NO_{9}Na^{+}$	-
1045	ST 20:3;O;S	-	-	$C_{20}H_{28}NO_{4}SK^{+}$
1046	ST 20:3;O3;S	-	-	$C_{20}H_{28}NO_{6}SK^{+}$
1047	ST 20:3;O3;T	-	$C_{22}H_{33}NO_{5}SNa^{+}$	$C_{22}H_{33}NO_{5}SK^{+}$
1048	ST 20:3;O4;HexNAC/ST 26:4;O8;G	-	$C_{28}H_{41}NO_{9}Na^{+}$	-
1049	ST 20:3;O8;G	-	$C_{22}H_{31}NO_{9}Na^{+}$	-
1050	ST 20:4;O2;S	-	$C_{20}H_{26}NO_{5}SNa^{+}$	-
1051	ST 20:4;O4;S	-	-	$C_{20}H_{26}NO_{7}SK^{+}$
1052	ST 20:4;O5;GlCA/ST 20:5;O6;Hex	-	$C_{26}H_{34}O_{11}Na^{+}$	-
1053	ST 20:5;O8	$C_{20}H_{25}O_{8}^{+}$	-	-
1054	ST 21:0;O2;GlCA/ST 21:1;O3;Hex/ST 27:2;O8	-	$C_{27}H_{44}O_{8}Na^{+}$	-
1055	ST 21:0;O3;GlCA/ST 21:1;O4;Hex	-	$C_{27}H_{44}O_{9}Na^{+}$	-
1056	ST 21:0;O3;Hex/ST 27:1;O8	-	$C_{27}H_{46}O_{8}Na^{+}$	-
1057	ST 21:0;O4;GlCA/ST 21:1;O5;Hex	-	$C_{27}H_{44}O_{10}Na^{+}$	-
1058	ST 21:0;O4;Hex	-	$C_{27}H_{46}O_{9}Na^{+}$	-
1059	ST 21:0;O4;HexNAC/ST 27:1;O8;G	-	$C_{29}H_{49}NO_{9}Na^{+}$	-
1060	ST 21:0;O5;GlCA/ST 21:1;O6;Hex	$C_{27}H_{45}O_{11}^{+}$	-	-
1061	ST 21:0;O5;Hex	-	$C_{27}H_{46}O_{10}Na^{+}$	-
1062	ST 21:0;O5;HexNAC	-	$C_{29}H_{49}NO_{10}Na^{+}$	-
1063	ST 21:0;O6;GlCA/ST 21:1;O7;Hex	-	$C_{27}H_{44}O_{12}Na^{+}$	-
1064	ST 21:0;O6;Hex	$C_{27}H_{47}O_{11}^{+}$	-	-
1065	ST 21:0;O7	-	$C_{21}H_{36}O_{7}Na^{+}$	-
1066	ST 21:0;O7;Hex	$C_{27}H_{47}O_{12}^{+}$	$C_{27}H_{46}O_{12}Na^{+}$	-
1067	ST 21:0;O7;HexNAC	-	$C_{29}H_{49}NO_{12}Na^{+}$	-
1068	ST 21:0;O8	-	$C_{21}H_{36}O_{8}Na^{+}$	-
1069	ST 21:0;O8;G	-	$C_{23}H_{39}NO_{9}Na^{+}$	-
1070	ST 21:0;O8;GlCA	$C_{27}H_{45}O_{14}^{+}$	$C_{27}H_{44}O_{14}Na^{+}$	-
1071	ST 21:0;O8;HexNAC	$C_{29}H_{50}NO_{13}^{+}$	-	-
1072	ST 21:1;O3;S	-	-	$C_{21}H_{34}NO_{6}SK^{+}$
1073	ST 21:1;O4;GlCA/ST 21:2;O5;Hex	-	$C_{27}H_{42}O_{10}Na^{+}$	-
1074	ST 21:1;O4;HexNAC/ST 27:2;O8;G	-	$C_{29}H_{47}NO_{9}Na^{+}$	-
1075	ST 21:1;O5;GlCA/ST 21:2;O6;Hex	$C_{27}H_{43}O_{11}^{+}$	$C_{27}H_{42}O_{11}Na^{+}$	-
1076	ST 21:1;O5;HexNAC	-	$C_{29}H_{47}NO_{10}Na^{+}$	-
1077	ST 21:1;O6;GlCA/ST 21:2;O7;Hex	$C_{27}H_{43}O_{12}^{+}$	$C_{27}H_{42}O_{12}Na^{+}$	-
1078	ST 21:1;O6;HexNAC	-	$C_{29}H_{47}NO_{11}Na^{+}$	-
1079	ST 21:1;O7	-	$C_{21}H_{34}O_{7}Na^{+}$	-
1080	ST 21:1;O7;S	$C_{21}H_{35}NO_{10}S^{+}$	-	-
1081	ST 21:1;O8	-	$C_{21}H_{34}O_{8}Na^{+}$	-
1082	ST 21:1;O8;G	-	$C_{23}H_{37}NO_{9}Na^{+}$	-
1083	ST 21:2;O;S	-	-	$C_{21}H_{32}NO_{4}SK^{+}$
1084	ST 21:2;O2;S	-	-	$C_{21}H_{32}NO_{5}SK^{+}$
1085	ST 21:2;O4;GlCA/ST 21:3;O5;Hex	-	$C_{27}H_{40}O_{10}Na^{+}$	-
1086	ST 21:2;O5;GlCA/ST 21:3;O6;Hex	$C_{27}H_{41}O_{11}^{+}$	-	-
1087	ST 21:2;O6;GlCA/ST 21:3;O7;Hex	-	$C_{27}H_{40}O_{12}Na^{+}$	-
1088	ST 21:2;O6;HexNAC	-	$C_{29}H_{45}NO_{11}Na^{+}$	-
1089	ST 21:2;O7	-	$C_{21}H_{32}O_{7}Na^{+}$	-

1090	ST 21:2;O7;G	-	$C_{23}H_{35}NO_{8}Na^{+}$	-
1091	ST 21:2;O8;G	-	$C_{23}H_{35}NO_{9}Na^{+}$	-
1092	ST 21:3;O	-	$C_{21}H_{30}O_{1}Na^{+}$	-
1093	ST 21:3;O5;GlCA/ST 21:4;O6;Hex	$C_{27}H_{39}O_{11}^{+}$	-	-
1094	ST 21:4;O3;T	-	-	$C_{23}H_{33}NO_{5}SK^{+}$
1095	ST 21:4;O4;G	-	$C_{23}H_{31}NO_{5}Na^{+}$	-
1096	ST 21:5;O8;GlCA	-	$C_{27}H_{34}O_{14}Na^{+}$	-
1097	ST 21:6;O;S	-	-	$C_{21}H_{24}NO_{4}SK^{+}$
1098	ST 22:0;O;S	-	-	$C_{22}H_{38}NO_{4}SK^{+}$
1099	ST 22:0;O2;S	-	-	$C_{22}H_{38}NO_{5}SK^{+}$
1100	ST 22:0;O3;Hex/ST 28:1;O8	-	$C_{28}H_{48}O_{8}Na^{+}$	-
1101	ST 22:0;O4;GlCA/ST 22:1;O5;Hex	-	$C_{28}H_{46}O_{10}Na^{+}$	-
1102	ST 22:0;O4;Hex	-	$C_{28}H_{48}O_{9}Na^{+}$	-
1103	ST 22:0;O4;HexNAC/ST 28:1;O8;G	-	$C_{30}H_{51}NO_{9}Na^{+}$	-
1104	ST 22:0;O5;GlCA/ST 22:1;O6;Hex	-	$C_{28}H_{46}O_{11}Na^{+}$	-
1105	ST 22:0;O5;Hex	-	$C_{28}H_{48}O_{10}Na^{+}$	-
1106	ST 22:0;O6;GlCA/ST 22:1;O7;Hex	-	$C_{28}H_{46}O_{12}Na^{+}$	-
1107	ST 22:0;O6;Hex	$C_{28}H_{49}O_{11}^{+}$	$C_{28}H_{48}O_{11}Na^{+}$	-
1108	ST 22:0;O6;HexNAC	-	$C_{30}H_{51}NO_{11}Na^{+}$	-
1109	ST 22:0;O7	-	$C_{22}H_{38}O_{7}Na^{+}$	-
1110	ST 22:0;O7;GlCA/ST 22:1;O8;Hex	-	$C_{28}H_{46}O_{13}Na^{+}$	-
1111	ST 22:0;O7;HexNAC	-	$C_{30}H_{51}NO_{12}Na^{+}$	-
1112	ST 22:0;O8	-	$C_{22}H_{38}O_{8}Na^{+}$	-
1113	ST 22:0;O8;G	-	$C_{24}H_{41}NO_{9}Na^{+}$	-
1114	ST 22:0;O8;GlCA	$C_{28}H_{47}O_{14}^{+}$	-	-
1115	ST 22:0;O8;Hex	$C_{28}H_{49}O_{13}^{+}$	$C_{28}H_{48}O_{13}Na^{+}$	-
1116	ST 22:1;O;S	-	-	$C_{22}H_{36}NO_{4}SK^{+}$
1117	ST 22:1;O4;GlCA/ST 22:2;O5;Hex	-	$C_{28}H_{44}O_{10}Na^{+}$	-
1118	ST 22:1;O4;HexNAC/ST 28:2;O8;G	-	$C_{30}H_{49}NO_{9}Na^{+}$	-
1119	ST 22:1;O5;GlCA/ST 22:2;O6;Hex	$C_{28}H_{45}O_{11}^{+}$	$C_{28}H_{44}O_{11}Na^{+}$	-
1120	ST 22:1;O6;GlCA/ST 22:2;O7;Hex	$C_{28}H_{45}O_{12}^{+}$	-	-
1121	ST 22:1;O6;HexNAC	-	$C_{30}H_{49}NO_{11}Na^{+}$	-
1122	ST 22:1;O7	-	$C_{22}H_{36}O_{7}Na^{+}$	-
1123	ST 22:1;O7;GlCA/ST 22:2;O8;Hex	-	$C_{28}H_{44}O_{13}Na^{+}$	-
1124	ST 22:1;O7;HexNAC	-	$C_{30}H_{49}NO_{12}Na^{+}$	-
1125	ST 22:1;O8	-	$C_{22}H_{36}O_{8}Na^{+}$	-
1126	ST 22:1;O8;G	-	$C_{24}H_{39}NO_{9}Na^{+}$	-
1127	ST 22:1;O8;HexNAC	$C_{30}H_{50}NO_{13}^{+}$	-	-
1128	ST 22:2;O2;S	-	-	$C_{22}H_{34}NO_{5}SK^{+}$
1129	ST 22:2;O3;S	-	-	$C_{22}H_{34}NO_{6}SK^{+}$
1130	ST 22:2;O4;GlCA/ST 22:3;O5;Hex	-	$C_{28}H_{42}O_{10}Na^{+}$	-
1131	ST 22:2;O5;GlCA/ST 22:3;O6;Hex	-	$C_{28}H_{42}O_{11}Na^{+}$	-
1132	ST 22:2;O5;S	$C_{22}H_{35}NO_{8}S^{+}$	-	$C_{22}H_{34}NO_{8}SK^{+}$
1133	ST 22:2;O6;G	-	$C_{24}H_{37}NO_{7}Na^{+}$	-
1134	ST 22:2;O6;GlCA/ST 22:3;O7;Hex	-	$C_{28}H_{42}O_{12}Na^{+}$	-
1135	ST 22:2;O6;HexNAC	-	$C_{30}H_{47}NO_{11}Na^{+}$	-
1136	ST 22:2;O6;S	-	-	$C_{22}H_{34}NO_{9}SK^{+}$
1137	ST 22:2;O7;G	-	$C_{24}H_{37}NO_{8}Na^{+}$	-

1138	ST 22:2;O7;HexNAC	-	$C_{30}H_{47}NO_{12}Na^+$	-
1139	ST 22:2;O8	-	$C_{22}H_{34}O_8Na^+$	-
1140	ST 22:2;O8;G	-	$C_{24}H_{37}NO_9Na^+$	-
1141	ST 22:2;O8;HexNAC	$C_{30}H_{48}NO_{13}^+$	-	-
1142	ST 22:3;O;S	-	-	$C_{22}H_{32}NO_4SK^+$
1143	ST 22:3;O2;S	-	-	$C_{22}H_{32}NO_5SK^+$
1144	ST 22:3;O3;S	-	-	$C_{22}H_{32}NO_6SK^+$
1145	ST 22:3;O4;HexNAC/ST 28:4;O8;G	$C_{30}H_{46}NO_9^+$	-	-
1146	ST 22:3;O4;S	-	-	$C_{22}H_{32}NO_7SK^+$
1147	ST 22:3;O5;S	-	-	$C_{22}H_{32}NO_8SK^+$
1148	ST 22:3;O8;G	-	$C_{24}H_{35}NO_9Na^+$	-
1149	ST 22:4;O3;S	-	-	$C_{22}H_{30}NO_6SK^+$
1150	ST 22:4;O4;S	-	-	$C_{22}H_{30}NO_7SK^+$
1151	ST 23:0;O3;GlCA/ST 23:1;O4;Hex	-	$C_{29}H_{48}O_9Na^+$	-
1152	ST 23:0;O5;Hex	-	$C_{29}H_{50}O_{10}Na^+$	-
1153	ST 23:0;O5;HexNAC	-	$C_{31}H_{53}NO_{10}Na^+$	-
1154	ST 23:0;O6;GlCA/ST 23:1;O7;Hex	$C_{29}H_{49}O_{12}^+$	$C_{29}H_{48}O_{12}Na^+$	-
1155	ST 23:0;O6;HexNAC	-	$C_{31}H_{53}NO_{11}Na^+$	-
1156	ST 23:0;O7;GlCA/ST 23:1;O8;Hex	$C_{29}H_{49}O_{13}^+$	$C_{29}H_{48}O_{13}Na^+$	-
1157	ST 23:0;O7;Hex	-	$C_{29}H_{50}O_{12}Na^+$	-
1158	ST 23:0;O8	-	$C_{23}H_{40}O_8Na^+$	-
1159	ST 23:0;O8;G	-	$C_{25}H_{43}NO_9Na^+$	-
1160	ST 23:0;O8;GlCA	$C_{29}H_{49}O_{14}^+$	$C_{29}H_{48}O_{14}Na^+$	-
1161	ST 23:0;O8;Hex	$C_{29}H_{51}O_{13}^+$	$C_{29}H_{50}O_{13}Na^+$	-
1162	ST 23:1;O;S	-	-	$C_{23}H_{38}NO_4SK^+$
1163	ST 23:1;O3;GlCA/ST 23:2;O4;Hex	-	$C_{29}H_{46}O_9Na^+$	-
1164	ST 23:1;O5;GlCA/ST 23:2;O6;Hex	$C_{29}H_{47}O_{11}^+$	$C_{29}H_{46}O_{11}Na^+$	-
1165	ST 23:1;O5;HexNAC	-	$C_{31}H_{51}NO_{10}Na^+$	-
1166	ST 23:1;O6;GlCA/ST 23:2;O7;Hex	$C_{29}H_{47}O_{12}^+$	$C_{29}H_{46}O_{12}Na^+$	-
1167	ST 23:1;O6;HexNAC	-	$C_{31}H_{51}NO_{11}Na^+$	-
1168	ST 23:1;O7;HexNAC	-	$C_{31}H_{51}NO_{12}Na^+$	-
1169	ST 23:1;O8	-	$C_{23}H_{38}O_8Na^+$	-
1170	ST 23:1;O8;G	-	$C_{25}H_{41}NO_9Na^+$	-
1171	ST 23:1;O8;GlCA	-	$C_{29}H_{46}O_{14}Na^+$	-
1172	ST 23:2;O;S	-	-	$C_{23}H_{36}NO_4SK^+$
1173	ST 23:2;O2;GlCA/ST 23:3;O3;Hex/ST 29:4;O8	-	$C_{29}H_{44}O_8Na^+$	-
1174	ST 23:2;O4;S	-	-	$C_{23}H_{36}NO_7SK^+$
1175	ST 23:2;O5;GlCA/ST 23:3;O6;Hex	$C_{29}H_{45}O_{11}^+$	-	-
1176	ST 23:2;O6;GlCA/ST 23:3;O7;Hex	$C_{29}H_{45}O_{12}^+$	$C_{29}H_{44}O_{12}Na^+$	-
1177	ST 23:2;O6;HexNAC	-	$C_{31}H_{49}NO_{11}Na^+$	-
1178	ST 23:2;O8	-	$C_{23}H_{36}O_8Na^+$	-
1179	ST 23:2;O8;G	-	$C_{25}H_{39}NO_9Na^+$	-
1180	ST 23:2;O8;S	$C_{23}H_{37}NO_{11}S^+$	$C_{23}H_{36}NO_{11}SNa^+$	-
1181	ST 23:3;O2;HexNAC/ST 29:4;O6;G	$C_{31}H_{48}NO_7^+$	-	-
1182	ST 23:3;O5;GlCA/ST 23:4;O6;Hex	$C_{29}H_{43}O_{11}^+$	-	-
1183	ST 23:3;O6;S	-	-	$C_{23}H_{34}NO_9SK^+$
1184	ST 23:3;O7;S	$C_{23}H_{35}NO_{10}S^+$	$C_{23}H_{34}NO_{10}SNa^+$	-
1185	ST 23:3;O8	-	$C_{23}H_{34}O_8Na^+$	-

1186	ST 23:3;O8;G	-	$C_{25}H_{37}NO_9Na^+$	-
1187	ST 23:3;O8;GlCA	-	$C_{29}H_{42}O_{14}Na^+$	-
1188	ST 23:4;O8;G	-	$C_{25}H_{35}NO_9Na^+$	-
1189	ST 23:5;O;S	-	-	$C_{23}H_{30}NO_4SK^+$
1190	ST 23:6;O;S	$C_{23}H_{29}NO_4S^+$	-	-
1191	ST 23:6;O7;G	-	-	$C_{25}H_{31}NO_8K^+$
1192	ST 24:0;O;S	-	-	$C_{24}H_{42}NO_4SK^+$
1193	ST 24:0;O3;GlCA/ST 24:1;O4;Hex	-	$C_{30}H_{50}O_9Na^+$	-
1194	ST 24:0;O4;GlCA/ST 24:1;O5;Hex	-	$C_{30}H_{50}O_{10}Na^+$	-
1195	ST 24:0;O4;Hex	-	$C_{30}H_{52}O_9Na^+$	-
1196	ST 24:0;O6;GlCA/ST 24:1;O7;Hex	-	$C_{30}H_{50}O_{12}Na^+$	-
1197	ST 24:0;O6;Hex	-	$C_{30}H_{52}O_{11}Na^+$	-
1198	ST 24:0;O6;HexNAC	-	$C_{32}H_{55}NO_{11}Na^+$	-
1199	ST 24:0;O7;GlCA/ST 24:1;O8;Hex	$C_{30}H_{51}O_{13}^+$	-	-
1200	ST 24:0;O7;Hex	$C_{30}H_{53}O_{12}^+$	-	-
1201	ST 24:0;O8	-	$C_{24}H_{42}O_8Na^+$	-
1202	ST 24:0;O8;G	-	$C_{26}H_{45}NO_9Na^+$	-
1203	ST 24:0;O8;GlCA	$C_{30}H_{51}O_{14}^+$	-	-
1204	ST 24:0;O8;Hex	-	$C_{30}H_{52}O_{13}Na^+$	-
1205	ST 24:1;O;S	-	-	$C_{24}H_{40}NO_4SK^+$
1206	ST 24:1;O5;HexNAC	-	$C_{32}H_{53}NO_{10}Na^+$	-
1207	ST 24:1;O6;HexNAC	-	$C_{32}H_{53}NO_{11}Na^+$	-
1208	ST 24:1;O7;GlCA/ST 24:2;O8;Hex	$C_{30}H_{49}O_{13}^+$	-	-
1209	ST 24:1;O7;T	-	-	$C_{26}H_{45}NO_9SK^+$
1210	ST 24:2;O;S	-	-	$C_{24}H_{38}NO_4SK^+$
1211	ST 24:2;O2;S	-	-	$C_{24}H_{38}NO_5SK^+$
1212	ST 24:2;O5;GlCA/ST 24:3;O6;Hex	$C_{30}H_{47}O_{11}^+$	-	-
1213	ST 24:2;O5;HexNAC	-	$C_{32}H_{51}NO_{10}Na^+$	-
1214	ST 24:2;O7;GlCA/ST 24:3;O8;Hex	$C_{30}H_{47}O_{13}^+$	-	-
1215	ST 24:2;O7;HexNAC	-	$C_{32}H_{51}NO_{12}Na^+$	-
1216	ST 24:3;O;S	-	-	$C_{24}H_{36}NO_4SK^+$
1217	ST 24:3;O2;S	-	-	$C_{24}H_{36}NO_5SK^+$
1218	ST 24:3;O3;G	-	$C_{26}H_{39}NO_4Na^+$	-
1219	ST 24:3;O4;S	-	$C_{24}H_{36}NO_7SNa^+$	-
1220	ST 24:3;O6;HexNAC	-	$C_{32}H_{49}NO_{11}Na^+$	-
1221	ST 24:3;O7;T	-	-	$C_{26}H_{41}NO_9SK^+$
1222	ST 24:4;O3	$C_{24}H_{35}O_3^+$	-	-
1223	ST 24:4;O3;T	-	-	$C_{26}H_{39}NO_5SK^+$
1224	ST 24:4;O4;T	-	-	$C_{26}H_{39}NO_6SK^+$
1225	ST 24:4;O6;T	-	-	$C_{26}H_{39}NO_8SK^+$
1226	ST 24:5;O3;S	$C_{24}H_{33}NO_6S^+$	-	$C_{24}H_{32}NO_6SK^+$
1227	ST 24:5;O4;T	-	-	$C_{26}H_{37}NO_6SK^+$
1228	ST 24:6;O;S	$C_{24}H_{31}NO_4S^+$	-	-
1229	ST 24:6;O2;S	$C_{24}H_{31}NO_5S^+$	-	$C_{24}H_{30}NO_5SK^+$
1230	ST 24:6;O3;T	$C_{26}H_{36}NO_5S^+$	-	$C_{26}H_{35}NO_5SK^+$
1231	ST 25:0;O5;GlCA/ST 25:1;O6;Hex	-	$C_{31}H_{52}O_{11}Na^+$	-
1232	ST 25:0;O6;GlCA/ST 25:1;O7;Hex	-	$C_{31}H_{52}O_{12}Na^+$	-
1233	ST 25:0;O6;Hex	-	$C_{31}H_{54}O_{11}Na^+$	-

1234	ST 25:0;O7;GlCA/ST 25:1;O8;Hex	-	$C_{31}H_{52}O_{13}Na^+$	-
1235	ST 25:0;O8	-	$C_{25}H_{44}O_8Na^+$	-
1236	ST 25:0;O8;G	$C_{27}H_{48}NO_9^+$	$C_{27}H_{47}NO_9Na^+$	-
1237	ST 25:0;O8;Hex	-	$C_{31}H_{54}O_{13}Na^+$	-
1238	ST 25:1;O;S	-	-	$C_{25}H_{42}NO_4SK^+$
1239	ST 25:1;O3;GlCA/ST 25:2;O4;Hex	-	$C_{31}H_{50}O_9Na^+$	-
1240	ST 25:1;O5;GlCA/ST 25:2;O6;Hex	$C_{31}H_{51}O_{11}^+$	$C_{31}H_{50}O_{11}Na^+$	-
1241	ST 25:1;O6;GlCA/ST 25:2;O7;Hex	$C_{31}H_{51}O_{12}^+$	$C_{31}H_{50}O_{12}Na^+$	-
1242	ST 25:1;O7;GlCA/ST 25:2;O8;Hex	-	$C_{31}H_{50}O_{13}Na^+$	-
1243	ST 25:2;O;S	-	-	$C_{25}H_{40}NO_4SK^+$
1244	ST 25:2;O2;S	-	-	$C_{25}H_{40}NO_5SK^+$
1245	ST 25:2;O5;GlCA/ST 25:3;O6;Hex	$C_{31}H_{49}O_{11}^+$	$C_{31}H_{48}O_{11}Na^+$	-
1246	ST 25:2;O6;GlCA/ST 25:3;O7;Hex	$C_{31}H_{49}O_{12}^+$	-	-
1247	ST 25:3;O;S	-	-	$C_{25}H_{38}NO_4SK^+$
1248	ST 25:3;O2;S	-	-	$C_{25}H_{38}NO_5SK^+$
1249	ST 25:3;O3;S	-	-	$C_{25}H_{38}NO_6SK^+$
1250	ST 25:3;O5;GlCA/ST 25:4;O6;Hex	$C_{31}H_{47}O_{11}^+$	-	-
1251	ST 25:4;O;S	-	-	$C_{25}H_{36}NO_4SK^+$
1252	ST 25:4;O3;GlCA/ST 25:5;O4;Hex	$C_{31}H_{45}O_9^+$	-	-
1253	ST 25:5;O;S	-	$C_{25}H_{34}NO_4SNa^+$	$C_{25}H_{34}NO_4SK^+$
1254	ST 25:5;O2;S	-	-	$C_{25}H_{34}NO_5SK^+$
1255	ST 25:6;O2;S	$C_{25}H_{33}NO_5S^+$	-	-
1256	ST 25:6;O3;T	$C_{27}H_{38}NO_5S^+$	-	-
1257	ST 25:7;O;S	$C_{25}H_{31}NO_4S^+$	-	-
1258	ST 25:7;O2;S	$C_{25}H_{31}NO_5S^+$	$C_{25}H_{30}NO_5SNa^+$	-
1259	ST 25:7;O3;T	$C_{27}H_{36}NO_5S^+$	$C_{27}H_{35}NO_5SNa^+$	-
1260	ST 25:7;O4;S	$C_{25}H_{31}NO_7S^+$	$C_{25}H_{30}NO_7SNa^+$	$C_{25}H_{30}NO_7SK^+$
1261	ST 25:7;O4;T	$C_{27}H_{36}NO_6S^+$	-	-
1262	ST 25:7;O5;S	-	$C_{25}H_{30}NO_8SNa^+$	$C_{25}H_{30}NO_8SK^+$
1263	ST 25:7;O5;T	$C_{27}H_{36}NO_7S^+$	-	-
1264	ST 26:0;O;S	-	-	$C_{26}H_{46}NO_4SK^+$
1265	ST 26:0;O4;GlCA/ST 26:1;O5;Hex	-	$C_{32}H_{54}O_{10}Na^+$	-
1266	ST 26:0;O8	-	$C_{26}H_{46}O_8Na^+$	-
1267	ST 26:0;O8;G	-	$C_{28}H_{49}NO_9Na^+$	-
1268	ST 26:0;O8;Hex	-	$C_{32}H_{56}O_{13}Na^+$	-
1269	ST 26:1;O;S	-	-	$C_{26}H_{44}NO_4SK^+$
1270	ST 26:1;O2;S	-	-	$C_{26}H_{44}NO_5SK^+$
1271	ST 26:1;O5;GlCA/ST 26:2;O6;Hex	-	$C_{32}H_{52}O_{11}Na^+$	-
1272	ST 26:2;O;S	-	-	$C_{26}H_{42}NO_4SK^+$
1273	ST 26:2;O2;S	-	-	$C_{26}H_{42}NO_5SK^+$
1274	ST 26:2;O5;GlCA/ST 26:3;O6;Hex	-	$C_{32}H_{50}O_{11}Na^+$	-
1275	ST 26:3;O;S	-	-	$C_{26}H_{40}NO_4SK^+$
1276	ST 26:3;O2;S	-	-	$C_{26}H_{40}NO_5SK^+$
1277	ST 26:3;O3;S	-	-	$C_{26}H_{40}NO_6SK^+$
1278	ST 26:3;O7;T	-	-	$C_{28}H_{45}NO_9SK^+$
1279	ST 26:4;O2;S	-	-	$C_{26}H_{38}NO_5SK^+$
1280	ST 26:4;O3;G	-	$C_{28}H_{41}NO_4Na^+$	-
1281	ST 26:4;O3;S	-	-	$C_{26}H_{38}NO_6SK^+$

1282	ST 26:4;O4;G	-	$C_{28}H_{41}NO_5Na^+$	-
1283	ST 26:5;O;S	-	-	$C_{26}H_{36}NO_4SK^+$
1284	ST 26:5;O2;S	-	-	$C_{26}H_{36}NO_5SK^+$
1285	ST 26:5;O3;T	-	-	$C_{28}H_{41}NO_5SK^+$
1286	ST 26:6;O;S	$C_{26}H_{35}NO_4S^+$	-	-
1287	ST 26:6;O3;T	$C_{28}H_{40}NO_5S^+$	-	-
1288	ST 26:6;O4;S	-	-	$C_{26}H_{34}NO_7SK^+$
1289	ST 26:6;O4;T	$C_{28}H_{40}NO_6S^+$	-	-
1290	ST 26:7;O;Hex	-	$C_{32}H_{42}O_6Na^+$	-
1291	ST 26:7;O;S	$C_{26}H_{33}NO_4S^+$	-	-
1292	ST 26:7;O3;T	$C_{28}H_{38}NO_5S^+$	$C_{28}H_{37}NO_5SNa^+$	-
1293	ST 26:7;O4;T	$C_{28}H_{38}NO_6S^+$	-	-
1294	ST 27:0;O6;GlCA/ST 27:1;O7;Hex	-	$C_{33}H_{56}O_{12}Na^+$	-
1295	ST 27:0;O8	-	$C_{27}H_{48}O_8Na^+$	-
1296	ST 27:0;O8;G	-	$C_{29}H_{51}NO_9Na^+$	-
1297	ST 27:0;O8;HexNAC	-	$C_{35}H_{61}NO_{13}Na^+$	-
1298	ST 27:1;O;S	-	-	$C_{27}H_{46}NO_4SK^+$
1299	ST 27:1;O2;S	-	-	$C_{27}H_{46}NO_5SK^+$
1300	ST 27:1;O3;S	-	-	$C_{27}H_{46}NO_6SK^+$
1301	ST 27:1;O6;GlCA/ST 27:2;O7;Hex	$C_{33}H_{55}O_{12}^+$	-	-
1302	ST 27:1;O8;GlCA	$C_{33}H_{55}O_{14}^+$	-	-
1303	ST 27:2;O;S	-	-	$C_{27}H_{44}NO_4SK^+$
1304	ST 27:2;O2;S	-	-	$C_{27}H_{44}NO_5SK^+$
1305	ST 27:2;O5;GlCA/ST 27:3;O6;Hex	$C_{33}H_{53}O_{11}^+$	-	-
1306	ST 27:2;O6;GlCA/ST 27:3;O7;Hex	-	$C_{33}H_{52}O_{12}Na^+$	-
1307	ST 27:3;O;S	-	-	$C_{27}H_{42}NO_4SK^+$
1308	ST 27:3;O2;S	-	-	$C_{27}H_{42}NO_5SK^+$
1309	ST 27:3;O5;GlCA/ST 27:4;O6;Hex	$C_{33}H_{51}O_{11}^+$	-	-
1310	ST 27:4;O;S	$C_{27}H_{41}NO_4S^+$	-	$C_{27}H_{40}NO_4SK^+$
1311	ST 27:4;O2;S	-	-	$C_{27}H_{40}NO_5SK^+$
1312	ST 27:4;O3;S	-	-	$C_{27}H_{40}NO_6SK^+$
1313	ST 27:4;O4;G	-	$C_{29}H_{43}NO_5Na^+$	-
1314	ST 27:4;O6;GlCA/ST 27:5;O7;Hex	$C_{33}H_{49}O_{12}^+$	-	-
1315	ST 27:4;O7;GlCA/ST 27:5;O8;Hex	$C_{33}H_{49}O_{13}^+$	-	-
1316	ST 27:5;O;S	$C_{27}H_{39}NO_4S^+$	-	$C_{27}H_{38}NO_4SK^+$
1317	ST 27:5;O2;S	-	-	$C_{27}H_{38}NO_5SK^+$
1318	ST 27:6;O;S	$C_{27}H_{37}NO_4S^+$	-	$C_{27}H_{36}NO_4SK^+$
1319	ST 27:6;O2;S	$C_{27}H_{37}NO_5S^+$	-	-
1320	ST 27:6;O3;T	$C_{29}H_{42}NO_5S^+$	-	$C_{29}H_{41}NO_5SK^+$
1321	ST 27:6;O4;T	$C_{29}H_{42}NO_6S^+$	-	$C_{29}H_{41}NO_6SK^+$
1322	ST 27:6;O6;T	-	$C_{29}H_{41}NO_8SNa^+$	-
1323	ST 27:7;O;S	$C_{27}H_{35}NO_4S^+$	$C_{27}H_{34}NO_4SNa^+$	-
1324	ST 27:7;O2;S	$C_{27}H_{35}NO_5S^+$	-	-
1325	ST 27:7;O3;T	$C_{29}H_{40}NO_5S^+$	-	-
1326	ST 27:7;O4;T	$C_{29}H_{40}NO_6S^+$	-	-
1327	ST 27:7;O5;T	-	$C_{29}H_{39}NO_7SNa^+$	-
1328	ST 28:0;O2;S	-	-	$C_{28}H_{50}NO_5SK^+$
1329	ST 28:0;O3;S	$C_{28}H_{51}NO_6S^+$	-	$C_{28}H_{50}NO_6SK^+$

1330	ST 28:0;O4;GlCA/ST 28:1;O5;Hex	-	$C_{34}H_{58}O_{10}Na^+$	-
1331	ST 28:0;O4;S	-	-	$C_{28}H_{50}NO_7SK^+$
1332	ST 28:0;O7;T	-	-	$C_{30}H_{55}NO_9SK^+$
1333	ST 28:0;O8	-	$C_{28}H_{50}O_8Na^+$	-
1334	ST 28:0;O8;Hex	$C_{34}H_{61}O_{13}^+$	$C_{34}H_{60}O_{13}Na^+$	$C_{34}H_{60}O_{13}K^+$
1335	ST 28:1;O;S	-	-	$C_{28}H_{48}NO_4SK^+$
1336	ST 28:1;O2;S	-	-	$C_{28}H_{48}NO_5SK^+$
1337	ST 28:1;O8;GlCA	-	$C_{34}H_{56}O_{14}Na^+$	-
1338	ST 28:2;O;S	-	-	$C_{28}H_{46}NO_4SK^+$
1339	ST 28:2;O2;S	-	-	$C_{28}H_{46}NO_5SK^+$
1340	ST 28:2;O3;S	-	-	$C_{28}H_{46}NO_6SK^+$
1341	ST 28:3;O;S	-	-	$C_{28}H_{44}NO_4SK^+$
1342	ST 28:3;O2;S	-	-	$C_{28}H_{44}NO_5SK^+$
1343	ST 28:3;O4;S	-	-	$C_{28}H_{44}NO_7SK^+$
1344	ST 28:4;O;S	-	-	$C_{28}H_{42}NO_4SK^+$
1345	ST 28:4;O2;S	-	-	$C_{28}H_{42}NO_5SK^+$
1346	ST 28:4;O4;G	-	$C_{30}H_{45}NO_5Na^+$	-
1347	ST 28:4;O7;T	-	-	$C_{30}H_{47}NO_9SK^+$
1348	ST 28:5;O;S	-	-	$C_{28}H_{40}NO_4SK^+$
1349	ST 28:5;O3;S	-	-	$C_{28}H_{40}NO_6SK^+$
1350	ST 28:5;O3;T	-	-	$C_{30}H_{45}NO_5SK^+$
1351	ST 28:5;O4;G	-	$C_{30}H_{43}NO_5Na^+$	-
1352	ST 28:5;O4;T	-	-	$C_{30}H_{45}NO_6SK^+$
1353	ST 28:6;O;S	$C_{28}H_{39}NO_4S^+$	-	-
1354	ST 28:6;O2;S	$C_{28}H_{39}NO_5S^+$	-	$C_{28}H_{38}NO_5SK^+$
1355	ST 28:6;O3;T	$C_{30}H_{44}NO_5S^+$	-	-
1356	ST 28:6;O4;T	$C_{30}H_{44}NO_6S^+$	-	$C_{30}H_{43}NO_6SK^+$
1357	ST 28:6;O6;T	$C_{30}H_{44}NO_8S^+$	-	-
1358	ST 28:7;O;Hex	-	$C_{34}H_{46}O_6Na^+$	-
1359	ST 28:7;O;S	$C_{28}H_{37}NO_4S^+$	$C_{28}H_{36}NO_4SNa^+$	-
1360	ST 28:7;O3;T	$C_{30}H_{42}NO_5S^+$	-	$C_{30}H_{41}NO_5SK^+$
1361	ST 28:7;O4;T	$C_{30}H_{42}NO_6S^+$	-	$C_{30}H_{41}NO_6SK^+$
1362	ST 29:0;O2;S	-	-	$C_{29}H_{52}NO_5SK^+$
1363	ST 29:0;O3;S	-	-	$C_{29}H_{52}NO_6SK^+$
1364	ST 29:0;O4;S	-	-	$C_{29}H_{52}NO_7SK^+$
1365	ST 29:0;O8	-	$C_{29}H_{52}O_8Na^+$	-
1366	ST 29:0;O8;G	-	$C_{31}H_{55}NO_9Na^+$	-
1367	ST 29:1;O2;S	-	-	$C_{29}H_{50}NO_5SK^+$
1368	ST 29:1;O7;GlCA/ST 29:2;O8;Hex	$C_{35}H_{59}O_{13}^+$	-	-
1369	ST 29:2;O;S	-	-	$C_{29}H_{48}NO_4SK^+$
1370	ST 29:2;O2;S	-	-	$C_{29}H_{48}NO_5SK^+$
1371	ST 29:2;O3;S	-	-	$C_{29}H_{48}NO_6SK^+$
1372	ST 29:2;O7;GlCA/ST 29:3;O8;Hex	-	$C_{35}H_{56}O_{13}Na^+$	-
1373	ST 29:3;O6;GlCA/ST 29:4;O7;Hex	-	$C_{35}H_{54}O_{12}Na^+$	-
1374	ST 29:3;O8;HexNAC	-	$C_{37}H_{59}NO_{13}Na^+$	-
1375	ST 29:4;O2;S	-	-	$C_{29}H_{44}NO_5SK^+$
1376	ST 29:4;O3;S	-	-	$C_{29}H_{44}NO_6SK^+$
1377	ST 29:4;O4;G	-	$C_{31}H_{47}NO_5Na^+$	-

1378	ST 29:5;O;S	-	-	$C_{29}H_{42}NO_4SK^+$
1379	ST 29:5;O2;S	-	-	$C_{29}H_{42}NO_5SK^+$
1380	ST 29:5;O3;S	-	-	$C_{29}H_{42}NO_6SK^+$
1381	ST 29:5;O3;T	-	-	$C_{31}H_{47}NO_5SK^+$
1382	ST 29:5;O4;G	-	$C_{31}H_{45}NO_5Na^+$	-
1383	ST 29:5;O5;T	-	-	$C_{31}H_{47}NO_7SK^+$
1384	ST 29:5;O7;T	$C_{31}H_{48}NO_9S^+$	-	-
1385	ST 29:6;O;S	-	$C_{29}H_{40}NO_4SNa^+$	$C_{29}H_{40}NO_4SK^+$
1386	ST 29:6;O3;T	$C_{31}H_{46}NO_5S^+$	-	$C_{31}H_{45}NO_5SK^+$
1387	ST 29:6;O4;T	-	$C_{31}H_{45}NO_6SNa^+$	-
1388	ST 29:6;O5;T	$C_{31}H_{46}NO_7S^+$	-	-
1389	ST 29:6;O6;T	$C_{31}H_{46}NO_8S^+$	-	-
1390	ST 29:7;O;S	$C_{29}H_{39}NO_4S^+$	$C_{29}H_{38}NO_4SNa^+$	-
1391	ST 29:7;O2;S	$C_{29}H_{39}NO_5S^+$	-	$C_{29}H_{38}NO_5SK^+$
1392	ST 29:7;O3;T	$C_{31}H_{44}NO_5S^+$	-	-
1393	ST 29:7;O4;S	$C_{29}H_{39}NO_7S^+$	-	-
1394	ST 29:7;O4;T	$C_{31}H_{44}NO_6S^+$	$C_{31}H_{43}NO_6SNa^+$	-
1395	ST 29:7;O5;T	$C_{31}H_{44}NO_7S^+$	-	-
1396	ST 30:0;O2;S	$C_{30}H_{55}NO_5S^+$	-	$C_{30}H_{54}NO_5SK^+$
1397	ST 30:0;O3;S	$C_{30}H_{55}NO_6S^+$	-	-
1398	ST 30:0;O4;S	$C_{30}H_{55}NO_7S^+$	-	$C_{30}H_{54}NO_7SK^+$
1399	ST 30:0;O5;S	-	-	$C_{30}H_{54}NO_8SK^+$
1400	ST 30:0;O8	-	$C_{30}H_{54}O_8Na^+$	-
1401	ST 30:0;O8;Hex	$C_{36}H_{65}O_{13}^+$	$C_{36}H_{64}O_{13}Na^+$	-
1402	ST 30:1;O2;S	$C_{30}H_{53}NO_5S^+$	-	$C_{30}H_{52}NO_5SK^+$
1403	ST 30:1;O3;S	-	-	$C_{30}H_{52}NO_6SK^+$
1404	ST 30:1;O4;S	-	-	$C_{30}H_{52}NO_7SK^+$
1405	ST 30:2;O;S	-	-	$C_{30}H_{50}NO_4SK^+$
1406	ST 30:2;O4;S	-	-	$C_{30}H_{50}NO_7SK^+$
1407	ST 30:3;O;S	-	$C_{30}H_{48}NO_4SNa^+$	$C_{30}H_{48}NO_4SK^+$
1408	ST 30:3;O2;S	-	-	$C_{30}H_{48}NO_5SK^+$
1409	ST 30:3;O6;S	-	-	$C_{30}H_{48}NO_9SK^+$
1410	ST 30:3;O7;S	-	-	$C_{30}H_{48}NO_{10}SK^+$
1411	ST 30:3;O8;GlCA	-	-	$C_{36}H_{56}O_{14}K^+$
1412	ST 30:4;O;S	-	-	$C_{30}H_{46}NO_4SK^+$
1413	ST 30:4;O2;S	-	-	$C_{30}H_{46}NO_5SK^+$
1414	ST 30:4;O3;S	-	-	$C_{30}H_{46}NO_6SK^+$
1415	ST 30:4;O6;S	-	-	$C_{30}H_{46}NO_9SK^+$
1416	ST 30:5;O;S	-	-	$C_{30}H_{44}NO_4SK^+$
1417	ST 30:5;O2;S	-	-	$C_{30}H_{44}NO_5SK^+$
1418	ST 30:5;O3;S	$C_{30}H_{45}NO_6S^+$	-	$C_{30}H_{44}NO_6SK^+$
1419	ST 30:5;O4;S	-	-	$C_{30}H_{44}NO_7SK^+$
1420	ST 30:5;O5;S	-	-	$C_{30}H_{44}NO_8SK^+$
1421	ST 30:5;O6;T	-	-	$C_{32}H_{49}NO_8SK^+$
1422	ST 30:6;O;S	$C_{30}H_{43}NO_4S^+$	$C_{30}H_{42}NO_4SNa^+$	$C_{30}H_{42}NO_4SK^+$
1423	ST 30:6;O2;S	$C_{30}H_{43}NO_5S^+$	-	-
1424	ST 30:6;O3;T	$C_{32}H_{48}NO_5S^+$	$C_{32}H_{47}NO_5SNa^+$	$C_{32}H_{47}NO_5SK^+$
1425	ST 30:6;O4;T	$C_{32}H_{48}NO_6S^+$	-	-

1426	ST 30:6;O5;T	-	$C_{32}H_{47}NO_7SNa^+$	-
1427	ST 30:6;O7;T	$C_{32}H_{48}NO_9S^+$	-	-
1428	ST 30:7;O;Hex/TG 33:9	-	$C_{36}H_{50}O_6Na^+$	-
1429	ST 30:7;O;S	$C_{30}H_{41}NO_4S^+$	$C_{30}H_{40}NO_4SNa^+$	-
1430	ST 30:7;O3;G	-	-	$C_{32}H_{43}NO_4K^+$
1431	ST 30:7;O3;S	$C_{30}H_{41}NO_6S^+$	-	-
1432	ST 30:7;O3;T	$C_{32}H_{46}NO_5S^+$	$C_{32}H_{45}NO_5SNa^+$	-
1433	ST 30:7;O4;GlCA	-	-	$C_{36}H_{48}O_{10}K^+$
1434	ST 30:7;O4;T	$C_{32}H_{46}NO_6S^+$	-	-
1435	ST 30:7;O8;T	$C_{32}H_{46}NO_{10}S^+$	-	-
1436	TG 35:2;O2/TG O-35:3;O3	-	$C_{38}H_{68}O_8Na^+$	-
1437	TG 36:2;O2/TG O-36:3;O3	-	$C_{39}H_{70}O_8Na^+$	-
1438	TG 37:1;O2/TG O-37:2;O3	-	$C_{40}H_{74}O_8Na^+$	-
1439	TG 37:1;O3	-	-	$C_{40}H_{74}O_9K^+$
1440	TG 38:1;O2/TG O-38:2;O3	-	$C_{41}H_{76}O_8Na^+$	-
1441	TG 38:1;O3	-	$C_{41}H_{76}O_9Na^+$	-
1442	TG 38:10	-	-	$C_{41}H_{58}O_6K^+$
1443	TG 38:2;O2/TG O-38:3;O3	-	$C_{41}H_{74}O_8Na^+$	-
1444	TG 38:3;O2/TG O-38:4;O3	-	$C_{41}H_{72}O_8Na^+$	-
1445	TG 38:4;O2/TG O-38:5;O3	-	-	$C_{41}H_{70}O_8K^+$
1446	TG 39:1;O2/TG O-39:2;O3	-	$C_{42}H_{78}O_8Na^+$	-
1447	TG 39:2;O2/TG O-39:3;O3	-	$C_{42}H_{76}O_8Na^+$	-
1448	TG 40:2;O2/TG O-40:3;O3	-	$C_{43}H_{78}O_8Na^+$	-
1449	TG 40:6;O2/TG O-40:7;O3	$C_{43}H_{71}O_8^+$	$C_{43}H_{70}O_8Na^+$	-
1450	TG 41:1;O3	-	$C_{44}H_{82}O_9Na^+$	-
1451	TG 41:2;O3	-	$C_{44}H_{80}O_9Na^+$	-
1452	TG 42:12;O3	-	$C_{45}H_{62}O_9Na^+$	-
1453	TG 43:4;O3	$C_{46}H_{81}O_9^+$	-	-
1454	TG 43:5;O3	$C_{46}H_{79}O_9^+$	-	-
1455	TG 45:10;O3	-	$C_{48}H_{72}O_9Na^+$	-

Supplementary Table S3. Quinolone assignments in the biofilm spectrum.

m/z	Assignment	dev. (ppm)	Area
173.0834	$C_{11}H_{11}NO^+$	-0.665	394507
174.0912	$C_{11}H_{12}NO^+$	-0.80475	5118907
176.1068	$C_{11}H_{14}NO^+$	-1.07946	2178951
185.0834	$C_{12}H_{11}NO^+$	-0.62188	486977.1
186.0912	$C_{12}H_{12}NO^+$	-0.75286	5787236
188.1068	$C_{12}H_{14}NO^+$	-1.0106	1781406
189.0783	$C_{11}H_{11}NO_2^+$	-0.68807	540246.3
190.0861	$C_{11}H_{12}NO_2^+$	-0.81595	5197015
190.1225	$C_{12}H_{16}NO^+$	-0.73689	522215.8
192.1017	$C_{11}H_{14}NO_2^+$	-1.06766	2171638
196.0732	$C_{11}H_{11}NONa^+$	-0.42892	1443582
198.0912	$C_{13}H_{12}NO^+$	-0.70725	1314402

200.1068	$C_{13}H_{14}NO^+$	-0.94999	2117241
202.0861	$C_{12}H_{12}NO_2^+$	-0.76749	3324959
202.1225	$C_{13}H_{16}NO^+$	-0.69314	1029142
204.1018	$C_{12}H_{14}NO_2^+$	-0.51494	1919263
204.1382	$C_{13}H_{18}NO^+$	-0.44137	227063.9
206.1175	$C_{12}H_{16}NO_2^+$	-0.26732	510815.6
208.0732	$C_{12}H_{11}NONa^+$	-0.40418	450126.7
212.0681	$C_{11}H_{11}NO_2Na^+$	-0.4673	2574382
212.1069	$C_{14}H_{14}NO^+$	-0.42479	373862.3
214.0861	$C_{13}H_{12}NO_2^+$	-0.72447	275410.7
214.1225	$C_{14}H_{16}NO^+$	-0.6543	983991
216.1018	$C_{13}H_{14}NO_2^+$	-0.48634	1457260
216.1382	$C_{14}H_{18}NO^+$	-0.41686	348657.4
218.1175	$C_{13}H_{16}NO_2^+$	-0.25262	967347.1
218.1539	$C_{14}H_{20}NO^+$	-0.18382	96908.21
220.1331	$C_{13}H_{18}NO_2^+$	-0.47744	205866.9
222.089	$C_{13}H_{13}NONa^+$	0.296728	130139.9
224.0681	$C_{12}H_{11}NO_2Na^+$	-0.44228	883854.5
224.1069	$C_{15}H_{14}NO^+$	-0.40204	67436.55
226.1225	$C_{15}H_{16}NO^+$	-0.61958	205678.6
228.1382	$C_{15}H_{18}NO^+$	-0.39494	483951.6
230.1174	$C_{14}H_{16}NO_2^+$	-0.674	716334.1
230.1539	$C_{15}H_{20}NO^+$	-0.17423	119684.5
232.1331	$C_{14}H_{18}NO_2^+$	-0.45276	384077.8
234.1487	$C_{14}H_{20}NO_2^+$	-0.6624	103607.6
240.1382	$C_{16}H_{18}NO^+$	-0.3752	139299.6
242.1174	$C_{15}H_{16}NO_2^+$	-0.6406	122177.4
242.1538	$C_{16}H_{20}NO^+$	-0.57856	2312591
243.1617	$C_{16}H_{21}NO^+$	-0.26772	62980.18
244.1329	$C_{15}H_{18}NO_2^+$	-1.24973	664627.9
244.1695	$C_{16}H_{22}NO^+$	-0.36901	19804885
246.1488	$C_{15}H_{20}NO_2^+$	-0.22385	167221.5
254.1538	$C_{17}H_{20}NO^+$	-0.55124	66809.81
256.1331	$C_{16}H_{18}NO_2^+$	-0.41033	139819.8
256.1695	$C_{17}H_{22}NO^+$	-0.35172	154590.1
258.1487	$C_{16}H_{20}NO_2^+$	-0.60082	6088625
258.1851	$C_{17}H_{24}NO^+$	-0.54263	186413.6
259.1565	$C_{16}H_{21}NO_2^+$	-0.69495	938392
260.1066	$C_{18}H_{14}NO^+$	-1.49977	81008.34
260.1644	$C_{16}H_{22}NO_2^+$	-0.40398	86361649
262.1801	$C_{16}H_{24}NO_2^+$	-0.21016	63493.69
268.1695	$C_{18}H_{22}NO^+$	-0.33598	1516185
269.1773	$C_{18}H_{23}NO^+$	-0.4276	500788.5
270.1851	$C_{18}H_{24}NO^+$	-0.51853	1.09E+08
271.1929	$C_{18}H_{25}NO^+$	-0.60879	1825354
272.1645	$C_{17}H_{22}NO_2^+$	-0.01874	157123.5
272.2007	$C_{18}H_{26}NO^+$	-0.69838	1.65E+08
273.2082	$C_{18}H_{27}NO^+$	-1.88537	69359.08

274.18	$C_{17}H_{24}NO_2^+$	-0.56569	333169.6
274.2164	$C_{18}H_{28}NO^+$	-0.51091	30429.18
276.1381	$C_{19}H_{18}NO^+$	-0.68842	74921.64
276.1957	$C_{17}H_{26}NO_2^+$	-0.38053	55856.48
281.1385	$C_{16}H_{20}NO_2Na^+$	-0.44142	136706.2
282.1463	$C_{16}H_{21}NO_2Na^+$	-0.52845	6600515
284.1644	$C_{18}H_{22}NO_2^+$	-0.36986	494614.8
284.2008	$C_{19}H_{26}NO^+$	-0.31703	290512.6
285.1722	$C_{18}H_{23}NO_2^+$	-0.45622	251625.3
286.18	$C_{18}H_{24}NO_2^+$	-0.54197	45541038
286.2165	$C_{19}H_{28}NO^+$	-0.1401	129839.9
287.1879	$C_{18}H_{25}NO_2^+$	-0.27891	993430.5
288.138	$C_{20}H_{18}NO^+$	-1.00681	76554.89
288.1957	$C_{18}H_{26}NO_2^+$	-0.36468	65026622
289.2033	$C_{18}H_{27}NO_2^+$	-1.14141	94702.69
290.1538	$C_{20}H_{20}NO^+$	-0.48285	58256.8
290.2113	$C_{18}H_{28}NO_2^+$	-0.53444	114916.4
292.167	$C_{18}H_{23}NONa^+$	-0.63012	7835033
294.1828	$C_{18}H_{25}NONa^+$	-0.11591	14328872
296.1046	$C_{19}H_{15}NONa^+$	0.053697	54413.83
296.2008	$C_{20}H_{26}NO^+$	-0.30419	2030946
297.1127	$C_{19}H_{16}NONa^+$	0.979091	56170.89
297.2087	$C_{20}H_{27}NO^+$	-0.05081	60920.86
298.1203	$C_{19}H_{17}NONa^+$	0.221052	824685.9
298.2164	$C_{20}H_{28}NO^+$	-0.46979	31600143
299.2241	$C_{20}H_{29}NO^+$	-0.88596	37908.75
300.1382	$C_{21}H_{18}NO^+$	-0.30019	294323.6
300.1957	$C_{19}H_{26}NO_2^+$	-0.3501	225213.8
300.2321	$C_{20}H_{30}NO^+$	-0.3001	5754397
302.1536	$C_{21}H_{20}NO^+$	-1.12559	73314.88
302.2113	$C_{19}H_{28}NO_2^+$	-0.51322	134695.7
305.0841	$C_{22}H_{11}NO^+$	1.91718	110399.1
308.1619	$C_{18}H_{23}NO_2Na^+$	-0.64609	2171496
310.0868	$C_{21}H_{12}NO_2^+$	1.757253	347513.4
310.1776	$C_{18}H_{25}NO_2Na^+$	-0.48069	4063387
310.2165	$C_{21}H_{28}NO^+$	-0.12926	45139.95
312.1957	$C_{20}H_{26}NO_2^+$	-0.33665	551089.7
312.2321	$C_{21}H_{30}NO^+$	-0.28857	91016.92
314.2113	$C_{20}H_{28}NO_2^+$	-0.49362	11452431
316.2271	$C_{20}H_{30}NO_2^+$	-0.01613	630480.1
317.0839	$C_{23}H_{11}NO^+$	1.213876	120562.4
318.1487	$C_{21}H_{20}NO_2^+$	-0.48751	86866.02
320.1983	$C_{20}H_{27}NONa^+$	-0.57496	847676.1
322.2134	$C_{20}H_{29}NONa^+$	-2.2783	107797.8
324.136	$C_{21}H_{19}NONa^+$	0.357566	276980
324.2322	$C_{22}H_{30}NO^+$	0.030534	143975.4
326.1516	$C_{21}H_{21}NONa^+$	0.202053	468387
326.2478	$C_{22}H_{32}NO^+$	-0.12291	550583.6

328.2271	$C_{21}H_{30}NO_2^+$	-0.01554	45150.86
329.0843	$C_{24}H_{11}NO^+$	2.385109	259579.8
330.1484	$C_{22}H_{20}NO_2^+$	-1.37847	44322.09
331.0999	$C_{24}H_{13}NO^+$	2.219577	2948384
332.0688	$C_{21}H_{11}NO_2Na^+$	1.809568	40107.23
333.079	$C_{23}H_{11}NO_2^+$	1.711008	83792.31
333.1156	$C_{24}H_{15}NO^+$	2.356245	1425498
336.1931	$C_{20}H_{27}NO_2Na^+$	-0.88967	259277.6
340.2272	$C_{22}H_{30}NO_2^+$	0.278931	50720.09
342.2427	$C_{22}H_{32}NO_2^+$	-0.161	91605.44
345.0789	$C_{24}H_{11}NO_2^+$	1.361719	90914.03
347.0948	$C_{24}H_{13}NO_2^+$	2.074077	1542470
349.1105	$C_{24}H_{15}NO_2^+$	2.205324	1244247
352.1673	$C_{23}H_{23}NONa^+$	0.329105	62818.98
380.2558	$C_{23}H_{35}NO_2Na^+$	-0.52359	26375.89
405.2648	$C_{25}H_{36}NO_2Na^+$	2.408061	268711.1

Supplementary Table S4. Acyl homoserine lactone (AHL) assignments in the biofilm spectrum.

m/z	Assignment	Description	Possible AHL identity	Deviation (ppm)
85.0286	$C_4H_5O_2^+$	AHL		2.155019
86.0364	$C_4H_6O_2^+$	AHL		2.256652
100.0393	$C_4H_6NO_2^+$	AHL		0.293813
101.0472	$C_4H_7NO_2^+$	AHL		0.286452
128.0341	$C_5H_6NO_3^+$	AHL		-0.9034
129.0421	$C_5H_7NO_3^+$	AHL		0.138799
142.0498	$C_6H_8NO_3^+$	AHL		-0.74324
143.0576	$C_6H_9NO_3^+$	AHL		-0.5552
171.0527	$C_7H_9NO_4^+$	AHL		0.658131
185.0684	$C_8H_{11}NO_4^+$	AHL		1.000031
199.0841	$C_9H_{13}NO_4^+$	AHL		0.801109
213.0998	$C_{10}H_{15}NO_4^+$	AHL	N-(3-Oxohexanoyl)-L-homoserine lactone	0.989324
227.1153	$C_{11}H_{17}NO_4^+$	AHL		0.416046

Supplementary Table S5. Salt ion assignments in the biofilm spectrum.

m/z	Assignment	Dev. (ppm)	Area
86.9608	$PO_2H_1Na^+$	1.88	425214
87.9771	$O_4H_1Na^+$	4.49	21378163
93.9429	$O_2H_0NaK^+$	1.53	6382475
94.9297	$O_1H_1K_2^+$	1.02	6538253
94.9507	$O_2H_1NaK^+$	1.25	1483804
97.9765	$PO_4H_3^+$	1.56	1425737
103.9509	$O_4H_1K^+$	2.48	22507293
103.9634	$PO_3H_2Na^+$	0.23	269785.5
104.9712	$PO_3H_3Na^+$	-0.01	1.08E+08

108.9425	$PO_2H_0Na_2^+$	-0.74	202775.8
109.9167	$O_2H_0K_2^+$	0.06	1153634
112.9635	$PO_5H_2^+$	0.56	762672.2
113.9713	$PO_5H_3^+$	0.33	1678752
120.9451	$PO_3H_3K^+$	-0.32	46638865
120.9566	$O_4H_2Na^+$	-0.01	2574076
120.9660	$PO_4H_3Na^+$	-0.96	2.17E+09
124.9374	$PO_3H_0Na_2^+$	-0.76	687655.7
126.9530	$PO_3H_2Na_2^+$	-1.14	9692855
128.9584	$PO_6H_2^+$	0.37	498388.7
134.9454	$PO_5H_1Na^+$	0.14	339435
135.9531	$PO_5H_2Na^+$	-0.78	1079120
136.9306	$O_4H_2K^+$	0.45	1750197
136.9400	$PO_4H_3K^+$	-0.39	9.61E+08
136.9530	$P_2O_3H_4Na^+$	1.55	380805.2
136.9611	$PO_5H_3Na^+$	0.50	1943757
142.9270	$PO_3H_2NaK^+$	-0.57	6081072
142.9385	$O_4H_1Na_2^+$	-0.32	26802537
142.9480	$PO_4H_2Na_2^+$	-0.42	3.65E+08
148.9352	$PO_3H_1Na_3^+$	0.74	545999.6
150.9403	$PO_6H_1Na^+$	0.03	335764.2
152.9349	$PO_5H_3K^+$	-0.45	210521.7
152.9559	$PO_6H_3Na^+$	-0.30	57198521
158.9009	$PO_3H_2K_2^+$	-0.75	393602.2
158.9124	$O_4H_1NaK^+$	-0.52	19613939
158.9219	$PO_4H_2NaK^+$	-0.61	2.42E+08
164.9091	$PO_3H_1Na_2K^+$	0.45	1096847
164.9204	$O_4H_0Na_3^+$	-0.54	535340.9
164.9299	$PO_4H_1Na_3^+$	-0.63	3923869
168.9299	$PO_6H_3K^+$	0.10	7453073
174.8864	$O_4H_1K_2^+$	-0.11	1940562
174.8958	$PO_4H_2K_2^+$	-0.77	49021827
174.9380	$PO_6H_2Na_2^+$	0.63	9780833
180.8831	$PO_3H_1NaK_2^+$	0.76	261430.8
180.8943	$O_4H_0Na_2K^+$	-0.70	927673.4
180.9038	$PO_4H_1Na_2K^+$	-0.78	5478357
190.9119	$PO_6H_2NaK^+$	0.38	3393459
196.8684	$O_4H_0NaK_2^+$	0.19	238865.8
196.8779	$PO_4H_1NaK_2^+$	0.11	1498559
200.9066	$P_3O_3H_5Na^+$	0.78	227568.2
200.9323	$P_2O_7H_4Na^+$	-0.73	12129428
206.8859	$PO_6H_2K_2^+$	0.66	119238.3
212.8518	$PO_4H_1K_3^+$	-0.07	76937.35
216.8807	$P_3O_3H_5K^+$	1.47	468035.2
216.9064	$P_2O_7H_4K^+$	0.07	2865109
222.9143	$P_2O_7H_3Na_2^+$	-0.41	1168654
238.8882	$P_2O_7H_3NaK^+$	-0.54	665093.1
242.8834	$P_3O_2H_3Na_5^+$	2.12	1542091

262.8974	$PO_8H_3Na_3^+$	0.06	193950.1
262.9069	$P_2O_8H_4Na_3^+$	0.00	2197015
265.7788	$P_3O_1H_1K_4^+$	1.89	102510.1
274.8968	$O_{10}H_0Na_5^+$	-2.35	715246
278.8714	$PO_8H_3Na_2K^+$	0.28	306374.5
278.8809	$P_2O_8H_4Na_2K^+$	0.23	3117327
280.8983	$P_3O_{10}H_5Na^+$	-1.70	68060.37
290.8743	$O_9H_0Na_5^+$	-1.04	99767.97
294.8453	$PO_8H_3NaK_2^+$	0.14	77797.71
294.8549	$P_5O_2H_7Na_3^+$	2.24	648768.6
294.8549	$P_2O_8H_4NaK_2^+$	0.43	648768.6
294.8968	$P_2O_{10}H_4Na_3^+$	0.24	227368.4
300.8534	$PO_8H_2Na_3K^+$	0.45	49146.47
310.8708	$P_2O_{10}H_4Na_2K^+$	0.43	81214.34
368.7698	$P_4O_8H_0Na_2K^+$	1.82	25494.29
368.7698	$P_4O_1H_4Na_5K_2^+$	2.07	25494.29
368.7698	$PO_7H_1Na_3K_4^+$	0.62	25494.29
368.7698	$O_4H_2Na_5K_4^+$	-1.90	25494.29
369.7493	$PO_8H_0NaK_4^+$	0.28	51365.38
370.7572	$PO_8H_1NaK_4^+$	0.48	125207.1
377.7381	$P_4O_5H_2NaK_3^+$	1.12	33506.9
378.7461	$P_4O_5H_3NaK_3^+$	1.58	94482.02
378.7461	$O_8H_1NaK_5^+$	-2.29	94482.02
394.7434	$P_5O_8H_2K_2^+$	1.75	308749.2
394.7434	$P_5O_1H_6Na_3K_3^+$	1.98	308749.2
394.7434	$P_2O_7H_3NaK_5^+$	0.63	308749.2
438.7277	$P_5O_9H_0Na_3K^+$	0.28	63654.35
438.7277	$P_2O_8H_1Na_4K_4^+$	-0.73	63654.35
441.7665	$P_4O_9H_4Na_4K_2^+$	-0.04	19328.01
471.6921	$P_4O_4H_4Na_4K_4^+$	1.43	20111
474.6732	$P_4O_5H_0Na_5K_4^+$	0.97	20428.97
480.6937	$P_5O_8H_3Na_2K_3^+$	-0.16	22008.84
480.6937	$P_5O_1H_7Na_5K_4^+$	0.03	22008.84
489.6981	$P_4O_6H_6Na_3K_5^+$	-1.37	26783.72
489.6981	$P_2O_8H_4Na_3K_5^+$	1.22	26783.72
539.6299	$P_5O_6H_2Na_4K_5^+$	-2.04	29428.79
539.6299	$P_3O_8H_0Na_4K_5^+$	0.31	29428.79

Supplementary Table S6. Substrate ion assignments in the biofilm spectrum.

m/z	Assignment	Dev. (ppm)	Area
84.9688	AlC2H2S	1.05	38925054
86.9845	AlC2H4S	1.60	1805828
88.0101	AlC2H5O2	1.76	4026814
92.9860	Al2C3H3	-0.04	45036537
93.9429	AlC1H0SNa	0.74	6382475

94.9507	AlC1H1SNa	0.47	1483804
97.9765	AlC3H3S	-0.37	1425737
98.9843	AlC3H4S	-0.62	1.03E+09
100.0099	AlC3H5O2	-0.45	236659.2
100.9636	AlC2H2O1S	-0.26	13546744
100.9999	AlC3H6S	-1.10	18075501
102.9702	Al2C4H1	-1.50	19624895
102.9792	AlC2H4O1S	-0.74	11841749
103.9509	AlC1H1O2S	1.77	22507293
106.9505	AlC2H1SNa	-1.45	2.38E+08
106.9918	AlC1H4O4	-1.40	794409.9
107.9729	Al2C3H2O1	-1.80	303119.3
108.9582	AlC1H1Na3	0.71	1819717
108.9808	Al2C3H3O1	-1.09	584703
110.9519	Al2C2H2P	-0.36	425696.8
110.9842	AlC4H4S	-1.45	7753058
110.9964	Al2C3H5O1	-1.52	60246124
111.9920	AlC4H5S	-1.66	587645.2
112.9635	AlC3H2O1S	-1.12	762672.2
112.9998	AlC4H6S	-1.87	20857280
113.9713	AlC3H3O1S	-1.33	1678752
114.9324	AlC0H2PSNa	1.38	851757.3
114.9736	AlC4H1O1Na	0.88	457548.5
114.9736	Al2C2H5S	-1.09	457548.5
114.9792	AlC3H4O1S	-0.66	21678962
115.0155	AlC4H8S	-1.40	1749540
115.9870	AlC3H5O1S	-0.87	1757974
116.9528	Al2C1H3O1S	-1.63	2248955
116.9947	AlC3H6O1S	-1.93	1.58E+08
119.0103	AlC3H8O1S	-2.32	564613.5
119.9820	AlC2H5O2S	-0.13	7048693
120.9451	AlC1H3O1PS	-0.93	46638865
120.9451	AlC0H0O3Na2	-1.43	46638865
120.9807	Al2C4H3O1	-1.81	72485392
121.0075	AlC2H6O4	-0.83	364355.6
121.0149	Al2C3H8Na	0.02	10205716
121.9614	AlC1H3O3S	0.97	289350.4
122.9244	AlC0H1O2PS	-0.63	51352812
122.9431	AlC0H2O1SNa2	-0.52	336452.8
122.9454	AlC2H1O1SNa	-1.38	3971441
122.9691	AlC1H4O3S	-0.05	334214.8
122.9817	AlC3H5SNa	-2.07	1868863
122.9842	AlC5H4S	-1.31	316253
124.9611	AlC2H3O1SNa	-0.96	330947.9
124.9998	AlC5H6S	-1.69	9939129
125.0180	AlC5H6O2	1.84	1362597
125.9609	AlC1H2O1Na3	0.30	350137.2
125.9835	Al2C3H4O2	-1.26	469267.7

126.9530	Al2C0H2O3Na	2.42	9692855
126.9687	AlC1H3O1Na3	0.10	1336283
126.9791	AlC4H4O1S	-1.39	11676113
126.9913	Al2C3H5O2	-1.45	1489292
127.0155	AlC5H8S	-1.27	5216081
127.9869	AlC4H5O1S	-1.57	350237.7
128.9584	AlC3H2O2S	-1.09	498388.7
128.9624	Al2C2H4O1P	-0.81	6861240
128.9737	AlC1H3O4Na	-1.50	5228608
128.9947	AlC4H6O1S	-1.75	18087912
129.0069	Al2C3H7O2	-1.81	2278939
129.0310	AlC5H10S	-2.41	1983847
130.9595	Al3C4H2	-1.67	617711.4
130.9740	AlC3H4O2S	-1.46	1883527
131.0104	AlC4H8O1S	-1.34	3219396
131.0644	AlC5H12O2	-2.44	460367.8
131.9818	AlC3H5O2S	-1.64	403501.3
132.9661	AlC4H3SNa	-1.54	464387.4
132.9897	AlC3H6O2S	-1.06	909026.5
133.0074	AlC3H6O4	-1.50	244898.5
133.0259	AlC4H10O1S	-2.45	336002.2
133.9793	AlC2H3O5	1.94	6054933
133.9976	AlC3H7O2S	-0.49	386837.1
134.9454	AlC3H1O1SNa	-1.26	339435
134.9606	AlC2H5O1PS	-1.94	242813.4
134.9606	AlC1H2O3Na2	-2.39	242813.4
134.9633	AlC3H1O3Na	-0.22	46633561
134.9963	Al2C5H5O1	-1.99	950069.7
135.0052	AlC3H8O2S	-2.16	1400266
135.0231	AlC3H8O4	-1.11	1023786
135.9531	AlC3H2O1SNa	-2.17	1079120
135.9560	AlC5H1O1S	1.46	1748503
136.9400	AlC1H3O2PS	-0.93	9.61E+08
136.9400	AlC0H0O4Na2	-1.37	9.61E+08
136.9530	AlC2H1O1Na3	-0.27	380805.2
136.9611	AlC3H3O1SNa	-0.88	1943757
138.9194	AlC0H1O3PS	0.06	371424.8
138.9381	AlC0H2O2SNa2	0.15	44482847
138.9442	Al2C1H3O1PNa	-1.79	922351.9
138.9442	Al4C1H3O1	1.46	922351.9
138.9639	AlC1H4O4S	-0.87	1196701
138.9766	AlC3H5O1SNa	-1.94	1.11E+08
138.9791	AlC5H4O1S	-1.27	345001.4
138.9913	Al2C4H5O2	-1.32	1.78E+08
139.9788	AlC4H3O1Na2	-0.84	216404.9
140.0132	AlC3H8Na3	2.16	5724493
140.9947	AlC5H6O1S	-1.60	19949709
142.0026	AlC5H7O1S	-1.06	206672.9

142.9270	AlC1H2O1PSNa	-1.09	6081072
142.9270	Al3C1H2O1S	2.07	6081072
142.9480	AlC3H2SNa2	-1.74	3.65E+08
142.9626	Al2C4H2O1Na	-1.84	234943.9
142.9740	AlC4H4O2S	-1.34	965047
142.9894	AlC2H5O4Na	-1.01	408913.6
143.0104	AlC5H8O1S	-1.23	9075537
144.9252	AlC0H1O1SNa3	0.63	1897543
144.9476	Al2C2H3O2S	-2.10	593940.7
144.9897	AlC4H6O2S	-0.97	3298703
145.0259	AlC5H10O1S	-2.25	768707
145.9976	AlC4H7O2S	-0.45	3007003
146.9730	Al2C2H6O2P	-0.48	2468248
146.9816	AlC5H5SNa	-2.42	2645583
146.9816	Al2C2H5O4	1.95	2645583
147.0053	AlC4H8O2S	-1.30	369763.4
148.0132	AlC4H9O2S	-0.78	375491.2
148.9610	AlC4H3O1SNa	-1.48	1327318
148.9701	Al3C4H4O1	-1.23	230889.2
148.9756	Al2C5H3O2	-1.57	681702.4
148.9973	AlC5H7SNa	-2.05	298767.3
149.0023	AlC3H6O5	-1.44	301758
149.0208	AlC4H10O2S	-2.29	427801.4
149.9925	AlC3H7O3S	-0.54	1550959
150.9403	AlC3H1O2SNa	-1.23	335764.2
150.9556	AlC2H5O2PS	-1.17	917499.3
150.9556	AlC1H2O4Na2	-1.58	917499.3
150.9766	AlC4H5O1SNa	-1.79	643373.3
151.0180	AlC3H8O5	-1.09	1356173
151.0254	Al2C4H10O1Na	-0.42	848431.4
151.9898	AlC2H5O6	1.28	984010.8
152.0132	AlC4H8Na3	1.99	7298314
152.9349	AlC1H3O3PS	-0.93	210521.7
152.9349	AlC0H0O5Na2	-1.33	210521.7
152.9559	AlC3H3O2SNa	-1.54	57198521
152.9649	Al3C3H4O2	-1.95	210295.1
152.9972	AlC2H6O6	-1.50	7335678
153.9665	AlC5H3O2S	0.87	10926572
154.0289	AlC4H10Na3	2.29	262785.8
154.9415	Al2C3H2O2P	-1.74	3719859
154.9635	AlC3H6PSNa	-0.27	549560.9
154.9635	AlC2H3O2Na3	-0.66	549560.9
156.9452	AlC4H3O1PS	-0.08	7589072
156.9452	AlC3H0O3Na2	-0.47	7589072
156.9452	Al2C1H4O2SNa	-1.91	7589072
156.9572	Al2C3H4O2P	-1.40	1162497
156.9897	AlC5H6O2S	-0.90	3960732
157.0018	Al2C4H7O3	-1.59	1107195

157.9974	AlC5H7O2S	-1.68	256393.2
158.9219	AlC1H2O2PSNa	-1.08	2.42E+08
158.9219	Al3C1H2O2S	1.77	2.42E+08
158.9816	Al2C3H5O4	1.80	215280.7
159.0052	AlC5H8O2S	-1.83	8499332
159.9948	AlC4H5O5	0.69	1574567
160.9200	AlC0H1O2SNa3	-0.14	7080633
160.9585	AlC3H4O1SNa2	-1.95	1188523
160.9788	AlC5H3O3Na	-1.11	5144813
160.9845	AlC4H6O3S	-1.59	589139.6
160.9972	Al2C3H7O4	1.47	1309103
161.0208	AlC5H10O2S	-2.12	729103.7
161.9715	AlC1H4O6Na	-0.30	333151
161.9741	AlC3H3O6	0.89	1026445
161.9925	AlC4H7O3S	-0.50	871369.6
162.9555	AlC3H5O2PS	-1.70	333385.2
162.9555	AlC2H2O4Na2	-2.07	333385.2
162.9555	Al2C5H1O3	2.46	333385.2
162.9581	AlC4H1O4Na	-0.88	3281610
162.9581	Al2C2H5O3S	-2.27	3281610
162.9765	AlC5H5O1SNa	-2.27	7107297
162.9765	Al2C2H5O5	1.66	7107297
163.0179	AlC4H8O5	-1.63	307136.9
163.0364	AlC5H12O2S	-2.40	222701.2
164.0081	AlC4H9O3S	-0.80	65216487
164.0131	AlC5H8Na3	1.24	256229.2
164.9091	AlC1H1O1PSNa2	0.00	1096847
164.9204	Al3C3H1O1P	-1.03	535340.9
164.9299	AlC3H1SNa3	-1.78	3923869
164.9299	Al2C1H4O2PS	2.48	3923869
164.9299	Al2C0H1O4Na2	2.11	3923869
164.9922	AlC5H7O1SNa	-1.94	681496.5
165.0157	AlC4H10O3S	-2.16	222128.2
165.0336	AlC4H10O5	-1.30	272472.1
165.9874	AlC3H7O4S	-0.58	15358073
165.9918	AlC4H6O1Na3	-2.18	325705.7
166.0288	AlC5H10Na3	1.52	1691215
166.9861	Al2C5H5O3	-1.79	18511638
167.0129	AlC3H8O6	-1.08	621934.1
168.0445	AlC5H12Na3	1.80	203211.6
168.9299	AlC1H3O4PS	-0.34	7453073
168.9299	AlC0H0O6Na2	-0.70	7453073
168.9662	AlC2H7O3PS	-0.84	2275727
168.9662	AlC1H4O5Na2	-1.20	2275727
168.9871	AlC4H7O2SNa	-1.98	718864.7
169.0018	Al2C5H7O3	-1.47	552311.5
170.9539	AlC1H4O6S	0.29	416333.3
170.9575	Al2C5H2O2Na	-1.63	317695.7

170.9754	Al3C3H6O3	-2.12	4032082
172.9192	AlC1H0O5PNa	0.61	222871.3
172.9192	Al2C4H0O1SNa	-0.49	222871.3
172.9521	Al2C3H4O3P	-1.36	28891738
173.9952	AlC4H7Na4	2.06	512053.6
174.9380	AlC3H2O2SNa2	-0.45	9780833
174.9678	Al2C3H6O3P	-1.06	4582094
174.9792	AlC2H5O6Na	-0.99	1136232
175.0002	AlC5H8O3S	-1.18	599791.2
175.9859	Al2C3H9PNa2	-2.01	81231184
175.9897	AlC4H5O6	0.54	28119662
176.0082	AlC5H9O3S	-0.18	592535.7
176.9737	AlC5H3O4Na	-1.10	73775708
176.9737	Al2C3H7O3S	-2.37	73775708
176.9894	AlC4H7O4P	1.21	3165429
176.9922	Al2C3H7O5	1.81	4353798
177.0269	AlC5H12SNa2	2.26	247727.6
177.9875	AlC4H7O4S	0.02	311581.9
178.0053	AlC4H7O6	0.25	308664.5
178.0238	AlC5H11O3S	-0.46	1088763
178.9504	AlC3H5O3PS	-1.63	3305728
178.9504	AlC2H2O5Na2	-1.97	3305728
178.9504	Al2C5H1O4	2.16	3305728
178.9685	AlC3H5O5P	0.27	25655778
178.9953	AlC4H8O4S	-0.12	252231.1
179.9793	AlC5H6O2SNa	-1.72	222513
179.9793	Al2C2H6O6	1.84	222513
180.0031	AlC4H9O4S	-0.26	12524063
180.9038	AlC1H1O2PSNa2	-1.19	5478357
180.9038	Al3C1H1O2SNa	1.31	5478357
180.9661	AlC3H7O3PS	-1.34	244735.8
180.9661	AlC2H4O5Na2	-1.67	244735.8
180.9661	Al2C5H3O4	2.41	244735.8
180.9871	AlC5H7O2SNa	-1.85	10108581
181.9614	AlC3H4Na5	1.46	791246.5
181.9823	AlC3H7O5S	-0.61	3714167
182.9608	AlC5H2O3Na2	-0.67	385489.3
182.9608	Al2C3H6O2SNa	-1.91	385489.3
182.9665	AlC4H5O3SNa	-1.09	1010355
183.0028	AlC5H9O2SNa	-1.56	1112521
184.9402	AlC5H3O2PS	0.39	808795.7
184.9402	AlC4H0O4Na2	0.06	808795.7
184.9402	Al2C2H4O3SNa	-1.16	808795.7
184.9695	AlC2H6O6S	-0.01	1219412
184.9968	Al2C5H7O4	-0.89	200464.3
185.9901	AlC4H8O3SNa	-0.40	338661.9
187.9694	AlC3H6O4SNa	-0.21	260819.8
187.9898	AlC5H5O6	1.04	356134.4

187.9898	AlC3H9O1PNa3	2.14	356134.4
188.9244	AlC1H2O3PNa3	-0.28	584388.3
188.9244	Al3C2H5O1PS	2.43	584388.3
188.9244	Al3C1H2O3Na2	2.11	584388.3
188.9534	AlC4H4O2SNa2	-1.74	26597087
188.9534	Al2C1H4O6Na	1.65	26597087
188.9860	Al3C3H8O4	-1.74	4200750
188.9922	Al2C4H7O5	1.70	483325.3
189.9691	AlC4H3O7	1.21	672256.6
189.9691	AlC2H7O2PNa3	2.30	672256.6
189.9874	AlC5H7O4S	-0.51	214521.3
190.0019	Al2C4H11PNa2	-0.02	220589.4
190.9119	AlC1H2O4PSNa	-0.01	3393459
190.9119	Al3C1H2O4S	2.36	3393459
190.9531	AlC5H1O5Na	-0.31	973200.2
190.9531	AlC3H5PNa4	0.77	973200.2
190.9531	Al2C3H5O4S	-1.49	973200.2
190.9626	Al2C3H6O4P	-1.57	1.95E+08
191.0078	Al2C4H9O5	1.42	215168.9
191.9671	AlC4H5O5S	1.77	494836.1
192.0031	AlC5H9O4S	-0.24	2376921
192.9664	AlC4H7O3PS	0.30	596733.6
192.9664	AlC3H4O5Na2	-0.02	596733.6
192.9872	Al2C3H7O6	2.10	3344313
193.9825	AlC4H7O5S	0.46	437594.5
194.0003	AlC4H7O7	0.67	14349601
194.0188	AlC5H11O4S	0.02	1814692
194.9305	AlC4H1O4SNa	0.95	4416318
194.9341	Al2C3H3O3PNa	-0.92	1921204
194.9341	Al4C3H3O3	1.40	1921204
194.9425	Al2C3H2O5Na	-0.12	3718581
194.9556	AlC3H7O1PSNa2	-2.31	410371.1
194.9556	Al2C5H3O2Na2	1.17	410371.1
194.9556	Al3C3H7O1SNa	0.01	410371.1
194.9661	Al2C2H5O7	0.21	395643.7
195.9770	AlC4H6Na5	1.10	21083862
195.9981	AlC4H9O5S	0.20	1349652
196.9610	AlC3H7O4PS	-1.31	7330561
196.9610	AlC2H4O6Na2	-1.62	7330561
196.9610	Al2C5H3O5	2.14	7330561
196.9821	AlC5H7O3SNa	-1.27	1601472
197.9679	Al2C3H8PNa3	-1.50	363930.8
197.9679	Al4C3H8Na2	0.78	363930.8
197.9717	AlC4H4O6Na	0.76	4443570
197.9926	AlC4H8Na5	0.83	1969123
198.0011	AlC5H10SNa3	2.43	2001669
198.9557	AlC5H2O4Na2	-0.69	17208708
198.9557	Al2C3H6O3SNa	-1.83	17208708

198.9647	Al4C3H7O3	-2.15	475291
198.9704	Al3C4H6O4	-1.40	1212382
198.9851	AlC3H8O6S	-0.26	3219702
199.0002	AlC4H9Na5	-0.30	507826.4
199.0085	AlC5H11SNa3	0.29	262248.8
200.0081	AlC4H10Na5	0.07	258888.2
200.9066	AlC2H2O1PSNa3	-0.47	227568.2
200.9066	Al3C2H2O1SNa2	1.78	227568.2
200.9323	AlC3H4O3PSNa	-1.67	12129428
200.9323	AlC2H1O5Na3	-1.98	12129428
200.9323	Al2C5H0O4Na	1.70	12129428
200.9323	Al3C3H4O3S	0.58	12129428
200.9470	Al2C4H4O4P	-1.24	263683.8
200.9922	Al2C5H7O5	1.60	3126233
201.9960	AlC4H10O1SNa3	2.31	52395981
202.0054	AlC4H11O1PNa3	1.74	842569.8
202.9480	AlC3H6O3PSNa	-1.41	5565498
202.9480	AlC2H3O5Na3	-1.71	5565498
202.9480	Al2C5H2O4Na	1.93	5565498
202.9480	Al3C3H6O3S	0.82	5565498
202.9626	Al2C4H6O4P	-1.48	217672.9
202.9691	AlC5H6O2SNa2	-1.38	298277.8
202.9691	Al2C2H6O6Na	1.78	298277.8
203.0006	Al2C5H11SNa2	1.91	344747.9
203.9809	Al2C4H9O1PNa2	-1.31	3279734
203.9809	Al4C4H9O1Na	0.90	3279734
203.9847	AlC5H5O7	0.88	5013602
203.9847	AlC3H9O2PNa3	1.90	5013602
204.0178	Al2C5H13PNa2	1.21	303479.6
204.9275	AlC2H4O4PSNa	-0.25	27435229
204.9275	AlC1H1O6Na3	-0.55	27435229
204.9275	Al3C2H4O4S	1.96	27435229
204.9687	AlC4H7PNa4	0.48	4492913
204.9687	Al2C4H7O4S	-1.63	4492913
204.9870	Al2C4H7O6	1.00	510500.2
205.9978	AlC3H8O7Na	0.17	434189.5
206.9256	AlC1H3O4SNa3	0.47	202958.9
206.9256	Al4C4H4O1P	0.08	202958.9
206.9515	AlC2H5O6SNa	0.27	355842.7
206.9635	AlC4H5O6P	0.65	3678583
207.0028	Al2C4H9O6	1.72	965527.4
207.9981	AlC5H9O5S	0.19	3674967
208.9733	Al2C3H8O5P	-0.79	1042167
208.9821	Al2C3H7O7	1.87	1682443
210.0136	AlC5H11O5S	-0.53	725445.1
210.9080	Al3C4H3O1PS	-1.38	221932.3
210.9080	Al3C3H0O3Na2	-1.66	221932.3
210.9259	Al3C4H3O3P	-0.71	254263.3

210.9259	Al4C1H4O4Na	-2.07	254263.3
210.9355	AlC4H3O2SNa3	-0.82	5379813
210.9355	Al2C1H3O6Na2	2.22	5379813
210.9768	AlC4H9O4PS	-0.51	766747.7
210.9768	AlC3H6O6Na2	-0.80	766747.7
210.9852	AlC4H8O6S	0.23	289897
210.9878	AlC4H11O1PSNa2	2.13	157113.5
210.9878	AlC3H8O3Na4	1.84	157113.5
211.9926	AlC4H9O6S	-1.77	411911.3
211.9926	Al2C4H9O2Na3	2.40	411911.3
212.9316	Al2C1H4O7P	-1.86	157312.3
212.9351	AlC5H0O5Na2	-0.01	224754.4
212.9351	AlC3H4PNa5	0.96	224754.4
212.9351	Al2C3H4O4SNa	-1.08	224754.4
212.9411	AlC4H3O5SNa	1.03	412553.8
212.9411	AlC2H7PSNa4	2.00	412553.8
212.9446	Al2C3H5O4PNa	-1.15	299516
212.9446	Al4C3H5O4	0.98	299516
212.9767	Al2C5H7Na4	1.50	184767.1
213.0008	AlC4H10O6S	-0.01	3004609
213.0158	AlC5H11Na5	-0.52	1908913
213.9456	AlC2H4O8P	1.07	327425.2
213.9456	AlC5H4PNa4	2.21	327425.2
213.9456	Al2C5H4O4S	0.19	327425.2
213.9456	Al2C2H5O1Na5	0.87	327425.2
213.9875	AlC4H8O1Na5	0.70	38114462
213.9960	AlC5H10O1SNa3	2.18	503114.8
214.9296	AlC3H2O6PNa	-0.28	1996508
214.9296	Al3C3H2O6	1.82	1996508
214.9627	Al2C5H6O4P	-0.93	291475.9
214.9651	Al3C4H6O5	-2.29	2392875
214.9801	AlC3H8O7S	0.16	194754.7
215.9697	AlC2H5O10	2.01	553193.6
216.0117	AlC5H12O1SNa3	2.39	2085911
216.9064	AlC0H1O7PNa2	-0.55	2865109
216.9064	Al2C3H1O3SNa2	-1.42	2865109
216.9064	Al3C0H1O7Na	1.53	2865109
216.9538	AlC3H3O8Na	1.13	957914.1
216.9658	AlC3H8PNa5	-1.83	27694609
216.9658	Al3C3H8Na4	0.26	27694609
216.9848	Al2C3H8O6Na	1.90	179741.6
216.9871	Al2C5H7O6	1.41	539948.8
217.9736	AlC3H9PNa5	-1.93	6048873
218.0003	AlC4H11O2PNa3	1.55	288041.4
218.9220	AlC0H3O7PNa2	-0.77	269391.1
218.9220	Al2C3H3O3SNa2	-1.64	269391.1
218.9429	AlC3H6O4PSNa	-1.38	82403323
218.9429	AlC2H3O6Na3	-1.65	82403323

218.9429	Al2C5H2O5Na	1.72	82403323
218.9429	Al3C3H6O4S	0.69	82403323
218.9576	Al2C4H6O5P	-0.98	16397360
218.9611	AlC3H6O6PNa	0.64	157274.5
218.9695	AlC3H5O8Na	1.35	153421.5
219.0028	Al2C5H9O6	1.63	6901285
220.0134	AlC4H10O7Na	-0.06	1324515
220.9015	AlC5H0O3PSNa	0.74	408809.6
220.9015	Al2C2H1O4SNa2	-0.56	408809.6
220.9377	AlC5H1O4Na3	-0.37	1206906
220.9377	Al2C3H5O3SNa2	-1.39	1206906
220.9581	Al2C5H4O5Na	-0.33	481984.1
220.9581	Al2C3H8PNa4	0.61	481984.1
220.9581	Al3C3H8O4S	-1.35	481984.1
221.0185	Al2C5H11O6	1.84	210400.7
221.9926	AlC3H8O8Na	-0.36	1359095
222.9143	AlC3H3O3PSNa2	-1.26	1168654
222.9143	AlC2H0O5Na4	-1.53	1168654
222.9143	Al2C0H3O7PNa	1.62	1168654
222.9143	Al3C3H3O3SNa	0.77	1168654
222.9254	AlC5H1O5SNa	0.76	515211.8
222.9254	AlC3H5PSNa4	1.69	515211.8
222.9290	Al2C4H3O4PNa	-0.87	1034111
222.9290	Al3C2H7O3PS	-1.88	1034111
222.9290	Al3C1H4O5Na2	-2.16	1034111
222.9290	Al4C4H3O4	1.16	1034111
222.9374	Al2C4H2O6Na	-0.17	265909.9
222.9374	Al2C2H6O1PNa4	0.76	265909.9
222.9374	Al3C2H6O5S	-1.18	265909.9
222.9977	Al2C4H9O7	1.53	1207933
223.9721	AlC2H6O9Na	0.70	185002.2
223.9721	AlC5H6O1Na5	1.79	185002.2
223.9780	AlC4H9O1SNa4	2.33	692225.7
223.9930	AlC5H9O6S	0.11	158044
224.9299	AlC3H5O3PSNa2	-1.47	314262.3
224.9299	AlC2H2O5Na4	-1.74	314262.3
224.9299	Al2C5H1O4Na2	1.55	314262.3
224.9299	Al3C3H5O3SNa	0.54	314262.3
225.0008	AlC5H10O6S	0.00	541176.7
225.9629	Al2C4H8O1PNa3	-0.94	165707.8
225.9629	Al4C4H8O1Na2	1.06	165707.8
225.9666	AlC5H4O7Na	0.60	948158.7
225.9666	AlC3H8O2PNa4	1.52	948158.7
225.9666	Al2C3H8O6S	-0.40	948158.7
226.0083	AlC5H11O6S	-1.44	268518
226.0083	Al2C5H11O2Na3	2.47	268518
226.8999	Al4C4H0O3Na	-0.97	231628.2
226.8999	Al5C2H4O2S	-1.97	231628.2

226.9094	AlC2H3O4PSNa2	-0.42	6551928
226.9094	AlC1H0O6Na4	-0.69	6551928
226.9094	Al3C2H3O4SNa	1.57	6551928
226.9094	Al5C2H5O2P	-2.04	6551928
226.9506	AlC4H6PNa5	0.24	973972.6
226.9506	Al2C4H6O4SNa	-1.67	973972.6
226.9506	Al3C4H6Na4	2.23	973972.6
226.9595	Al4C4H7O4	-2.39	196182.2
226.9801	AlC4H8O7S	0.15	251152.6
227.0164	AlC5H12O6S	-0.23	306744.1
228.9958	AlC4H10O7S	0.37	2694951
229.9910	AlC5H10O2SNa3	2.40	2780933
229.9910	Al3C5H12PNa2	-1.16	2780933
229.9910	Al5C5H12Na	0.80	2780933
230.9427	AlC4H6O4PSNa	-2.17	427926.6
230.9427	AlC3H3O6Na3	-2.43	427926.6
230.9427	Al2C4H6PNa4	1.66	427926.6
230.9427	Al3C4H6O4S	-0.21	427926.6
230.9547	Al4C3H7O5	-1.11	226034.2
230.9750	AlC3H8O8S	0.08	1087652
232.9586	AlC4H8O4PSNa	-1.08	278276.9
232.9586	AlC3H5O6Na3	-1.34	278276.9
232.9586	Al3C4H8O4S	0.86	278276.9
232.9732	Al2C5H8O5P	-1.14	258028
233.9924	AlC4H8O8Na	-1.19	3640362
234.9169	AlC0H3O8PNa2	-0.78	14737950
234.9169	Al2C3H2O7P	2.36	14737950
234.9169	Al2C3H3O4SNa2	-1.59	14737950
234.9169	Al3C3H3Na5	2.18	14737950
234.9643	AlC3H5O9Na	0.77	10483250
234.9643	Al2C4H9SNa4	0.85	10483250
234.9764	AlC5H6O6Na2	-2.42	26164263
234.9977	Al2C5H9O7	1.45	3185671
235.9569	AlC5H5O7S	1.31	8138461
235.9569	AlC3H9O2PSNa3	2.19	8138461
235.9569	AlC2H6O4Na5	1.93	8138461
235.9600	Al2C4H7O6P	-2.35	1469925
235.9689	Al2C4H6O8	0.43	3318309
236.0083	AlC4H10O8Na	-0.12	479256.7
236.9116	AlC3H1O6PNa2	-0.02	399512.7
236.9116	Al2C1H5O5PSNa	-0.97	399512.7
236.9116	Al2C0H2O7Na3	-1.23	399512.7
236.9116	Al3C3H1O6Na	1.89	399512.7
236.9409	AlC4H7PSNa4	0.96	3173562
236.9529	Al2C5H4O6Na	-0.79	19442180
236.9529	Al2C3H8O1PNa4	0.08	19442180
236.9529	Al3C3H8O5S	-1.75	19442180
236.9529	Al4C3H8O1Na3	1.99	19442180

236.9618	AlC3H7O7SNa	-0.89	203672.9
237.0133	Al2C5H11O7	1.23	1755087
237.9725	AlC5H7O7S	1.09	609747.4
237.9876	AlC3H8O9Na	0.02	303381.7
238.8882	AlC0H0O7PNa3	-1.10	665093.1
238.8882	Al2C3H0O3SNa3	-1.89	665093.1
238.8882	Al3C1H3O5PS	1.05	665093.1
238.8882	Al3C0H0O7Na2	0.79	665093.1
238.9565	AlC4H9PSNa4	0.74	986171.9
238.9926	Al2C4H9O8	1.36	224353.7
240.9039	AlC0H2O7PNa3	-0.88	157247.4
240.9039	Al2C3H1O6PNa	2.18	157247.4
240.9039	Al2C3H2O3SNa3	-1.67	157247.4
240.9039	Al3C1H5O5PS	1.24	157247.4
240.9039	Al3C0H2O7Na2	0.99	157247.4
240.9154	Al4C5H2O3Na	-1.54	150340.2
240.9154	Al5C3H6O2S	-2.48	150340.2
240.9249	AlC3H5O4PSNa2	-1.02	21469298
240.9249	AlC2H2O6Na4	-1.27	21469298
240.9249	Al2C5H1O5Na2	1.80	21469298
240.9249	Al3C3H5O4SNa	0.86	21469298
240.9360	AlC5H3O6SNa	0.85	295342.4
240.9360	AlC3H7O1PSNa4	1.71	295342.4
240.9395	Al2C4H5O5PNa	-1.08	87542.09
240.9395	Al4C4H5O5	0.80	87542.09
240.9847	Al2C5H8O6Na	1.29	81056.74
240.9957	AlC5H10O7S	-0.07	758770.6
242.8834	Al2C2H0O4SNa3	-0.69	1542091
242.8834	Al3C0H3O6PS	2.20	1542091
242.8834	Al5C5H1O1P	-1.02	1542091
242.9254	AlC4H3O4SNa3	-0.42	383154.3
242.9254	Al2C2H6O6PS	2.47	383154.3
242.9254	Al2C1H3O8Na2	2.21	383154.3
242.9254	Al5C4H5O2Na	-1.94	383154.3
242.9600	AlC3H8O3SNa4	2.13	555024.9
242.9600	Al3C5H6O6	-2.09	555024.9
243.0114	AlC5H12O7S	0.14	412408.7
243.9646	Al2C4H9O2SNa3	1.79	167525.1
243.9646	Al4C4H11PNa2	-1.56	167525.1
243.9746	AlC4H10O6PS	0.16	257010.1
243.9746	AlC3H7O8Na2	-0.09	257010.1
243.9892	AlC5H11PNa5	-1.93	205078.1
243.9892	Al3C5H11Na4	-0.08	205078.1
244.9486	AlC4H3O9Na	0.53	196867.3
244.9486	AlC2H7O4PNa4	1.38	196867.3
244.9486	Al2C5H7SNa4	0.61	196867.3
244.9581	Al2C5H8PNa4	0.55	110072.7
244.9581	Al3C5H8O4S	-1.22	110072.7

244.9581	Al4C5H8Na3	2.39	110072.7
244.9607	AlC4H8O1PNa5	-1.68	177393.9
244.9607	Al3C4H8O1Na4	0.17	177393.9
244.9907	AlC4H10O8S	0.28	337855.2
245.9685	AlC4H9O1PNa5	-1.77	207360.7
245.9685	Al3C4H9O1Na4	0.06	207360.7
246.9373	Al2C4H6O1PNa4	0.28	1263577
246.9373	Al3C4H6O5S	-1.47	1263577
246.9373	Al4C4H6O1Na3	2.11	1263577
246.9525	Al2C5H6O6P	-0.93	187062.4
248.9325	AlC1H5O8PNa2	-0.94	111921
248.9325	Al2C4H4O7P	2.03	111921
248.9325	Al2C4H5O4SNa2	-1.70	111921
248.9325	Al3C4H5Na5	1.86	111921
249.9634	AlC3H9O2PNa5	-1.80	131596
250.0237	AlC5H12O8Na	-1.12	86603.16
250.9418	AlC3H5O8SNa	2.09	1168424
250.9418	Al3C3H7O6P	-1.17	1168424
250.9717	AlC5H6O7Na2	-0.73	358510.6
250.9924	Al2C5H9O8	0.50	98654.34
251.9343	Al3C4H4O6Na	-1.30	937157.5
251.9343	Al4C5H7PNa2	2.46	937157.5
251.9435	AlC5H6O6PS	0.94	309137.8
251.9435	AlC4H3O8Na2	0.70	309137.8
251.9435	AlC2H7O3PNa5	1.52	309137.8
251.9729	AlC5H9O2SNa4	2.02	314915.7
251.9729	Al3C5H11PNa3	-1.23	314915.7
251.9729	Al5C5H11Na2	0.56	314915.7
252.0032	AlC4H10O9Na	-0.17	120817.7
252.9184	Al3C5H2O4Na2	-2.04	1743467
252.9569	AlC3H7O8SNa	-0.10	194235.1
252.9871	AlC5H8O7Na2	-1.71	263970.7
253.0082	Al2C5H11O8	1.09	1145638
253.9579	Al2C5H8O2PNa3	-0.50	79687.29
253.9579	Al4C5H8O2Na2	1.28	79687.29
253.9674	AlC5H7O8S	0.96	25361086
253.9795	AlC5H12PSNa4	-1.17	127593.4
253.9795	Al2C4H8O9	0.54	127593.4
253.9795	Al3C5H12SNa3	0.61	127593.4
254.9514	AlC4H9O1PSNa4	0.64	42955096
254.9514	Al2C3H5O10	2.34	42955096
254.9514	Al3C4H9O1SNa3	2.41	42955096
254.9683	Al3C5H12O1PSNa	-0.72	500836.4
254.9683	Al3C4H9O3Na3	-0.96	500836.4
254.9683	Al5C5H12O1S	1.05	500836.4
254.9877	AlC5H13PSNa4	0.30	326702.9
254.9877	Al2C4H9O9	2.00	326702.9
255.9523	Al3C5H7O1Na4	-2.09	1099543

255.9560	AlC4H6O9P	0.25	892193.2
255.9560	Al2C5H10PSNa3	0.32	892193.2
255.9560	Al2C4H7O2Na5	0.08	892193.2
255.9560	Al4C5H10SNa2	2.09	892193.2
255.9650	Al4C5H11PNa2	0.07	665123.4
256.8894	AlC0H1O8SNa3	-0.44	394727.2
256.8894	Al2C3H0O7SNa	2.44	394727.2
256.8894	Al4C3H2O5P	-0.75	394727.2
256.8988	AlC0H2O8PNa3	-0.89	16811455
256.8988	Al2C3H1O7PNa	1.99	16811455
256.8988	Al2C3H2O4SNa3	-1.62	16811455
256.8988	Al3C1H5O6PS	1.11	16811455
256.8988	Al3C0H2O8Na2	0.87	16811455
256.9401	AlC5H4O7PNa	-0.49	949634.9
256.9401	Al2C3H8O6PS	-1.37	949634.9
256.9401	Al2C2H5O8Na2	-1.60	949634.9
256.9401	Al3C5H4O7	1.27	949634.9
256.9401	Al3C3H8O2PNa3	2.08	949634.9
256.9461	AlC4H7O7PS	0.38	263046.7
256.9461	AlC3H4O9Na2	0.14	263046.7
256.9461	Al2C4H8SNa5	0.21	263046.7
256.9490	AlC5H3O9Na	2.07	1387935
256.9490	Al2C3H7O8S	1.19	1387935
256.9583	AlC5H5O6Na3	-2.38	4085054
256.9583	Al2C3H8O8P	0.35	4085054
256.9906	AlC5H10O8S	-0.12	150139.4
257.9388	AlC5H4O7SNa	1.03	153971.3
257.9388	AlC3H8O2PSNa4	1.83	153971.3
257.9388	Al3C5H6O5P	-2.15	153971.3
257.9419	Al2C4H6O6PNa	-2.32	267531.3
257.9419	Al4C4H6O6	-0.57	267531.3
257.9508	Al2C4H5O8Na	0.22	91188.44
258.8994	AlC2H3O6PSNa2	0.29	128975.6
258.8994	AlC1H0O8Na4	0.05	128975.6
258.8994	Al3C2H3O6SNa	2.04	128975.6
258.8994	Al5C2H5O4P	-1.13	128975.6
258.9229	AlC4H6PSNa5	1.09	1691034
258.9349	Al2C5H3O6Na2	-0.51	2623984
258.9349	Al2C3H7O1PNa5	0.29	2623984
258.9349	Al3C3H7O5SNa	-1.38	2623984
258.9349	Al4C3H7O1Na4	2.03	2623984
258.9376	AlC5H6O5PSNa	-1.99	108191.3
258.9376	AlC4H3O7Na3	-2.23	108191.3
258.9376	Al2C2H6O9P	0.48	108191.3
258.9376	Al2C5H6O1PNa4	1.42	108191.3
258.9376	Al3C5H6O5S	-0.25	108191.3
259.0063	AlC5H12O8S	0.07	95074.44
259.9694	AlC4H10O7PS	-0.30	83883.19

259.9694	AlC3H7O9Na2	-0.53	83883.19
260.9383	AlC4H8PSNa5	0.13	113823.5
260.9383	Al2C3H4O9Na	1.79	113823.5
260.9383	Al3C4H8SNa4	1.86	113823.5
260.9501	Al2C5H5O6Na2	-2.23	118733.1
260.9501	Al2C3H9O1PNa5	-1.44	118733.1
260.9533	AlC5H8O5PSNa	-1.79	118388.3
260.9533	AlC4H5O7Na3	-2.02	118388.3
260.9533	Al2C5H8O1PNa4	1.61	118388.3
260.9533	Al3C5H8O5S	-0.05	118388.3
260.9826	AlC5H10O4PNa3	2.42	142857.3
260.9826	AlC5H11O1SNa5	-1.13	142857.3
261.0112	Al2C5H12O7Na	2.29	95032.67
262.8974	Al4C5H1O3Na2	-1.20	193950.1
262.8974	Al5C3H5O2SNa	-2.06	193950.1
262.9069	AlC5H0O9S	-1.51	2197015
262.9069	AlC3H4O4PSNa3	-0.72	2197015
262.9069	AlC2H1O6Na5	-0.95	2197015
262.9069	Al2C5H0O5Na3	1.86	2197015
262.9069	Al3C3H4O4SNa2	1.00	2197015
262.9069	Al5C3H6O2PNa	-2.12	2197015
262.9267	AlC5H3O6PNa2	-2.11	366914.9
262.9267	Al3C5H3O6Na	-0.39	366914.9
262.9267	Al3C3H7O1PNa4	0.40	366914.9
262.9267	Al4C3H7O5S	-1.25	366914.9
262.9267	Al5C3H7O1Na3	2.12	366914.9
262.9713	AlC4H10O2PNa5	-1.43	3668021
262.9713	Al3C5H13PSNa2	0.52	3668021
262.9713	Al3C4H10O2Na4	0.29	3668021
263.9518	AlC4H9O3PSNa3	1.90	261405.9
263.9518	AlC3H6O5Na5	1.67	261405.9
263.9518	Al5C4H11O1PNa	0.51	261405.9
263.9638	Al2C5H6O9	0.33	544312.1
264.9359	AlC2H7O9PS	0.26	91933.54
264.9359	AlC5H7O1PSNa4	1.18	91933.54
265.9675	AlC4H11O3PSNa3	2.07	87537.64
265.9675	AlC3H8O5Na5	1.84	87537.64
266.0189	AlC5H12O9Na	0.02	90370.92
266.9426	Al2C4H6O8P	0.15	162368.7
266.9426	Al3C4H7O1Na5	-0.01	162368.7
267.9982	AlC4H10O10Na	0.15	98882.94
268.9193	Al2C1H5O9PNa	-0.44	119395.6
268.9193	Al2C4H5O1PNa5	0.46	119395.6
268.9193	Al3C4H5O5SNa	-1.15	119395.6
268.9193	Al4C4H5O1Na4	2.15	119395.6
268.9823	AlC5H8O8Na2	-0.55	112295.2
269.9450	AlC4H6O7PNa2	-2.39	1335299
269.9450	Al3C4H6O7Na	-0.71	1335299

270.9290	Al3C5H4O5Na2	-1.78	4087413
270.9290	Al3C3H8PNa5	-1.01	4087413
270.9290	Al5C3H8Na4	0.66	4087413
270.9889	Al3C5H11O6Na	-1.85	91869.5
272.8728	Al2C1H2O6PSNa2	-0.88	1140823
272.8728	Al3C4H1O5PS	1.83	1140823
272.8728	Al3C1H2O2PNa5	2.37	1140823
272.8728	Al4C1H2O6SNa	0.78	1140823
272.9322	AlC3H5O8PNa2	-1.96	283758.5
272.9322	Al3C3H5O8Na	-0.30	283758.5
272.9532	AlC5H5O7Na3	-2.30	112915.7
272.9532	Al2C3H8O9P	0.27	112915.7
273.9128	AlC3H4O9PS	1.62	66484.49
273.9128	Al2C3H5O2SNa5	1.46	66484.49
273.9128	Al4C5H3O5Na	-2.29	66484.49
273.9128	Al4C3H7PNa4	-1.53	66484.49
273.9160	Al3C5H6O4PS	-1.86	64983.72
273.9160	Al3C4H3O6Na2	-2.08	64983.72
273.9160	Al3C2H7O1PNa5	-1.33	64983.72
273.9160	Al4C5H6PNa3	1.37	64983.72
274.8968	AlC4H2O7PSNa	0.56	715246
274.8968	Al2C1H3O8SNa2	-0.48	715246
274.8968	Al3C4H2O7S	2.20	715246
274.9003	Al3C5H1O4Na3	-2.04	711167.5
274.9003	Al4C3H4O6P	0.52	711167.5
274.9088	Al2C3H3O8PNa	-0.20	165691.5
274.9088	Al4C3H3O8	1.45	165691.5
274.9563	AlC4H9O8PS	-0.97	1079764
274.9563	AlC3H6O10Na2	-1.19	1079764
274.9563	Al2C4H9O4PNa3	2.25	1079764
274.9563	Al2C4H10O1SNa5	-1.13	1079764
274.9691	AlC5H7O7Na3	-1.37	106611.1
275.9494	AlC5H6O8SNa	1.09	481599.2
275.9494	Al3C5H8O6P	-1.88	481599.2
276.9209	AlC4H3O9SNa	1.30	546549.7
276.9209	AlC2H7O4PSNa4	2.05	546549.7
276.9209	Al3C4H5O7P	-1.66	546549.7
276.9209	Al4C4H6Na5	-1.81	546549.7
276.9333	AlC4H8O1PSNa5	0.43	9174757
276.9333	Al2C3H4O10Na	1.99	9174757
276.9333	Al3C4H8O1SNa4	2.06	9174757
276.9450	Al2C5H5O7Na2	-2.16	27933159
276.9450	Al2C3H9O2PNa5	-1.41	27933159
276.9849	AlC5H9O7Na3	-0.82	91809.74
276.9960	AlC5H11O4Na5	1.55	63483.47
277.9342	Al2C5H5O8P	-1.93	121998
277.9342	Al3C5H6O1Na5	-2.08	121998
277.9342	Al4C3H9O3PNa2	0.45	121998

277.9380	AlC4H5O9PNa	0.43	133691.7
277.9380	Al2C5H9PSNa4	0.50	133691.7
277.9380	Al3C4H5O9	2.06	133691.7
277.9380	Al4C5H9SNa3	2.12	133691.7
277.9590	AlC4H9O3PNa5	0.84	171707.7
277.9590	Al2C4H9O7SNa	-0.72	171707.7
277.9590	Al3C4H9O3Na4	2.47	171707.7
277.9675	AlC5H11O3PSNa3	1.98	132733.2
277.9675	AlC4H8O5Na5	1.76	132733.2
277.9675	Al5C5H13O1PNa	0.66	132733.2
278.0031	AlC5H12O4Na5	-1.06	96793.18
278.8714	AlC0H0O8SNa4	-0.20	306374.5
278.8714	Al4C3H1O5PNa	-0.49	306374.5
278.8714	Al5C1H5O4PS	-1.30	306374.5
278.8714	Al5C0H2O6Na2	-1.52	306374.5
278.8809	AlC0H1O8PNa4	-0.26	3117327
278.8809	Al2C3H0O7PNa2	2.39	3117327
278.8809	Al2C3H1O4SNa4	-0.94	3117327
278.8809	Al3C1H4O6PSNa	1.58	3117327
278.8809	Al3C0H1O8Na3	1.36	3117327
278.9216	AlC5H3O7PNa2	-2.04	11926003
278.9216	Al3C5H3O7Na	-0.42	11926003
278.9216	Al3C3H7O2PNa4	0.32	11926003
278.9216	Al4C3H7O6S	-1.23	11926003
278.9216	Al5C4H10PSNa	2.16	11926003
278.9216	Al5C3H7O2Na3	1.94	11926003
278.9309	AlC5H2O9Na2	1.75	126108
278.9309	AlC3H6O4PNa5	2.49	126108
278.9309	Al2C3H6O8SNa	0.94	126108
278.9309	Al4C3H8O6P	-2.00	126108
278.9367	AlC4H5O9SNa	1.83	56486.09
278.9367	Al3C4H7O7P	-1.11	56486.09
278.9367	Al4C4H8Na5	-1.26	56486.09
278.9515	AlC3H9O9PS	0.06	84493.61
278.9515	Al2C5H5O10	2.49	84493.61
279.9831	AlC5H13O3PSNa3	1.79	1236602
279.9831	AlC4H10O5Na5	1.57	1236602
279.9980	AlC5H10O10Na	-0.57	65828.37
280.8983	Al2C5H1O7PNa	0.04	68060.37
280.8983	Al3C3H5O6PS	-0.77	68060.37
280.8983	Al3C2H2O8Na2	-0.98	68060.37
280.8983	Al4C5H1O7	1.65	68060.37
280.8983	Al4C3H5O2PNa3	2.38	68060.37
280.9168	Al2C5H2O6Na3	-0.63	60823.6
280.9168	Al3C3H5O8P	1.87	60823.6
280.9168	Al3C3H6O5SNa2	-1.43	60823.6
280.9168	Al4C3H6O1Na5	1.72	60823.6
280.9784	Al2C5H12O2PNa4	-2.48	129122.3

280.9784	Al4C5H12O2Na3	-0.87	129122.3
281.9624	AlC4H11O4PSNa3	1.90	2383786
281.9624	AlC3H8O6Na5	1.68	2383786
282.0138	AlC5H12O10Na	-0.03	79468.36
282.9465	AlC5H9O2PSNa4	1.23	450991.8
282.9465	Al5C5H11PNa2	-0.07	450991.8
282.9632	Al3C5H9O4Na3	-0.92	49861.41
283.9510	AlC5H6O10P	0.52	72857.89
283.9510	Al2C5H7O3Na5	0.37	72857.89
283.9670	AlC4H11O7PSNa	-0.25	74915.5
283.9670	AlC3H8O9Na3	-0.47	74915.5
286.9178	AlC2H6O9PSNa	0.08	53278.47
286.9178	AlC5H6O1PSNa5	0.93	53278.47
286.9178	Al2C4H2O10Na	2.45	53278.47
286.9178	Al3C2H6O9S	1.66	53278.47
288.0008	AlC5H11O9Na2	-0.13	69901.52
289.9797	AlC5H12O8PS	-1.18	63095.69
289.9797	AlC4H9O10Na2	-1.39	63095.69
289.9797	Al2C5H12O4PNa3	1.87	63095.69
290.8743	Al3C3H1O6PNa2	-1.31	99767.97
290.8743	Al4C1H5O5PSNa	-2.09	99767.97
290.8743	Al4C0H2O7Na3	-2.30	99767.97
290.8743	Al5C3H1O6Na	0.24	99767.97
290.8743	Al5C1H5O1PNa4	0.95	99767.97
290.9338	AlC3H6O9SNa2	0.05	99248.22
290.9338	Al5C3H8O7	-1.21	99248.22
291.9320	Al2C5H4O8Na2	-2.35	533387.7
291.9320	Al2C3H8O3PNa5	-1.64	533387.7
291.9320	Al4C4H11O1PSNa2	0.11	533387.7
291.9320	Al4C3H8O3Na4	-0.10	533387.7
291.9831	AlC5H10O5Na5	1.51	70681.67
292.8984	AlC3H2O8PNa3	-2.14	123867.8
292.8984	Al3C4H5O6PS	-0.39	123867.8
292.8984	Al3C3H2O8Na2	-0.60	123867.8
292.9074	AlC4H4O8PSNa	0.64	245499.1
292.9074	AlC3H1O10Na3	0.44	245499.1
292.9074	Al3C4H4O8S	2.19	245499.1
292.9108	Al3C5H3O5Na3	-2.13	143183.3
292.9108	Al4C3H6O7P	0.26	143183.3
292.9108	Al5C3H7Na5	0.12	143183.3
292.9671	AlC4H11O9PS	-0.11	71471.83
293.9539	AlC4H9O4PNa5	0.74	65460.17
293.9539	Al2C4H9O8SNa	-0.73	65460.17
293.9539	Al3C5H12O2PSNa2	2.49	65460.17
293.9539	Al3C4H9O4Na4	2.28	65460.17
293.9715	Al2C4H9O10Na	-1.27	124867.2
293.9715	Al3C5H12O4PNa2	1.95	124867.2
293.9977	AlC5H12O5Na5	-2.08	113577.5

294.8453	Al5C4H1O3PS	-0.84	77797.71
294.8453	Al5C1H2PNa5	-0.35	77797.71
294.8549	Al2C1H1O6PSNa3	-0.28	648768.6
294.8549	Al3C4H0O5PSNa	2.22	648768.6
294.8549	Al4C1H1O6SNa2	1.25	648768.6
294.8968	AlC3H4O6PSNa3	-0.40	227368.4
294.8968	AlC2H1O8Na5	-0.61	227368.4
294.8968	Al2C5H0O7Na3	1.90	227368.4
294.8968	Al3C3H4O6SNa2	1.13	227368.4
294.8968	Al5C3H6O4PNa	-1.65	227368.4
294.9315	AlC4H5O10SNa	1.34	1775444
294.9315	Al3C4H7O8P	-1.44	1775444
294.9315	Al4C4H8O1Na5	-1.58	1775444
295.9323	AlC3H7O5PNa5	-2.18	93302.61
295.9323	Al3C4H10O3PSNa2	-0.45	93302.61
295.9323	Al3C3H7O5Na4	-0.66	93302.61
295.9323	Al5C4H10O3SNa	1.08	93302.61
295.9361	Al2C5H6O9S	1.06	95184.21
295.9361	Al5C5H9Na4	-1.85	95184.21
295.9481	AlC4H7O10PNa	-1.17	199175.9
295.9481	Al2C5H11O1PSNa4	-1.11	199175.9
295.9481	Al3C4H7O10	0.36	199175.9
295.9481	Al4C5H11O1SNa3	0.42	199175.9
295.9781	AlC5H13O4PSNa3	1.98	69054.82
295.9781	AlC4H10O6Na5	1.77	69054.82
296.9200	Al2C4H8O2PSNa4	0.45	820492.5
296.9200	Al4C4H8O2SNa3	1.97	820492.5
296.9291	Al3C4H9O6PS	-2.41	149673.6
296.9291	Al4C4H9O2PNa3	0.57	149673.6
297.9400	AlC5H6O8PNa2	-1.88	98096.83
297.9400	Al3C5H6O8Na	-0.36	98096.83
298.9088	Al2C5H3O8PNa	-0.18	521351.8
298.9088	Al3C3H7O7PS	-0.94	521351.8
298.9088	Al3C2H4O9Na2	-1.14	521351.8
298.9088	Al4C5H3O8	1.33	521351.8
298.9088	Al4C3H7O3PNa3	2.02	521351.8
298.9088	Al4C3H8SNa5	-1.08	521351.8
298.9240	Al3C4H8O1PNa5	-0.63	74692.03
298.9240	Al4C4H8O5SNa	-2.08	74692.03
298.9240	Al5C4H8O1Na4	0.88	74692.03
298.9269	Al2C5H4O7Na3	-2.14	299198.7
298.9269	Al3C3H7O9P	0.20	299198.7
298.9269	Al4C4H11PSNa3	0.26	299198.7
298.9269	Al4C3H8O2Na5	0.06	299198.7
298.9692	Al2C5H10O9P	1.42	153923.3
298.9692	Al2C5H11O6SNa2	-1.68	153923.3
298.9692	Al3C5H11O2Na5	1.28	153923.3
299.9409	Al2C4H7O10P	2.28	56434.86

299.9409	Al2C4H8O7SNa2	-0.81	56434.86
299.9409	Al3C5H11O1PSNa3	2.34	56434.86
299.9409	Al3C4H8O3Na5	2.14	56434.86
300.8534	Al4C3H0O5PNa2	-0.27	49146.47
300.8534	Al5C1H4O4PSNa	-1.02	49146.47
300.8534	Al5C0H1O6Na3	-1.22	49146.47
300.9035	AlC5H2O7PNa3	-2.04	398281.5
300.9035	Al3C5H2O7Na2	-0.54	398281.5
300.9035	Al3C3H6O2PNa5	0.15	398281.5
300.9035	Al4C3H6O6SNa	-1.29	398281.5
300.9035	Al5C4H9PSNa2	1.86	398281.5
300.9035	Al5C3H6O2Na4	1.65	398281.5
301.9798	AlC5H9O10Na2	-1.00	46552.66
302.8954	AlC1H3O10SNa3	1.34	39980.01
302.8954	Al3C1H5O8PNa2	-1.36	39980.01
302.8954	Al4C4H4O7P	1.08	39980.01
302.8954	Al4C4H5O4SNa2	-1.98	39980.01
302.8954	Al5C4H5Na5	0.94	39980.01
302.9513	AlC5H9O9PS	-0.60	52824.71
302.9513	Al2C5H9O5PNa3	2.32	52824.71
302.9513	Al2C5H10O2SNa5	-0.74	52824.71
302.9879	AlC5H10O10Na2	-0.09	70040.27
303.9443	AlC4H10O4PSNa4	1.62	303441.7
303.9955	AlC5H11O10Na2	-0.83	43306.56
304.9282	AlC5H8O2PSNa5	0.34	170187.6
304.9282	Al3C5H8O2SNa4	1.82	170187.6
304.9282	Al5C5H10PNa3	-0.87	170187.6
304.9398	Al2C4H9O3PNa5	-1.66	617527.9
304.9398	Al4C5H12O1PSNa2	0.03	617527.9
304.9398	Al4C4H9O3Na4	-0.17	617527.9
305.9751	AlC5H12O9PS	0.47	89584.43
306.9165	Al3C4H7O3PNa4	0.24	139317.2
306.9165	Al4C4H7O7S	-1.17	139317.2
306.9165	Al5C5H10O1PSNa	1.92	139317.2
306.9165	Al5C4H7O3Na3	1.72	139317.2
307.9780	AlC5H10O6Na5	1.38	107009.9
308.9623	AlC4H11O10PS	0.82	41091.7
310.8708	AlC0H1O10PNa4	-0.01	81214.34
310.8708	Al2C3H0O9PNa2	2.37	81214.34
310.8708	Al2C3H1O6SNa4	-0.61	81214.34
310.8708	Al3C1H4O8PSNa	1.64	81214.34
310.8708	Al3C0H1O10Na3	1.45	81214.34
310.8708	Al3C4H4PSNa5	2.43	81214.34
310.9090	AlC3H4O9PNa3	-1.91	282774.2
310.9090	Al3C4H7O7PS	-0.26	282774.2
310.9090	Al3C3H4O9Na2	-0.45	282774.2
310.9090	Al4C4H8SNa5	-0.40	282774.2
310.9209	Al2C3H6O10SNa	1.39	59024.08

310.9209	Al4C3H8O8P	-1.25	59024.08
311.9643	AlC4H11O5PNa5	0.17	212491
312.8975	AlC3H4O10PSNa	1.47	62741.79
312.8975	Al4C5H3O6Na2	-1.95	62741.79
312.8975	Al4C3H7O1PNa5	-1.29	62741.79
312.9661	Al2C5H9O9Na2	-2.00	88893.05
314.9640	Al2C5H10O10P	0.98	99335.26
314.9640	Al2C5H11O7SNa2	-1.96	99335.26
314.9640	Al3C5H11O3Na5	0.85	99335.26
315.9233	AlC5H6O10PS	1.20	220265.4
315.9233	AlC2H7O7PNa5	1.66	220265.4
315.9233	Al2C5H7O3SNa5	1.06	220265.4
315.9233	Al4C5H9O1PNa4	-1.53	220265.4
315.9354	Al2C4H8O8SNa2	-2.08	135164.7
315.9354	Al3C5H11O2PSNa3	0.91	135164.7
315.9354	Al3C4H8O4Na5	0.72	135164.7
315.9354	Al5C5H11O2SNa2	2.34	135164.7
316.8778	Al2C0H3O10PNa3	-1.33	45462.67
316.8778	Al3C3H2O9PNa	1.00	45462.67
316.8778	Al3C3H3O6SNa3	-1.93	45462.67
316.8778	Al4C4H6PSNa4	1.06	45462.67
316.8778	Al5C3H2O9	2.43	45462.67
316.9073	AlC5H1O10Na3	0.09	376163.7
316.9073	Al2C3H5O9SNa2	-0.63	376163.7
316.9073	Al3C4H8O3PSNa3	2.36	376163.7
316.9073	Al3C3H5O5Na5	2.17	376163.7
316.9199	AlC2H6O10PNa3	-0.81	220207.2
316.9199	Al2C5H5O9PNa	1.52	220207.2
316.9199	Al2C5H6O6SNa3	-1.41	220207.2
316.9199	Al3C3H9O8PS	0.80	220207.2
316.9199	Al3C2H6O10Na2	0.61	220207.2
316.9249	Al2C4H8O9PS	-0.93	68489.96
316.9249	Al3C4H8O5PNa3	1.86	68489.96
316.9249	Al3C4H9O2SNa5	-1.07	68489.96
316.9375	Al2C5H6O8Na3	-1.91	41112.54
316.9375	Al3C3H9O10P	0.30	41112.54
316.9796	Al2C5H12O10P	0.82	70294.45
318.9134	Al3C3H8O3PNa5	-1.94	117928
318.9134	Al5C4H11O1PSNa2	-0.33	117928
318.9134	Al5C3H8O3Na4	-0.52	117928
320.8909	AlC2H3O9PNa4	-1.99	49522.34
320.8909	Al2C5H2O8PNa2	0.32	49522.34
320.8909	Al3C3H6O7PSNa	-0.39	49522.34
320.8909	Al3C2H3O9Na3	-0.58	49522.34
320.8909	Al4C5H2O8Na	1.73	49522.34
320.8909	Al4C3H6O3PNa4	2.37	49522.34
320.8909	Al5C3H6O7S	1.02	49522.34
320.9292	Al3C5H6O8Na2	-2.11	1619470

322.9058	Al2C5H4O8PNa2	-2.01	314657.3
322.9058	Al4C5H4O8Na	-0.61	314657.3
322.9058	Al4C3H8O3PNa4	0.03	314657.3
322.9058	Al5C3H8O7S	-1.31	314657.3
322.9265	AlC3H9O6PSNa4	2.13	61766.5
322.9265	Al3C5H7O9P	-1.05	61766.5
322.9265	Al4C5H8O2Na5	-1.18	61766.5
322.9504	Al2C4H11O4PNa5	-1.46	57450.79
330.8946	AlC4H5O6PSNa4	0.26	32420.5
330.8946	Al2C1H5O10PNa3	2.20	32420.5
330.8946	Al3C4H5O6SNa3	1.63	32420.5
330.8946	Al5C4H7O4PNa2	-0.85	32420.5
332.8849	AlC5H1O9SNa3	1.42	23755.65
332.8849	Al3C5H3O7PNa2	-1.04	23755.65
332.8849	Al4C3H7O6PSNa	-1.72	23755.65
332.8849	Al4C2H4O8Na3	-1.90	23755.65
332.8849	Al5C5H3O7Na	0.32	23755.65
332.8849	Al5C3H7O2PNa4	0.94	23755.65
332.8940	AlC5H2O9PNa3	0.17	40502.94
332.8940	Al2C3H6O8PSNa2	-0.51	40502.94
332.8940	Al2C2H3O10Na4	-0.69	40502.94
332.8940	Al3C5H2O9Na2	1.53	40502.94
332.8940	Al3C3H6O4PNa5	2.15	40502.94
332.8940	Al4C3H6O8SNa	0.85	40502.94
333.9338	Al2C5H9O4SNa5	0.81	163016.3
333.9338	Al4C5H11O2PNa4	-1.64	163016.3
334.9179	Al2C3H7O10SNa2	-0.49	75511.69
334.9179	Al3C4H10O4PSNa3	2.34	75511.69
334.9179	Al3C3H7O6Na5	2.16	75511.69
334.9354	Al2C4H10O10PS	-1.08	298601.4
334.9354	Al3C4H10O6PNa3	1.56	298601.4
336.8649	Al2C3H3O7PSNa3	-1.92	23624.45
336.8649	Al2C2H0O9Na5	-2.10	23624.45
336.8649	Al4C3H2O10P	2.17	23624.45
336.8649	Al4C3H3O7SNa2	-0.58	23624.45
336.8649	Al5C4H6O1PSNa3	2.23	23624.45
336.8649	Al5C3H3O3Na5	2.05	23624.45
336.9119	Al2C4H9O3PSNa5	-1.41	205408.9
336.9119	Al4C4H9O3SNa4	-0.07	205408.9
336.9241	Al3C5H6O9Na2	-2.05	52557105
337.9053	AlC5H5O10PSNa	1.28	55752.22
337.9053	Al4C5H8O1PNa5	-1.27	55752.22
338.8892	AlC5H0O10Na4	-0.05	63409.37
338.8892	Al2C3H4O9SNa3	-0.71	63409.37
338.8892	Al3C4H7O3PSNa4	2.08	63409.37
338.9007	Al2C5H4O9PNa2	-1.96	6572692
338.9007	Al2C3H6O6SNa5	2.40	6572692
338.9007	Al4C5H4O9Na	-0.62	6572692

338.9007	Al4C3H8O4PNa4	-0.01	6572692
338.9007	Al5C3H8O8S	-1.29	6572692
338.9196	Al2C5H5O8Na4	-1.33	20362.3
338.9196	Al3C3H8O10PNa	0.74	20362.3
338.9196	Al5C3H8O10	2.08	20362.3
338.9283	AlC3H8O9SNa4	-1.98	89843.22
338.9283	Al2C4H11O3PSNa5	0.81	89843.22
338.9398	Al3C5H8O9Na2	-1.89	28321.3
338.9623	Al2C5H12O7SNa3	0.26	20808.18
339.9207	AlC5H7O10PSNa	0.54	20505.67
339.9207	Al3C5H7O10S	1.87	20505.67
339.9207	Al4C5H10O1PNa5	-1.99	20505.67
340.9047	AlC5H2O10Na4	-0.49	27194.68
340.9047	Al2C3H6O9SNa3	-1.15	27194.68
340.9047	Al3C4H9O3PSNa4	1.62	27194.68
341.9387	AlC4H9O7PNa5	0.80	23725.58
341.9387	Al3C5H12O5PSNa2	2.30	23725.58
341.9387	Al3C4H9O7Na4	2.12	23725.58
342.8717	Al4C5H1O8Na2	-1.72	38422.6
342.8717	Al4C3H5O3PNa5	-1.12	38422.6
342.8717	Al5C3H5O7SNa	-2.38	38422.6
343.9302	Al2C5H8O9SNa2	-2.25	25062.16
343.9302	Al3C5H8O5Na5	0.33	25062.16
346.8683	AlC1H2O10PNa5	-0.28	44820.6
346.8683	Al2C4H1O9PNa3	1.85	44820.6
346.8683	Al2C4H2O6SNa5	-0.82	44820.6
346.8683	Al3C2H5O8PSNa2	1.20	44820.6
346.8683	Al3C1H2O10Na4	1.03	44820.6
349.9113	Al3C4H7O10PNa	-0.64	22469.72
349.9113	Al4C5H11O1PSNa4	-0.59	22469.72
349.9113	Al5C4H7O10	0.65	22469.72
350.9130	Al4C3H9O9PNa	-2.27	163561.6
351.9112	Al3C5H5O10Na2	-1.94	61423.3
351.9112	Al3C3H9O5PNa5	-1.35	61423.3
354.8666	AlC5H0O9SNa4	0.64	35431.28
354.8666	Al3C5H2O7PNa3	-1.67	35431.28
354.8666	Al4C3H6O6PSNa2	-2.30	35431.28
354.8666	Al4C2H3O8Na4	-2.47	35431.28
354.8666	Al5C5H2O7Na2	-0.39	35431.28
354.8666	Al5C3H6O2PNa5	0.19	35431.28
354.8758	AlC5H1O9PNa4	-0.25	109048.6
354.8758	Al2C3H5O8PSNa3	-0.88	109048.6
354.8758	Al2C2H2O10Na5	-1.05	109048.6
354.8758	Al3C5H1O9Na3	1.03	109048.6
354.8758	Al4C3H5O8SNa2	0.39	109048.6
354.9105	AlC4H6O8PNa5	1.79	30550.9
354.9105	Al4C4H8O10P	-1.74	30550.9
354.9105	Al5C5H12O1PSNa3	-1.69	30550.9

354.9105	Al5C4H9O3Na5	-1.86	30550.9
354.9226	Al2C4H11O4PSNa5	-0.96	209446.7
354.9347	Al3C5H8O10Na2	-1.85	158850
355.9029	Al2C3H7O7SNa5	0.77	30819.14
355.9029	Al4C5H5O10Na	-2.11	30819.14
355.9029	Al4C3H9O5PNa4	-1.53	30819.14
355.9157	Al4C5H10O2PNa5	-1.67	66581.98
355.9272	Al3C5H12O3PSNa4	-1.18	38986.41
355.9272	Al5C5H12O3SNa3	0.09	38986.41
356.8872	Al4C4H7O3PNa5	-1.49	683419.4
356.8992	Al2C3H6O10SNa3	-2.26	222731.4
356.8992	Al3C4H9O4PSNa4	0.39	222731.4
356.8992	Al5C4H9O4SNa3	1.66	222731.4
356.9112	Al2C5H6O10PNa2	-2.04	25830.01
356.9112	Al2C3H8O7SNa5	2.10	25830.01
356.9112	Al4C5H6O10Na	-0.77	25830.01
357.8586	Al3C0H2O10Na5	2.48	19856.53
357.8586	Al4C5H0O9Na2	-2.18	19856.53
357.8586	Al4C3H4O4PNa5	-1.60	19856.53
357.8879	AlC3H4O9SNa5	1.28	38862.06
357.8879	Al3C3H6O7PNa4	-1.01	38862.06
357.8879	Al5C4H9O5PSNa	0.43	38862.06
357.8879	Al5C3H6O7Na3	0.26	38862.06
357.9041	Al3C4H7O9SNa2	-1.64	26985.63
357.9041	Al4C5H10O3PSNa3	1.00	26985.63
357.9041	Al4C4H7O5Na5	0.83	26985.63
358.8756	Al3C3H4O10SNa2	-1.47	37603.22
358.8756	Al4C4H7O4PSNa3	1.17	37603.22
358.8756	Al4C3H4O6Na5	1.00	37603.22
358.8847	Al2C4H6O6PSNa4	2.29	40150.75
358.8847	Al5C3H5O10Na	-1.37	40150.75
358.8878	AlC3H6O7PSNa5	2.17	135566.4
358.8878	Al3C5H4O10PNa	-0.69	135566.4
358.8878	Al4C3H8O9PS	-1.32	135566.4
358.8878	Al5C5H4O10	0.57	135566.4
358.8878	Al5C3H8O5PNa3	1.14	135566.4
359.9498	AlC4H11O8PNa5	2.25	22801.99
360.8829	Al2C5H3O9PNa3	-1.13	79295.71
360.8829	Al3C3H7O8PSNa2	-1.75	79295.71
360.8829	Al3C2H4O10Na4	-1.92	79295.71
360.8829	Al4C5H3O9Na2	0.12	79295.71
360.8829	Al4C3H7O4PNa5	0.70	79295.71
360.8829	Al5C3H7O8SNa	-0.50	79295.71
361.8899	Al4C5H4O9Na2	-2.16	22082.68
361.8899	Al4C3H8O4PNa5	-1.58	22082.68
362.8422	Al2C2H2O8PSNa4	-0.57	22249.17
362.8422	Al3C5H1O7PSNa2	1.47	22249.17
362.8422	Al4C2H2O8SNa3	0.67	22249.17

362.9305	Al3C5H10O7PNa3	1.95	21750.88
362.9305	Al3C5H11O4SNa5	-0.60	21750.88
364.9192	Al3C4H10O5PNa5	-0.82	42127.29
364.9192	Al4C4H10O9SNa	-2.01	42127.29
364.9192	Al5C5H13O3PSNa2	0.59	42127.29
364.9192	Al5C4H10O5Na4	0.42	42127.29
365.9001	Al4C3H8O10PNa	-2.15	22155.27
370.9001	Al2C3H7O8PNa5	2.35	39526.14
370.9001	Al5C3H9O10P	-1.03	39526.14
372.8648	Al2C5H0O9Na5	-2.17	101842.8
372.8648	Al4C4H7O2PSNa5	-0.24	101842.8
374.8979	Al4C4H9O4PNa5	-1.06	51488.16
374.8979	Al5C4H9O8SNa	-2.22	51488.16
375.8695	Al2C5H2O10PNa3	-2.39	24860.75
375.8695	Al4C5H2O10Na2	-1.18	24860.75
375.8695	Al4C3H6O5PNa5	-0.63	24860.75
375.8695	Al5C3H6O9SNa	-1.79	24860.75
376.8486	Al3C5H1O7PNa4	-1.42	22290.45
376.8486	Al4C3H5O6PSNa3	-2.02	22290.45
376.8486	Al4C2H2O8Na5	-2.18	22290.45
376.8486	Al5C5H1O7Na3	-0.22	22290.45
376.8577	AlC5H0O9PNa5	-0.35	64353.41
376.8577	Al2C3H4O8PSNa4	-0.95	64353.41
376.8577	Al3C5H0O9Na4	0.85	64353.41
376.8577	Al4C3H4O8SNa3	0.25	64353.41
376.8866	Al4C4H9O5PSNa3	2.27	39547.25
376.8866	Al4C3H6O7Na5	2.10	39547.25
376.8866	Al5C3H8O9PNa	-2.42	39547.25
376.9039	Al4C4H9O7PNa3	1.05	24874.95
376.9039	Al4C4H10O4SNa5	-1.41	24874.95
376.9169	AlC5H7O10PNa4	-2.26	19680.72
376.9169	Al3C5H7O10Na3	-1.06	19680.72
378.8926	Al4C5H5O10Na2	-2.16	244920.1
378.8926	Al4C3H9O5PNa5	-1.62	244920.1
378.9197	Al4C4H11O7PNa3	1.44	43175.99
379.9006	Al4C5H6O10Na2	-1.70	39928.27
380.8701	Al3C5H3O10PNa2	0.28	30338.14
380.8701	Al3C5H4O7SNa4	-2.15	30338.14
380.8701	Al4C3H7O9PSNa	-0.31	30338.14
380.8701	Al5C5H3O10Na	1.47	30338.14
380.8701	Al5C3H7O5PNa4	2.01	30338.14
380.9085	Al4C5H7O10Na2	-1.50	67938.26
393.8797	Al4C3H8O6PNa5	-1.53	140062.3
394.8973	Al4C3H8O8Na5	2.35	133846.6
394.9146	Al4C4H11O8PNa3	1.34	3579072
395.9154	Al2C5H7O10Na5	0.25	38583.3
398.8397	Al2C3H3O8PSNa5	-0.75	23994.28
398.8397	Al4C3H3O8SNa4	0.38	23994.28

410.8922	Al4C4H11O7PSNa3	2.37	324882
410.8922	Al4C3H8O9Na5	2.22	324882
416.8778	Al4C4H10O6PSNa4	-1.09	29804.28
418.8671	Al2C3H7O9PSNa5	2.11	40401.06
420.8609	Al5C3H8O6PNa5	-2.24	22467.55
442.8639	Al4C3H8O9SNa5	1.22	20227.12
458.8593	Al4C3H8O10SNa5	2.24	21984.44

Supplementary Table 7 Salt ion assignments in spectrum of biofilm grown on NGDPA for 24h.

m/z	Dev [ppm]	Assignment
78.9559	2.962422	H1O1NaK+
86.9482	3.42619	O3K+
87.9561	4.239633	H1O3K+
87.9771	4.488688	H1O4Na+
94.9297	1.020756	H1O1K2+
103.9508	1.51899	H1O4K+
104.9586	1.266215	H2O4K+
108.9425	-0.73525	O2PNa2+
120.9659	-1.78645	H3O4PNa+
124.9163	-1.73796	O2PNaK+
124.9373	-1.56158	O3PNa2+
125.9451	-1.74758	H1O3PNa2+
126.9529	-1.93063	H2O3PNa2+
136.9308	1.912649	H2O4SK+
142.8883	-0.31563	H2O0PSK2+
142.9092	-0.86139	H2O1PSNaK+
142.9384	-1.01512	H1O4SNa2+
148.9348	-1.94111	H1O3PNa3+
158.8831	-1.00766	H2O1PSK2+
163.9219	-1.70264	O4PNa3+
179.9168	-1.63464	O5PNa3+
179.9195	1.14996	H3O0P2Na5+
182.8902	-0.9246	H4O1P2NaK2+
182.9017	-0.72225	H3O2PSNa2K+
186.9117	-1.32736	O4PNa4+
188.9086	-1.64683	H1O5P2Na2+
196.868	-1.84438	O4SNaK2+
198.8756	-0.85028	H3O2PSNaK2+
198.9052	1.05025	H2O5SNa2K+
202.8855	-1.89811	O4PNa3K+
204.9035	-1.59148	H1O6P2Na2+
204.9222	-1.52789	H2O5PNa4+
206.9192	-1.33434	H3O6P2Na2+

212.8419	-1.8798	O4SK3+
212.8514	-1.95018	H1O4PK3+
214.8495	-0.95928	H3O2PSK3+
218.8595	-1.47172	O4PNa2K2+
220.8962	-1.1322	H2O5PNa3K+
222.9143	-0.40868	H3O7P2Na2+
226.8855	-1.19047	O6P2Na3+
228.9012	-0.96155	H2O6P2Na3+
228.9094	-1.15373	H1O8PNa3+
236.8701	-1.21206	H2O5PNa2K2+
242.8595	-0.85276	O6P2Na2K+
244.896	-1.36833	H2O7P2Na3+
246.92	-0.92783	H3O9PNa3+
250.8831	-1.05268	H1O6P2Na4+
262.9233	-0.33508	H2O12Na3+
266.8681	1.333619	H3O3P2Na5K+
266.8778	-1.7952	H1O7P2Na4+
268.8811	-0.35369	H2O8PNa3K+
278.8714	0.27934	H3O8PSNa2K+
282.8519	-1.11754	H1O7P2Na3K+
284.8588	1.575871	H2O7PSNa3K+
288.8598	-1.46472	O7P2Na5+
292.8981	1.815993	O14Na3+
298.8258	0.608716	H4O1P5SNa4+
298.8258	-1.18162	H1O7P2Na2K2+
300.8289	0.910485	H1O9P4S+
300.8289	1.216307	H5O2P4SNa3K+
300.8289	-0.56211	H2O8PNaK3+
300.8536	1.109843	H2O8PSNa3K+
302.8865	-0.88185	O17P+
303.8872	-0.25042	H3O12PSNa2+
304.8339	-0.85325	O7P2Na4K+
306.8599	-1.4505	O11P2Na3+
308.8757	-0.9554	H2O11P2Na3+
308.8757	-0.9554	H2O11P2Na3+
310.9087	1.823368	H2O15Na3+
314.7997	0.168043	O8P5S+
314.7997	0.460293	H4O1P5SNa3K+
314.7997	-1.2392	H1O7P2NaK3+
316.8029	1.35384	H5O2P4SNa2K2+
316.8029	-0.33491	H2O8PK4+
318.864	0.241169	O16PS+
320.8078	0.741566	H3O1P5SNa5+
320.8078	-0.9261	O7P2Na3K2+
323.8823	1.629916	H2O11PNa5+
324.8685	-0.36661	H3O10PNa4K+

324.879	-0.47433	H1O14PNa3+
328.834	-0.13715	O9P3Na4+
332.8014	0.780947	H2O8PSNaK3+
334.8344	0.877748	H3O11P4S+
336.7817	0.596529	H3O1P5SNa4K+
336.7817	-0.99204	O7P2Na2K3+
336.7817	-0.71886	H4O0P2Na5K4+
338.859	-1.02432	O12SNa5+
340.8464	-1.89557	H5O7P3Na4K+
340.8464	1.833381	H3O9PSNa4K+
346.8436	0.752213	O12PSNa4+
348.7753	0.639094	H2O8PSK4+
350.8083	1.581778	O7P4Na5+
356.8697	-0.59433	H2O13SNa5+
358.8845	-0.37366	H3O16PNa3+
364.842	-1.20902	H3O12P2Na3K+
364.8609	-0.87732	O18PNa2+
368.8267	0.395579	H1O10P3Na5+
372.8644	1.343386	H1O17PNa3+
372.8644	-1.14546	H2O14SNa5+
374.8619	0.293175	H3O15PSNa3+
380.8205	-1.20818	H1O16P4+
380.8205	-0.9666	H5O9P4Na3K+
380.8546	1.984751	H1O17Na3K+
380.8664	-0.46762	H2O16PNa4+
386.824	-0.99554	H2O12P2Na4K+
388.8421	-0.58147	H3O14P3Na3+
390.8095	-1.45109	H5O12P5K+
390.8095	1.801137	H3O14P3SK+
390.8751	1.626865	H3O18PNa3+
394.8464	1.410424	O17PNa4+
396.8495	1.866958	H1O18Na3K+
402.8029	-0.01018	O16P4Na+
402.8029	0.218221	H4O9P4Na4K+
404.8341	1.039686	H2O15SNa4K+
406.7819	-1.31545	O10P3Na5K+
406.7819	0.45946	H6O10P4K3+
412.8444	1.758293	H1O19Na3K+
424.7832	1.51348	O14P3Na3K+
424.7832	-0.67117	H1O11P2SNa5K+
426.8177	-0.50162	O17PSNa4+
426.8177	1.888163	H3O13P2Na5K+
434.7459	0.337899	H1O13P5SK+
434.7459	0.549517	H5O6P5SNa3K2+
434.7459	-0.68109	H2O12P2NaK4+

Supplementary Table 8 Lipid ion assignments in spectrum of biofilm grown on NGDPA for 24h.

m/z	Assignment	Lipid name
271.1881	C13H28O4Na+	'MG O-10:0;O'
299.2194	C15H32O4Na+	'MG O-12:0;O'
327.2507	C17H36O4Na+	'MG O-14:0;O'
794.4492	C40H69N1O11S1Na+	'SHexCer 34:5;O2'
767.412	C38H65O12PNa+	LPI 29:5/LPI O-29:6;O/PI O-29:5'
778.4751	C37H73N1O12S1Na+	'SHexCer 31:0;O3'
756.4934	C42H72N1O7PNa+	'LPC 34:7/PC O-34:7/PE O-37:7'
549.1587	C24H30O13Na+	'ST 18:4;O7;GlCA/ST 18:5;O8;Hex'
439.1428	C22H26N1O5S1Na+	'ST 22:6;O2;S'
512.1558	C21H31N1O10S1Na+	'ST 19:3;O8;T'
526.1708	C22H33N1O10S1Na+	'ST 20:3;O8;T'
549.1632	C24H32N1O10S1Na+	'ST 24:5;O7;S'
502.1709	C20H33N1O10S1Na+	'ST 18:1;O8;T'
453.1584	C23H28N1O5S1Na+	'ST 23:6;O2;S'
356.1508	C15H27N1O5S1Na+	'NAT 13:2;O'
599.3329	C29H53O9PNa+	'LPG 23:3/LPG O-23:4;O/PA 26:2;O/PG O-23:3'
325.235	C17H34O4Na+	'FA 17:0;O2/MG 14:0/MG O-14:1;O'
651.3732	C32H57N2O8PNa+	'PE-Cer 30:5;O4'
599.5006	C37H68O4Na+	'DG O-34:3/MG 34:3/MG O-34:4;O'
442.1666	C22H29N1O5S1Na+	'ST 20:5;O3;T'
510.209	C26H33N1O8Na+	'ST 18:5;O3;HexNAC/ST 24:6;O7;G'
430.1664	C21H29N1O5S1Na+	'ST 19:4;O3;T'
792.4334	C40H67N1O11S1Na+	'SHexCer 34:6;O2'
429.1584	C21H28N1O5S1Na+	'ST 21:4;O2;S'
385.0958	C18H20N1O5S1Na+	'ST 18:5;O2;S'
627.3734	C30H57N2O8PNa+	'PE-Cer 28:3;O4'
344.1508	C14H27N1O5S1Na+	'NAT 12:1;O'
393.1574	C18H28N1O5S1Na+	'ST 18:1;O2;S'
235.0944	C11H16O4Na+	'FA 11:3;O2'

Supplementary Table 9 Quinolone ion assignments in spectrum of biofilm grown on NGDPA for 24h.

m/z	Dev [ppm]	Assignment
173.107	-1.87225	C11H13N2+
182.0835	-1.91725	C12H10N2+
185.0706	-1.83227	C11H9N2O+
213.0655	-1.66193	C12H9N2O2+
219.0914	-1.25107	C15H11N2+
219.1039	-1.59787	C16H13N1+
220.1117	-1.70413	C16H14N1+

222.0909	-1.98162	C15H12N1O+
224.1066	-1.74069	C15H14N1O+
229.0882	-1.74649	C17H11N1+
231.1038	-1.94761	C17H13N1+
233.0831	-1.78091	C16H11N1O+
234.0909	-1.88004	C16H12N1O+
235.0987	-1.97831	C16H13N1O+
236.1066	-1.65222	C16H14N1O+
237.0761	0.32015	C13H12N1O2Na1+
240.0803	-1.97892	C18H10N1+
243.1039	-1.44012	C18H13N1+
244.1116	-1.94624	C18H14N1+
246.0909	-1.78836	C17H12N1O+
257.1071	-0.87162	C18H13N2+
257.2008	-1.6489	C17H25N2+
258.1848	-1.70459	C17H24N1O+
260.164	-1.94146	C16H22N1O2+
263.0811	-1.53603	C16H11N2O2+
270.1847	-1.999	C18H24N1O+
270.2213	-1.20309	C19H28N1+
272.2004	-1.80051	C18H26N1O+
282.2211	-1.86059	C20H28N1+
286.1796	-1.93969	C18H24N1O2+
286.2161	-1.53765	C19H28N1O+
296.2005	-1.31701	C20H26N1O+
298.0857	-1.86221	C20H12N1O2+
298.216	-1.8111	C20H28N1O+
300.2316	-1.96548	C20H30N1O+
301.0881	-1.66097	C23H11N1+
302.0959	-1.73819	C23H12N1+
304.1116	-1.56225	C23H14N1+
310.2161	-1.41869	C21H28N1O+
312.1953	-1.61789	C20H26N1O2+
312.2317	-1.56967	C21H30N1O+
314.2109	-1.76664	C20H28N1O2+
315.1037	-1.74577	C24H13N1+
316.2267	-1.28104	C20H30N1O2+
324.2317	-1.51157	C22H30N1O+
326.2473	-1.65549	C22H32N1O+
327.1036	-1.98744	C25H13N1+
328.1115	-1.75275	C25H14N1+
328.263	-1.49301	C22H34N1O+
330.1271	-1.89351	C25H16N1+
342.2423	-1.32976	C22H32N1O2+
354.2786	-1.5245	C24H36N1O+
359.1274	-1.83528	C24H18N1ONa1+

369.206	-0.8372	C24H28N1ONa1+
369.206	-1.67684	C20H31N2ONa1+
387.2162	-1.74088	C24H30N1O2Na1+
392.2019	1.784543	C24H29N2O+
393.1574	0.132008	C24H22N2O2Na1+
411.1111	1.707327	C26H16N2O2Na1+
525.3128	-0.56176	C33H45N1ONa1+

Supplementary Table 10 Saccharide / polysaccharide ion assignments in spectrum of biofilm grown on NGDPA for 24h.

m/z	Dev [ppm]	Assignment
374.0837	1.079706	C14H15N4O7Na+
374.0837	-0.77015	C8H21N3O10SNa+
386.0837	1.046147	C15H15N4O7Na+
386.0837	-0.74621	C9H21N3O10SNa+
400.0994	1.041992	C11H21N4O10P+
481.1106	-0.69028	C15H23N5O11S+
469.1106	-0.70793	C14H23N5O11S+
557.163	1.747966	C17H35N1O17S+
609.1627	-0.01166	C16H35N4O17PNa+
439.1428	-1.08871	C15H25N3O12+
450.1347	0.688461	C16H27O13Na+
558.1352	1.451084	C25H27O13Na+
398.0838	1.265815	C16H15N4O7Na+
398.0838	-0.47251	C10H21N3O10SNa+
427.1229	1.760386	C17H21N3O10+
427.1229	-1.38391	C19H23O11+
468.1028	0.224096	C22H21O10Na+
468.1028	1.614819	C14H25N2O12SNa+
410.0837	0.984922	C17H15N4O7Na+
410.0837	-0.70254	C11H21N3O10SNa+
453.0793	-0.50786	C17H21N1O10PNa+
482.1184	0.037128	C18H27O13P+
312.0802	-0.07082	C10H15N3O7Na+
633.1628	0.146724	C18H35N4O17PNa+
633.1628	-1.97437	C20H37N1O18PNa+
480.1027	0.010206	C23H21O10Na+
480.1027	1.366168	C15H25N2O12SNa+
465.0792	-0.70977	C18H21N1O10PNa+
465.0792	0.68999	C10H25N3O12PSNa+
388.0994	1.07421	C10H21N4O10P+
595.1434	0.204825	C28H25N3O12+
595.1434	1.29868	C20H29N5O14S+
595.1434	-0.95792	C22H31N2O15S+
579.1159	-0.17285	C32H21N1O10+

579.1159	0.951279	C24H25N3O12S+
